# Selective Chemical Inhibition of *agr* Quorum Sensing in *Staphylococcus aureus* Promotes Host Defense with Minimal Impact on Resistance

**DOI:** 10.1371/journal.ppat.1004174

**Published:** 2014-06-12

**Authors:** Erin K. Sully, Natalia Malachowa, Bradley O. Elmore, Susan M. Alexander, Jon K. Femling, Brian M. Gray, Frank R. DeLeo, Michael Otto, Ambrose L. Cheung, Bruce S. Edwards, Larry A. Sklar, Alexander R. Horswill, Pamela R. Hall, Hattie D. Gresham

**Affiliations:** 1 Research Service, New Mexico Veterans Affairs Medical Center, Albuquerque, New Mexico, United States of America; 2 Division of Infectious Diseases, Department of Internal Medicine, University of New Mexico School of Medicine, Albuquerque, New Mexico, United States of America; 3 Laboratory of Human Bacterial Pathogenesis, Rocky Mountain Laboratories, National Institute of Allergy and Infectious Diseases, National Institutes of Health, Hamilton, Montana, United States of America; 4 College of Pharmacy, University of New Mexico, Albuquerque, New Mexico, United States of America; 5 Department of Emergency Medicine, University of New Mexico, Albuquerque, New Mexico, United States of America; 6 Laboratory of Human Bacterial Pathogenesis, National Institute of Allergy and Infectious Diseases, National Institutes of Health, Bethesda, Maryland, United States of America; 7 Department of Microbiology, Dartmouth Medical School, Hanover, New Hampshire, United States of America; 8 Center for Molecular Discovery and Department of Pathology, University of New Mexico School of Medicine, Albuquerque, New Mexico, United States of America; 9 Department of Microbiology, Carver College of Medicine, University of Iowa, Iowa City, Iowa, United States of America; Vanderbilt University, United States of America

## Abstract

Bacterial signaling systems are prime drug targets for combating the global health threat of antibiotic resistant bacterial infections including those caused *by Staphylococcus aureus*. *S. aureus* is the primary cause of acute bacterial skin and soft tissue infections (SSTIs) and the quorum sensing operon *agr* is causally associated with these. Whether efficacious chemical inhibitors of *agr* signaling can be developed that promote host defense against SSTIs while sparing the normal microbiota of the skin is unknown. In a high throughput screen, we identified a small molecule inhibitor (SMI), savirin (*S. aureus* virulence inhibitor) that disrupted *agr*-mediated quorum sensing in this pathogen but not in the important skin commensal *Staphylococcus epidermidis*. Mechanistic studies employing electrophoretic mobility shift assays and a novel AgrA activation reporter strain revealed the transcriptional regulator AgrA as the target of inhibition within the pathogen, preventing virulence gene upregulation. Consistent with its minimal impact on exponential phase growth, including skin microbiota members, savirin did not provoke stress responses or membrane dysfunction induced by conventional antibiotics as determined by transcriptional profiling and membrane potential and integrity studies. Importantly, savirin was efficacious in two murine skin infection models, abating tissue injury and selectively promoting clearance of *agr*+ but not Δ*agr* bacteria when administered at the time of infection or delayed until maximal abscess development. The mechanism of enhanced host defense involved in part enhanced intracellular killing of *agr+* but not Δ*agr* in macrophages and by low pH. Notably, resistance or tolerance to savirin inhibition of *agr* was not observed after multiple passages either *in vivo* or *in vitro* where under the same conditions resistance to growth inhibition was induced after passage with conventional antibiotics. Therefore, chemical inhibitors can selectively target AgrA in *S. aureus* to promote host defense while sparing *agr* signaling in *S. epidermidis* and limiting resistance development.

## Author Summary

New approaches are needed to lessen the burden of antibiotic resistant bacterial infections. One strategy is to develop therapies that target virulence which rely on host defense elements to clear the bacteria rather than direct antimicrobial killing. Quorum sensing is a bacterial signaling mechanism that often regulates virulence in medically relevant bacterial pathogens. Therefore, drugs that inhibit quorum sensing can promote host defense by rendering the pathogenic bacteria avirulent and/or less fit for survival within the host. Our work addressed this strategy in the pathogen *Staphylococcus aureus* which is the major cause of acute bacterial skin and soft tissue infections. We conducted a high throughput screen to identify compounds that could inhibit signaling by the quorum sensing operon, *agr*. We found a compound that we termed savirin (*S. aureus* virulence inhibitor) that could inhibit signaling by this operon. The drug helped the innate immune system in animals to clear bacteria that express this operon without affecting clearance of bacteria that do not have this operon. We addressed the mechanism of action of this compound and whether resistance or tolerance to this compound would likely develop. Our data indicate for the first time that host defense against *S. aureus* skin infections can be enhanced by chemical inhibition of *agr*-mediated quorum sensing.

## Introduction

The global health threat of antibiotic resistant bacterial infections mandates rethinking of how antibiotics are used, how targets for new antibiotics are identified, and how mechanisms for promoting host defense can be enhanced [1], [2]. In this regard, there is much interest in chemical inhibition of bacterial signaling systems, particularly quorum sensing, because of its regulation of virulence in many medically relevant pathogens where antibiotic resistance is problematic [3], [4]. While chemical inhibitors of quorum sensing (QSIs) have been described in vitro, few have demonstrated *in vivo* efficacy [5]. Moreover, concerns have been raised about the specificity and selectivity of these compounds [6] as well as the potential for resistance development to quorum sensing inhibition [7]. Therefore, the future of quorum sensing inhibition as a medical strategy to replace or augment conventional antibiotics is uncertain.

Of the quorum sensing systems in Gram positive pathogens being targeted for chemical inhibition, the *agr* operon of *Staphylococcus aureus* has received noteworthy attention [3], [8]. This interest derives from its significant medical burden [9], its known propensity for developing resistance to newly introduced antibiotics [10], and the failure of all vaccines to date to prevent infection [11]. While chemical inhibitors of *agr* have been identified [8], none have demonstrated efficacy in mammalian models of infection. Moreover, none have demonstrated selectivity towards *agr* signaling in the pathogen *S. aureus* while sparing *agr* signaling in the skin commensal *Staphylococcus epidermidis*, an important contributor to host defense against skin infection [12].

Approximately 90% of *S. aureus* infections involve skin and soft tissues (SSTIs) [9], [13] and *agr* is positively associated with human SSTIs [14], [15]. Moreover, competitive interference with *agr* signaling is sufficient to abrogate experimental skin abscesses [16], and we have shown that innate immunity against experimental *S. aureus* skin infection requires active suppression of *agr* signaling [17]–[19]. Therefore, we postulated that selective chemical inhibition of *agr* signaling in *S. aureus* could promote host defense against SSTIs, providing evidence for limiting conventional antibiotic use in the majority of *S. aureus* infections. Here we describe a QSI identified in a high throughput screen that selectively inhibited *agr* signaling in *S. aureus*, but not in *S. epidermidis*, by blocking the function of the transcriptional regulator of the operon, AgrA, preventing upregulation of the *agr*-regulated genes essential for skin infection. It was efficacious in murine models of *agr*-dependent skin infection without apparent induction of resistance or tolerance after passage *in vivo*. These data provide proof-of-principle that AgrA transcriptional function in *S. aureus* can be selectively inhibited to attenuate quorum sensing with minimal toxicity to the bacterium or induction of stress responses observed with conventional antibiotics. Thus, selective AgrA blockade could enhance *agr*-dependent host defense in the skin while potentially preserving the normal microbiota, limiting resistance induction, and sparing conventional antibiotics for treatment of invasive systemic infections.

## Results

### A small molecule inhibitor, savirin, inhibits *agr* quorum sensing in *S. aureus* by blocking the transcriptional function of AgrA

**Figure 1:**
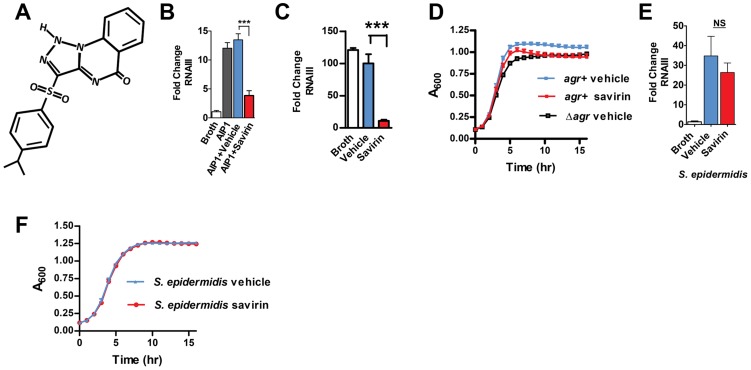
Savirin inhibits RNA III levels in *S. aureus*, but not *S. epidermidis*, without affecting *agr*-independent growth. (**A**) Chemical structure of savirin (3-(4-propan-2-ylphenyl) sulfonyl-1H-triazolo [1,5-a] quinazolin-5-one). Effect of savirin (5 µg ml^−1^) vs vehicle control on (**B**) RNAIII levels induced by 50 nM AIP1 at 1 hr in MRSA strain USA300 LAC; (**C**) RNAIII levels in LAC without exogenous AIP1 at 5 hrs; (**D**) growth of LAC compared to growth of LAC Δ*agr*; (**E**) RNAIII levels in *S. epidermidis* induced by overnight culture supernatant containing *S. epidermidis* AIP at 1 hr; and (**F**) growth of *S. epidermidis*. Data are represented as mean ± SEM, n = 3 experiments (**B**, **C**, **D**, & **F**) or n = 6 (**E**) performed in triplicate. ***p<0.001 **p<0.01, *p<0.05 by two-tailed Student's *t*-test.

The *agr* quorum sensing operon encodes two promoters [3], [20]; P2 that drives production of a two component sensor-regulator, AgrC and AgrA, and its autoinducing peptide pheromone ligand, and P3 that drives production of a regulatory molecule RNAIII that together with AgrA is responsible for transcriptional control of approximately 200 genes including multiple virulence factors and metabolic pathways involved in stationary phase growth [15]. P3 also drives P2 providing positive feedback to the production of the receptor (AgrC), the transcriptional regulator (AgrA), and the cyclic thiolactone peptide pheromone (AIP). Critically, the virulence factors most closely associated with human SSTIs, alpha hemolysin (hla), phenol soluble modulins (PSMs), and Panton-Valentine Leukocidin (PVL) are *agr* regulated [14], [15]. We screened 24,087 compounds selected for diversity for inhibition of AIP-induced *agr:*:P3 activation using a reporter strain where P3 drives production of GFP (ALC1743) (http://pubchem.ncbi.nlm.nih.gov/assay/assay.cgi?aid=1206&loc=ea_ras). We pursued one compound where dose-response experiments using an additional reporter strain indicated that it had minimal impact on exponential phase growth during the 3 hr assay starting at a CFU of 2×10^7^/ml and ending at ∼1×10^8^/ml. It inhibited optimally at 5 µg ml^−1^ (13.5 µM) (Fig. S1). Termed savirin (Fig. 1A), for *Staphylcoccus aureus* virulence inhibitor, its molecular weight (368) and lipophilicity (XLogP3-3.5) meet standards for drug development [21].

*S. aureus* isolates belong to one of four *agr* alleles depending on variations in AIP (amino acid sequence and length) and the cognate receptor, AgrC [3], [20]. While *agr* I alleles predominate in human disease, all four can contribute to SSTIs [9]. Therefore, an optimal chemical for *agr* disruption should work against all *agr* alleles. Savirin (5 µg ml^−1^) inhibited *agr::*P3 activation in reporter strains of each *agr* type (Fig. S2). Therefore, we pursued its efficacy *in vitro* and *in vivo* using a strain (LAC) of the epidemic methicillin-resistant USA300 clone and the predominant *agr* group I [15], [18], [19], [22]. We demonstrated by qRT-PCR that savirin (5 µg ml^−1^) inhibited both AIP1-induced RNAIII (Fig. 1B) and RNAIII produced at a longer time point without addition of exogenous AIP1 (Fig. 1 C) with no effect on exponential phase growth (Fig. 1 D). Stationary phase growth was negatively affected by both the genetic deletion of *agr* (Δ*agr*) and by savirin treatment (Fig. 1D) consistent with the known role of *agr* in regulating metabolic pathways of this growth phase in LAC [15]. Importantly, savirin did not significantly affect AIP1-induced RNAIII levels (Fig. 1E) or *agr*-dependent stationary phase growth (Fig. 1F) in the related Gram positive member of the skin microbiota, *S. epidermidis*. Nor did it affect growth of a Gram negative member of the skin microbiota, *Pseudomonas aeruginosa* [23] (data not shown). Because savirin could have different effects on growth in larger bulk cultures, we evaluated the effects of savirin on both exponential and stationary phase growth in 5 ml cultures diluted to measure OD_600_ under 0.8. The results were qualitatively similar (Fig. S3). In addition, savirin did not disrupt membrane integrity (Fig. S4A) or membrane potential (Fig. S4B), properties that are altered by antibiotic compounds that could affect *agr* signaling [24], [25] and that could be impaired by *agr*-independent, non-specific toxic effects [6].

**Figure 2:**
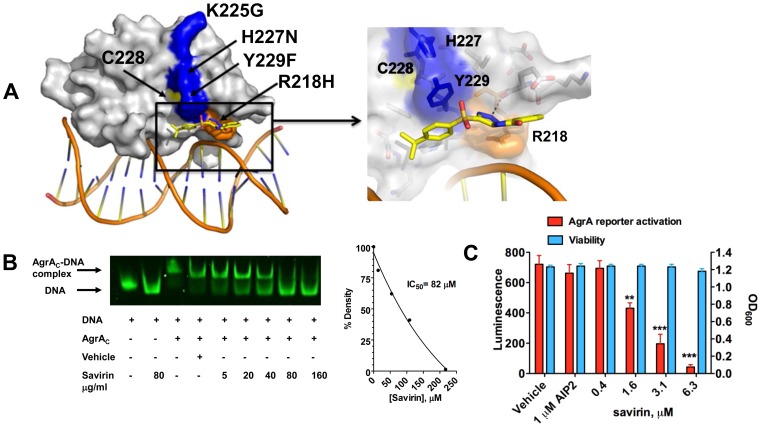
Savirin inhibits AgrA function in *S. aureus* both *in vitro* and within the organism. (**A**) *In silico* docking of savirin to AgrA_C_ from *S. aureus*. Space-filled representation of the C-terminal agrA DNA binding domain (gray) bound to target DNA. Surface residues that differ in *S. epidermidis* are blue, a naturally occurring dysfunctional mutation is shown in orange, and an essential Cys is shown in yellow. Savirin is shown in stick representation. An enlarged view of the boxed area shows the savirin docking site and surrounding residues. (**B**) Effect of increasing concentrations of savirin (5–160 µg ml^−^1 or 13.5–432 µM) vs vehicle on AgrA_C_-FAM labeled oligonucleotide complex formation by EMSA. The IC50 for savirin inhibition was quantified by densitometry of the bands. Data are representative of 3 independent experiments. (**C**) Effect of increasing concentrations of savirin (0.4–6.3 µM or 0.29–2.33 µg ml^−1^) vs vehicle on *agrA* reporter activation in an *agr* null strain expressing a plasmid for *agrA* where *agr*::P3 drives luminescence, AH3048, after 6 hr of growth. AIP2 as an inhibitor of non-*agr*II AgrC signaling was used as a specificity control. Viability is represented as OD_600_. Data are represented as the mean ± SEM of quadruplicates of a representative experiment of 3 independent experiments. ***p<0.001 **p<0.01, *p<0.05 by two-tailed Student's t-test.

To pursue the molecular mechanism by which savirin inhibits *agr* signaling in *S. aureus* but not in *S. epidermidis*, we examined the differences in histidine kinase function and transcriptional control between the two. Because residues within the histidine kinase domain of AgrC that are critical for *agr* activation are conserved between *S. aureus* and *S. epidermidis* [26], we pursued AgrA function as the molecular target of savirin. We used *in silico* docking of savirin to the C-terminal DNA binding domain (AgrA_c_) [27] of both *S. aureus* and *S. epidermidis* using the online server Swissdock [28]. Savirin docked to AgrA_c_ of *S. aureus* between Tyr229, which is adjacent to a residue critical for AgrA folding [29] (Cys228), and Arg218 near the DNA binding interface with a calculated binding energy of −6.1 kcal/mol (Fig. 2A). Notably, mutation of Arg218 to His has been described in clinical isolates with defective *agr* function [30]. At this position, savirin is within hydrogen bonding distance of the backbone carbonyl of Glu217 and within π-stacking distance of Tyr229 (Fig. 2A, enlarged view). Importantly, this site differed in *S. epidermidis* where the key Tyr229 is a Phe and His227 is an Asn. Consistent with this, attempts to dock savirin to this site in *S. epidermidis* were unsuccessful, demonstrating that the DNA binding domain of AgrA is the likely target of savirin. We performed electrophoretic mobility shift assays to prove that savirin blocked the DNA binding function of AgrA. Incubation of purified AgrA_c_ (2 µM) with the high affinity site in P2 and P3 (0.1 µM) (Fig. 2B) shifted electrophoretic mobility of the FAM labeled nucleotide and increasing concentrations of savirin (5–160 µg ml^−^1 or 13.5–432 µM) vs. vehicle inhibited this shift with an IC50 of 83 µM or 30.3 µg ml^−1^ (Fig. 2B). To prove that AgrA was the target within the pathogen, we constructed a novel reporter strain (AH3048) where plasmid-encoded AgrA constitutively produced without induction drives activation of *agr*::P3 *lux* in the absence of the rest of the *agr* operon, including *agrB*, *agrC*, and *agrD* [31]. As positive controls, we evaluated the ability of diflunisal and 4-phenoxyphenol, compounds published by others as inhibitors of AgrA_c_ DNA binding ability [27], [32] and both inhibited dose-dependently (Fig. S5). Additionally, increasing concentrations of savirin (0.4–6.3 µM or 0.29–2.33 µg ml^−1^) suppressed constitutive luminescence without affecting viability where AIP2, an inhibitor of non-*agr*II AgrC signaling [16], [20], had no effect on luminescence demonstrating that savirin specifically suppressed AgrA-dependent activation of P3 within the microorganism (Fig. 2C). In comparison to the positive control compounds (Fig. S5), savirin inhibited luminescence at 6.3 µM equivalent to the inhibition of the controls at 100 µM. Moreover, the concentration of savirin required for optimal inhibition of the agrA reporter was equivalent to the concentration that optimally inhibited *agr::P3* activation within the pathogen in strain LAC (1–5 µg ml^−^1)(Fig. 1). However, the concentration of savirin required to inhibit in the EMSA assay was much higher due to the excess of AgrA_c_ required for the optimal shift in electrophoretic mobility of the labeled nucleotide. Together, these mechanistic studies indicate that AgrA within *S. aureus* is savirin's molecular target.

### Savirin targets *agr*-dependent transcriptional regulation of major virulence factors implicated in SSTIs

**Figure 3:**
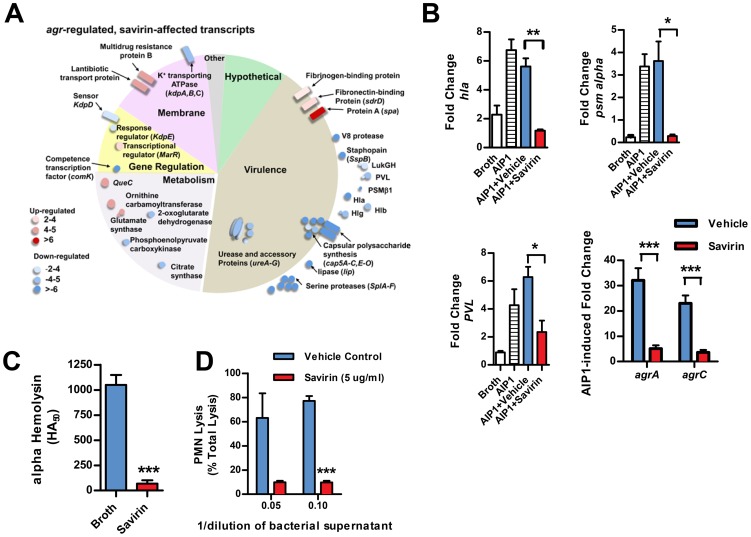
Savirin inhibits *agr*-regulated gene transcription and secreted virulence factor production. (**A**) Graphic representation by category of microarray results in LAC after 5 hrs of culture with 50 nM AIP1 and 5 µg ml^−1^ of savirin. Of 205 *agr*-regulated transcripts, 122 were affected by savirin by 2 fold or greater and p<0.05, n = 3. All major virulence factors are represented; others shown are representative of the category. (**B**) Effect of savirin (5 µg ml^−1^) on transcription induced by 50 nM AIP1 in LAC for *hla*, *psm alpha*, and *pvl* relative to 16S at 1 hr and for *agrA* and *agrC* at 5 hr. (**C**) Effect of savirin (5 µg ml^−1^) on overnight alpha-hemolysin production by LAC. (**D**) Effect of savirin (5 µg ml^−1^) vs. vehicle on the capacity of culture supernatant from a clinical *agr* I blood stream isolate to lyse human neutrophils as measured by LDH release after 2 hr. Data are represented as the mean ± SEM, n = 3 independent experiments. ***p<0.001 **p<0.01, *p<0.05 by two-tailed Student's *t*-test.

**Figure 4:**
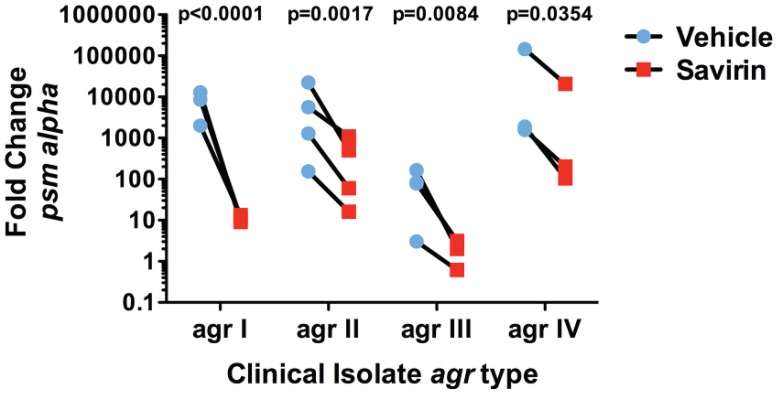
Savirin inhibits AgrA-dependent transcription in clinical isolates. Effect of savirin (5 µg ml^−1^) vs. vehicle on *psm alpha* transcripts determined by qRT-PCR in clinical *S. aureus* isolates of each *agr* allele after 5 hr of culture. Data are represented as the mean of 5 replicates. Significance determined by two-way repeated measures ANOVA.

We investigated the transcriptional impact of savirin on *agr* virulence by microarray analysis [15], [33] and confirmed the results by qRT-PCR and direct measurement of virulence factor function in LAC and in multiple clinical isolates. All of these were performed with the same concentration of savirin, 5 µg ml^−1^. The effect of savirin vs. vehicle on AIP1 induced transcription in LAC was compared to the differences between LAC and Δ*agr* LAC. Two hundred and five non-redundant transcripts were different and changed by greater than two fold between LAC and Δ*agr* LAC (Table S1). Of these, savirin affected 122 or 60% of *agr*-regulated transcripts by a similar magnitude and direction including downregulation of *agr* secreted virulence factors (the majority of transcripts affected), transcriptional regulators, and metabolic pathways important for SSTIs [14], [15] (Fig. 3A). Of the remainder of the potentially *agr*-regulated transcripts not affected by savirin, the majority were hypothetical or involved in metabolism. In contrast, savirin affected only 5% of the non *agr*-regulated transcriptome (Table S1) demonstrating selectivity towards *agr*-dependent transcription. The transcripts upregulated by savirin in both LAC and Δ*agr* LAC could reflect a stress response or be implicated in resistance or tolerance induced by savirin exposure. Of the 19 transcripts upregulated, only 5 with potential roles in drug efflux or resistance were significantly affected (Table S2). However, this did not include the two most closely implicated with antibiotic resistance, *norA* (SAUSA300 0680) or *mecA* (SAUSA300 0032). Importantly, transcripts were not affected for known stress response genes and the anti-inflammatory exotoxins induced by bactericidal agents [24], [34]–[36] or *agr* ablation [25], [37] (Table S1). We confirmed by qRT-PCR that savirin inhibited AIP1 induced transcripts for RNAIII and AgrA regulated genes including *hla*, *psm alpha*, *pvl (lukS)*, *agrA*, and *agrC*, (Fig. 3B). We also confirmed by qRT-PCR that the anti-inflammatory exotoxin set7 was not affected by savirin (fold increase of vehicle 4.66±1.47 SEM vs 4.66±0.7 SEM for savirin, n = 3). Alpha hemolysin activity (Fig. 3C) and PMN lysis capacity (Fig. 3D) in savirin-treated bacterial supernatants were inhibited as well as lipase and protease activity (data not shown). Moreover, savirin inhibited *psm alpha* transcripts in clinical isolates of all four *agr* alleles (Fig. 4). Additionally, savirin reduced alpha hemolysin activity in supernatants from numerous MRSA and MSSA clinical isolates from multiple sites of infection (Fig. S6).

### Savirin treatment *in vivo* attenuates tissue injury and promotes *agr* but not *Δagr* bacterial clearance

**Figure 5:**
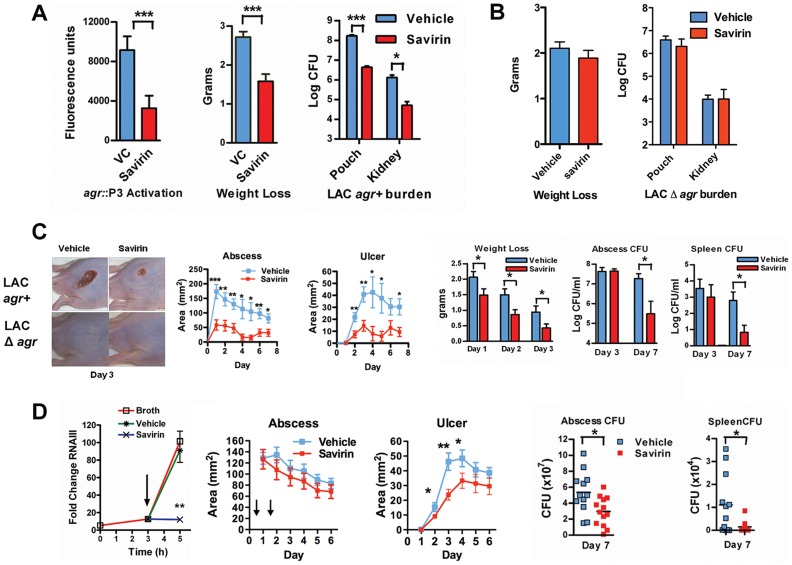
Savirin promotes *agr*-dependent host defense *in vivo* and *in vitro*. (**A**) Effect of 10 µg of savirin vs. vehicle on infection with 2×10^7^ LAC AH 1677 (*agr*::P3 driving *yfp*) of *Nox2^−/−^* mice (n = 8 per group) in an air-pouch model 24 hrs after infection. Parameters shown include the fluorescence of bacteria in a lavage of the pouch, weight loss, and bacterial burden in the pouch and in the kidney. (**B**) Effect of 10 µg savirin vs vehicle on infection with 5×10^7^ LAC Δ*agr* of wild-type C57BL/6 mice (n = 4 per group) in an air-pouch model 24 hr after infection. Parameters shown include weight loss and bacterial burden in a lavage of the pouch and kidney. (**C**) Effect of savirin (5 µg) vs vehicle injected at the time of infection with 4×10^7^ LAC *agr*+ or Δ*agr* subcutaneously in the flank (n = 10–15 mice per group) of immunocompetent hairless SKH1 mice. Parameters shown include images of the infection sites at day 3; abscess area for *agr*+ infected mice; ulcer area for *agr*+ infected mice; weight loss over 3 days for *agr*+ infected mice; bacterial burden measured as CFU from the skin of *agr*+ infected mice at days 3 and 7; and bacterial burden measured as CFU from the spleen of *agr*+ infected mice at days 3 and 7. (**D**) Effect of delayed addition of savirin *in vitro* and *in vivo*. Parameters shown include the *in vitro* effect of savirin (5 µg ml−1) added at 3 hrs on RNAIII levels at 5 hr. **p<0.01 by two tailed Student's *t* test and savirin (5 µg) vs. vehicle injected 24 and 48 hrs after infection (n = 12–13 mice per group), depicting abscess area (arrows indicate timing of savirin/vehicle injection), ulcer size, bacterial burden in the skin at day 7, and bacterial burden in the spleen at day 7. All data from mouse infection represented as mean ±SEM ***p<0.001 **p<0.01, *p<0.05 by two-tailed Mann-Whitney U test.

Given this SMI's selective effect on virulence factor production by multiple isolates, we pursued savirin's *in vivo* efficacy in two murine models of skin and soft tissue infection. To confirm that savirin inhibited *agr* signaling *in vivo* and that it did not affect infection with LAC Δ*agr*, we used an airpouch skin infection model. Mice genetically deficient in the NADPH oxidase (*Nox2^−/−^*) lack control of *agr::*P3 activation in this model causing maximal *in vivo* quorum sensing [17]–[19]. The airpouch in the skin was infected with LAC expressing a fluorescent reporter of *agr::*P3 activation (AH1677) [19] and savirin (10 µg) was co-administered at the time of infection. Savirin treatment significantly inhibited *agr::*P3 activation in bacteria from a lavage of the pouch as well as consequential weight loss (as a measure of morbidity) and bacterial burden in the pouch lavage and systemically in the kidney (Fig. 5A). Moreover, when C57BL/6 mice were infected with LAC Δ*agr* using the same model, savirin (10 µg) did not affect weight loss or bacterial burden in the pouch lavage or the kidney (Fig. 5B). These *in vivo* data are consistent with our *in vitro* data demonstrating that savirin selectively inhibits *agr* activation and that it has minimal impact on bacteria lacking *agr*.

In addition, we evaluated savirin in an established model of *agr*-dependent dermonecrotic skin infection in hairless immunocompetent mice [15], [38]. In this model, clearance of Δ*agr* LAC vs. LAC was enhanced by day 7 (Fig. S7) demonstrating that *agr* contributes not only to early tissue injury [15], [38] but to persistence in the skin. Subcutaneous injection of savirin (5 µg) vs. vehicle at the time of infection abrogated abscesses and dermonecrosis (measured as area of ulceration) (Day 1–3) (Fig. 5C) similarly to the genetic deletion of *agr* (Fig. 5C, images) and prevented early morbidity (measured as weight loss). At day 3 the bacterial burden in the skin abscess was unaffected by savirin treatment (Fig. 5C), indicating that savirin inhibited toxin-induced tissue injury and not bacterial viability at this time point. In contrast, at day 7 savirin treatment promoted bacterial clearance from abscesses and systemically from the spleen (Fig. 5C), replicating the phenotype of *agr* deletion (Fig. S7). Because ongoing quorum sensing is likely as the pathogen reaches the required density in discrete locales to accumulate AIP and activate AgrC, we examined the effect of delayed delivery of savirin both *in vitro* and *in vivo*. Delayed delivery inhibited RNAIII production *in vitro*, dermonecrosis *in vivo*, and promoted bacterial clearance from the skin and systemically from the spleen at day 7 (Fig. 5D).

**Figure 6:**
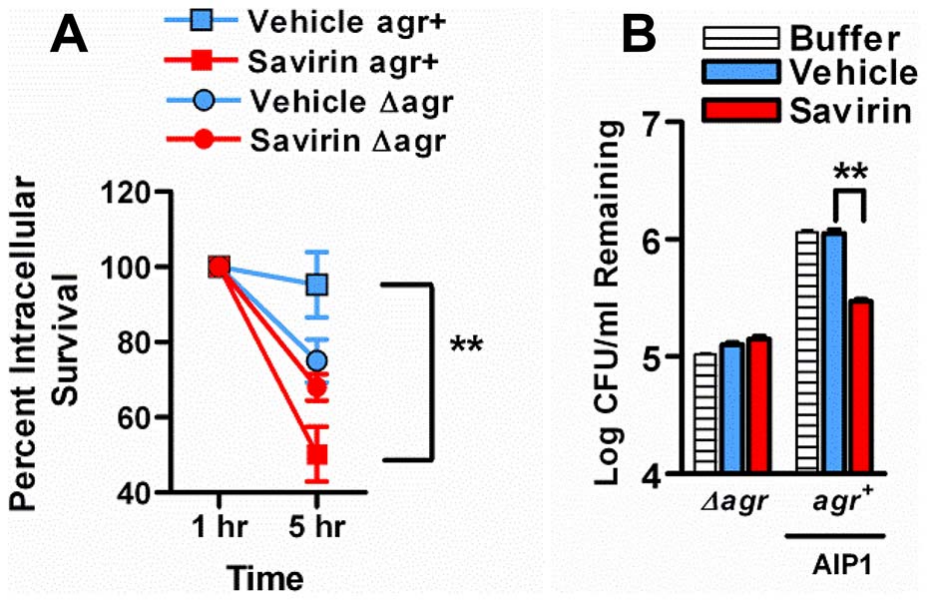
Effect of savirin treatment on *in vitro* host-dependent killing of LAC *agr*+ and Δ*agr*. (**A**) Percent intracellular survival of LAC *agr*+ (plus AIP1) or Δ*agr* treated with savirin (5 µg ml^−1^) vs. vehicle for 5 hr prior to opsonization and phagocytosis by mouse macrophages (MOI 1∶1). Viable intracellular CFU set at 100% after internalization for 1 hr. Mean ± s.e.m., n = 3 independent experiments performed in triplicate. (**B**) Log CFU remaining of 1.0×10^8^ LAC *agr*+ (plus AIP1) or Δ*agr* treated with savirin (5 µg ml^−1^) vs. vehicle for 5 hr prior to incubation at pH 2.5 for 2 hr. Mean ± SEM, n = 6. ***p<0.001 **p<0.01, *p<0.05 by two-tailed Student's *t*-test.

These data indicate that savirin promoted bacterial clearance not by inducing non-specific, *agr*-independent toxicity in the bacteria, because it did not lead to a reduction in CFU of Δ*agr* at 24 hr (Fig. 5B) or of LAC *agr*+ at 3 days (Fig. 5C), but by rendering LAC less able to survive within the skin leading to clearance by skin host defense mechanisms during the resolution of the infection (Fig. 5 C, D) (Figs. S7). Skin host defense mechanisms are comprised in part of phagocytes, antimicrobial peptides, lytic lipids, and an acidic environment [39]–[41]. Given the time frame that clearance was enhanced, we postulated that savirin treatment of LAC (5 µg ml^−1^) but not Δ*agr* LAC would augment killing of the bacteria in vitro by macrophages. As predicted, survival of vehicle treated LAC intracellularly from 1–5 hrs was significantly greater than savirin treated LAC (Fig. 6 A). In contrast, savirin had no effect on the intracellular survival of LAC Δ*agr* (Fig. 6A) indicating that savirin's effect on intracellular viability was *agr*-specific. Because optimal killing of *S. aureus* within macrophage phagolysosomes requires acidification [42] and because *agr* regulates transcripts involved in acid resistance [43] (urease, *kdpDE*, Fig. 3A), we incubated savirin- and vehicle-treated LAC and LAC Δ*agr* at pH 2.5 and evaluated viability. As with survival inside macrophages, savirin treatment promoted killing of *agr+* but not Δ*agr* bacteria (Fig. 6B). Of interest in both of these assays, the vehicle treated Δ*agr* bacteria were more easily killed compared to the vehicle treated *agr+* bacteria indicating that *agr* contributes to survival inside macrophages and to acid resistance (Fig. 6A,B). However, savirin treatment did not enhance killing by the antimicrobial peptide beta defensin 3, reactive oxidants, or lytic lipids (data not shown) indicating that savirin enhanced killing by some but not all skin defense mechanisms. These data suggest that enhanced killing by macrophages or the acidic environment of the skin may contribute in part to the ability of savirin to promote clearance of *agr*+ bacteria from the skin.

### Passage of *S. aureus* with savirin minimally impacts resistance

**Figure 7:**
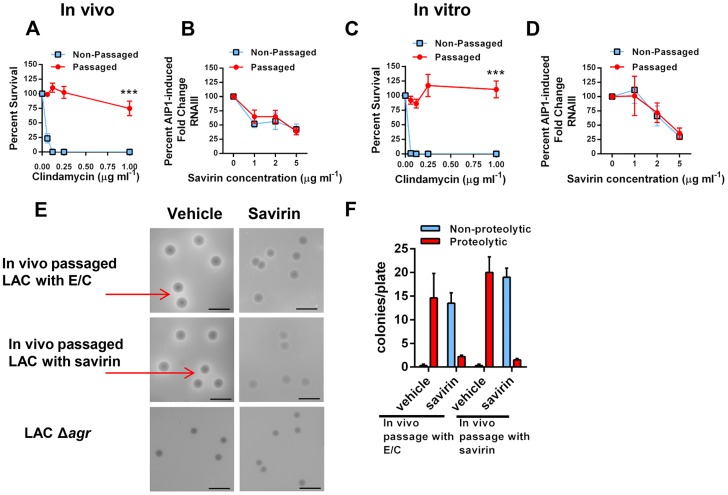
Passage of *agr*+ LAC with savirin *in vivo* or *in vitro* does not induce resistance or tolerance to savirin inhibition of *agr* signaling. (**A** & **B**) In vivo passage of LAC sequentially through the skin of 10 individual mice for 24 hr in the presence of either 16 µg erythromycin and 0.12 µg clindamycin (**A**) or 5 µg savirin (**B**). (**A**) Percent survival after incubation overnight with 16 µg ml^−1^ erythromycin and increasing concentrations of clindamycin of non-passaged and passaged LAC, mean ± SEM, n = 3. (**B**) Percent AIP1 induced fold change in RNAIII after incubation for 1 hr with increasing concentrations of savirin in non-passaged or passaged LAC, mean ± SEM, n = 4–5. (**C** & **D**) *In vitro* passage serially for 10 days of LAC with either 16 µg ml^−1^ of erythromycin and 0.12 µg ml^−1^ clindamycin (**C**) or 5 µg ml^−1^ savirin (**D**). (**C**) Percent survival after incubation overnight with 16 µg ml−1 erythromycin and increasing concentrations of clindamycin of non-passaged and passaged LAC, mean ± SEM, n = 3. (**D**) Percent AIP1 induced fold change in RNAIII after 1 hr incubation with increasing concentrations of savirin in non-passaged and passaged LAC, mean ± SEM, n = 3. ***p<0.001 by two-tailed Student's *t*-test. (**E** & **F**) Assessment of savirin resistance at the colony level of LAC passaged *in vivo* with either antibiotics (E/C) or savirin. (**E**) In vivo passaged bacteria were plated on skim milk agar plates (diluted to give ∼15–20 colonies/plate) containing either vehicle or 10 µg ml^−1^ savirin for 72 hr. Non-passaged LAC Δ*agr* is shown for comparison. Arrows are pointing to zones of proteolysis. The black bar is 5 mm. (**F**) Quantification of proteolytic and non-proteolytic colonies after 72 hr on milk agar plates containing either vehicle or savirin. Data are represented as mean ± SEM, n = 8 replicates of a representative experiment of two independent experiments.

*S. aureus* has a remarkable propensity for developing resistance or tolerance to antibiotics [10] but whether it would become resistant to inhibition of quorum sensing, as has been postulated for Gram negative bacteria [7], is unknown. Resistance or tolerance to savirin suppression of quorum sensing could occur by either selecting for the survival of spontaneously arising *agr* dysfunctional mutants or by stimulating drug efflux necessitating higher concentrations of savirin for efficacy. To be clinically significant, resistance or tolerance induced by repeated exposure should occur *in vivo*. To address this, we serially passaged LAC with savirin (5 µg) vs. vehicle sequentially through the skin of ten individual mice 24 hrs after infection. We compared this to *in vivo* passage with sub-inhibitory concentrations of antibiotics known to induce resistance in USA300 strains, erythromycin and clindamycin, because of the genetic expression of ermC [44]. We chose clindamycin as a control because it is used clinically for the treatment of SSTI's and emergence of resistance to clindamycin is clinically important [44]. Passage *in vivo* with conventional antibiotics induced resistance to killing by clindamycin (Fig. 7A) but passage with savirin did not affect its ability to inhibit *agr* signaling in the savirin passaged bacteria, as measured by AIP1 induction of RNAIII by qRT-PCR, or the dose response of savirin optimal for inhibition of RNAIII production (1–5 µg ml^−^1) (Fig. 7B). Equivalent data were obtained with *in vitro* passage every day for ten days (Fig. 7C, D).

To address resistance at the colony level, we plated the *in vivo* passaged bacteria on milk agar plates where proteolysis is *agr*-dependent and contributes to colony growth (Fig. 7E). While passage of *S. aureus in vitro* leads to the production of *agr* dysfunctional colonies [45], whether this happens with *in vivo* passage is unknown. The passaged bacteria were diluted to give 15–20 colonies per plate, spread on plates containing either vehicle or 10 µg ml^−1^ savirin, and proteolytic and non-proteolytic colonies enumerated at 72 hr. Both antibiotic- and savirin- passaged bacteria plated on vehicle had equally large colonies with clear zones of proteolysis (≥1.0 mm) and neither had small non-proteolytic colonies indicative of *agr* dysfunction (Fig. 7E, F). In contrast, when both the savirin and antibiotic passaged bacteria were plated on savirin containing plates, the majority of the colonies converted to a non-proteolytic phenotype however a small number had zones of proteolysis ≥1.0 mm (Fig. 7E,F). These data demonstrated that plating on savirin was able to suppress *agr*-dependent protease production and that there was no difference between antibiotic- and savirin- *in vivo* passaged bacteria in their sensitivity to savirin inhibition. In total, these data indicate that under conditions where resistance to growth inhibition can be induced *in vivo* with a conventional antibiotic used for treatment of SSTI's, savirin exposure did not lead to loss of *agr* function or tolerance to savirin inhibition of *agr* function at both the population and colony level.

## Discussion

**Figure 8:**
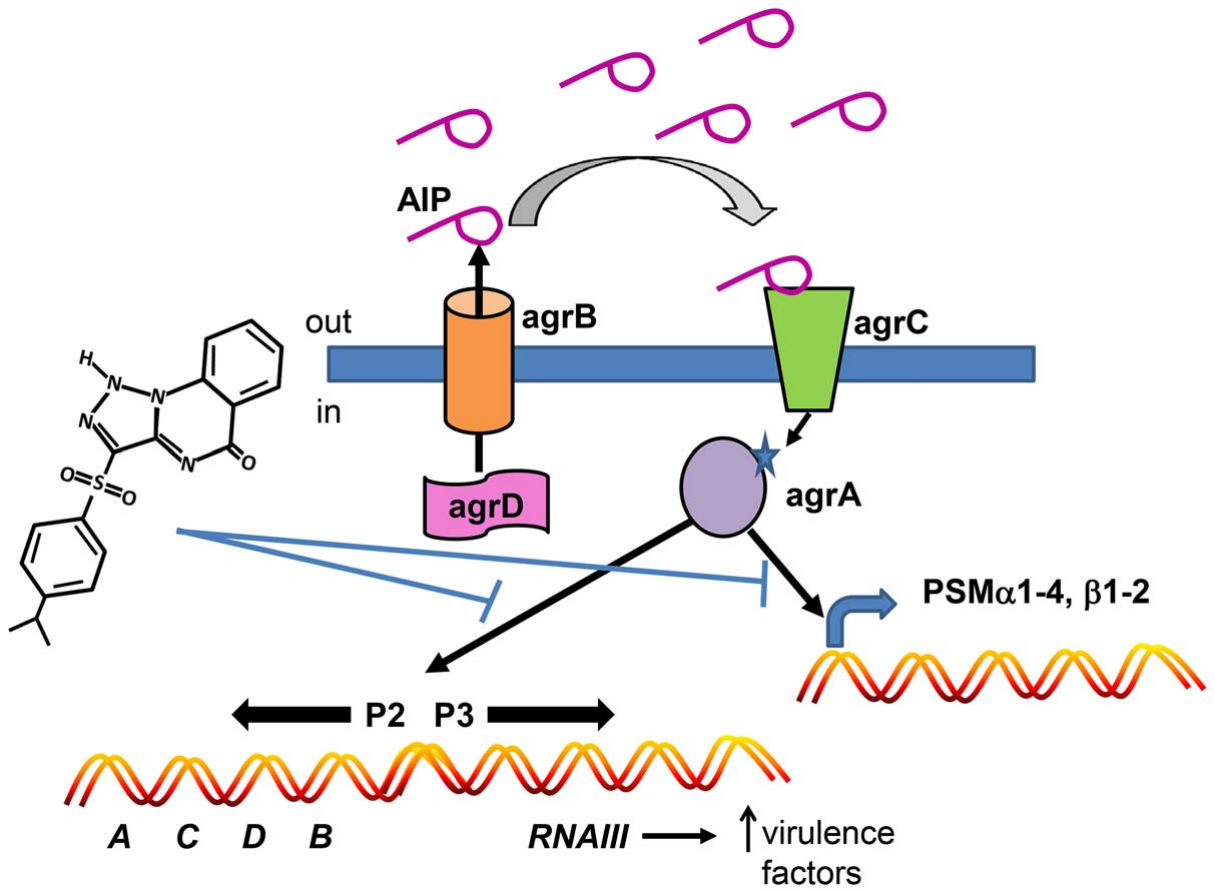
Model depicting the mechanism of action of savirin on *S. aureus* virulence. Savirin blocks AgrA binding to its promoter sites subsequent to AIP secretion, binding, and signaling through AgrC. This blocking prevents the positive stimulation of P2 preventing increased AgrA and AgrC expression, the production of RNAIII which alters expression of multiple secreted virulence factors, and the direct promoter function of AgrA on PSM production.

In this work we have used an SMI as a tool to address many of the concerns raised about the use of quorum sensing inhibitors as therapies or adjuncts for the prevention or treatment of antibiotic resistant bacterial infections [6], [7]. We identified an SMI in a high throughput screen that inhibited signaling of the *agr* quorum sensing operon in the medically significant pathogen, *S. aureus* (Fig. 8 model). We addressed the specificity of the inhibitor for *agr* signaling in this pathogen, its lack of generalized non-specific, *agr*-independent toxic effects on the bacterium, its molecular mechanism of action, its selective efficacy *in vivo*, and the potential for resistance development. If QSIs are to be efficacious for treating bacterial infections, they must work by enhancing host defense against the pathogen rendered either avirulent by the inhibitor or less fit for survival within the host. The evidence that our SMI works this way rather than by some non-specific, *agr*-independent toxicity on the bacterium *in vivo* includes: 1) its lack of effect on the number of Δ*agr* bacteria 24 hr after infection, 2) its lack of effect on the number of *agr*+ bacteria early (day 3) at the site of skin infection, and 3) its lack of effect on macrophage or low pH killing of Δ*agr* bacteria. Moreover, the reduction in CFU observed in our 2 models of SSTIs both at the site of infection and systemically (1.5–2.0 logs) was similar to that seen with conventional antibiotics tested in a murine surgical wound infection model [46] suggesting that if drugs were developed as QSIs with adequate bioavailability and pharmacokinetic properties that the eventual reduction in bacterial number could approach that seen with currently used antibiotics.

Because the majority of *S. aureus* infections involve skin and skin structures and are dependent on *agr* signaling in humans and animals [9], [14], [15], limiting antibiotic use in these infections could have a major impact on preserving conventional antibiotics for systemic, life-threatening infections [47]. In this regard, a clinical trial is ongoing which is testing whether treatment of uncomplicated skin abscesses could be limited to incision and drainage without systemic antibiotic use (NCT00730028, Uncomplicated Skin and Soft Tissue Infections Caused by Community-Associated Methicillin-Resistant *Staphylococcus aureus*). Our data are consistent with this approach and suggest that a QSI could either substitute for or be used as an adjunct to conventional antibiotics in this setting. Additionally, a QSI could be substituted for antibiotics used prophylactically to prevent wound infections until clinical signs of infection were apparent. Whether a QSI like savirin could be an adjunct with conventional antibiotics for treating systemic infections with or without a biofilm component is a matter of speculation and was not addressed by our studies. In fact, QSI's may have very different clinical utility in Gram negative and positive infections. Because even appropriate antibiotic use drives resistance [1], any strategy that spares conventional antibiotic use could positively impact resistance development. However, more work is needed in understanding the host defense status of patients presenting with acute bacterial skin infections because the effective use of a QSI is dependent on patients having adequate host defense systems to clear the QSI-treated, less virulent and/or less fit pathogen. Intriguingly, our compound was efficacious in mice lacking the Nox2 phagocyte oxidase, an important component of host defense against *S. aureus* in humans [17]–[19], suggesting that *agr* inhibitors may have efficacy in some patients with impaired host defense systems. More experimental work is required to determine which host defense elements are essential for *agr* inhibitor efficacy.

The potential for resistance development to QSI's has been addressed primarily in Gram negative bacteria (particularly *Pseudomonas aeruginosa*) where QS mutants (cheaters) arise during infection by taking advantage of the metabolic effort exerted by QS enabled bacteria for survival [7]. Whether this happens even experimentally *in vivo* with *S. aureus* infection is uncertain. Based on studies in Gram negative bacteria [7], the use of a QSI like savirin could give rise to mutants with a selective advantage over wild-type organisms. However, given the mechanism of action of savirin and its potential binding site in AgrA_c_, mutants resistant to savirin are most likely to be *agr* dysfunctional. Mutations in either *agrA* or *agrC* do arise in human infection [30] and savirin's potential binding site includes a known mutation in *agrA* (Arg 218 to His) [30]. However, elegant epidemiologic investigation has determined that these arise primarily from colonizing strains prior to the initiation of infection and not spontaneously from *agr* enabled bacteria during the course of infection [48]. Moreover, these mutants are less fit for transmission between patients [30], [48] suggesting that even if *agr* mutants arise with savirin exposure, they are unlikely to have a selective advantage over wild-type bacteria. Importantly, infection with *agr* mutants is primarily associated with bacteremia in hospitalized patients with impaired host defense systems and not with acute skin infection in immunocompetent individuals [14], [48]. This information along with our experimental data with *in vivo* passage in mice suggests that *agr* inhibitors may not drive the selection of *agr* mutants in skin infection. However, resistance or tolerance to *agr* inhibitors could arise by inducing a survival response in the bacteria that leads to upregulation of efflux mechanisms. Our microarray data suggest this as a possibility but neither *in vivo* nor *in vitro* passage with savirin resulted in resistance or tolerance to *agr* inhibition at either the population or colony level under the conditions we used. Currently, it is impossible to predict whether these issues would arise in human infection and whether our method for chronic exposure with *in vivo* passage in mice actually reflects how skin bacteria would be exposed to a QSI during human infection.

The mechanism of action of our SMI suggests that focusing on a site for targeted drug development within the DNA binding domain of the transcriptional regulator AgrA that is different between S. *aureus* and *S. epidermidis*, would be optimal for creating an *agr* inhibitor that spares the important contribution of *S. epidermidis* to host defense against skin infection [12], [23]. However, additional work is required to prove that savirin binds directly to the proposed site in AgrA_c_ and to prove that savirin does not affect skin colonization by *S. epidermidis*. Other investigators have reported compounds that inhibit AgrA DNA binding but whether these compounds would also inhibit in *S. epidermidis* was not addressed [27], [32]. Our novel AgrA activation reporter assay could be duplicated using AgrA from *S. epidermidis* for dual screening of compound libraries for inhibitors of *S. aureus* but not *S. epidermidis* AgrA DNA binding function. Using this strategy a drug selective for *agr* inhibition in *S. aureus* could be developed with appropriate bioavailability and pharmacokinetic properties to enhance host defense against skin and soft tissue infections while minimizing the impact on normal microbiota and on antibiotic resistance.

## Materials and Methods

### Ethics statement

All animal experiments were conducted at the AAALAAC accredited Veterinary Medical Unit of the New Mexico Veteran's Affairs Health Care Service in accordance with the applicable portions of the USA Animal Welfare Act as regulated by USDA, the Eighth Edition of *The Guide for the Care and Use of Laboratory Animals*, and the rules and regulations of the USA Department of Veterans Affairs governing experimental vertebrate animal use. These studies were approved by the NMVAHCS Institutional Animal Care and Use Committee (Protocol #10-HG-41). Human neutrophils were purchased from AllCells and the source of the neutrophils was anonymous.

### Reagents

AIPs1-4 were synthesized by Biopeptide Co., Inc and stored in DMSO at −80°C. Savirin (3-(4-propan-2-ylphenyl) sulfonyl-1H-triazolo [1,5-a] quinazolin-5-one, CID#3243271) was synthesized by ChemDiv, confirmed purified by HPLC, and stored in DMSO at −80°C.

### Bacterial strains and growth conditions

The *S. aureus* strains used in this study were as follows: USA 300 strain LAC and its *agr* deletion mutant as described [15], [37]; ALC1743 (*agr* I [*agr::*P3-gfp]) and ALC3253 (Newman [*agr*::P3-gfp]) as described [17], [18]; AH1677 (*agr* I LAC [*agr::*P3-yfp]); AH430 (*agr*II 502a [*agr:*:P3-yfp]), AH1747 (*agr* III MW2 [*agr::*P3-yfp]), AH1872 (*agr* IV MN TG [*agr:*:P3-yfp]) as described [19]; and *agr* IV clinical isolates (NRS165 and NRS166) were obtained through the Network on Antimicrobial Resistance in *Staphylcoccus aureus* (NARSA) supported under NIAID, NIH contract No. HHSN272200700055C. MRSA and MSSA clinical isolates were provided by Dr. Larry Massie, Pathology Service, NMVAHCS and *agr* typed by PCR as described [19]. *Staphylococcus epidermidis* strain #12228 was obtained from ATCC and a *Pseudomonas aeruginosa* isolate was provided by Dr. Graham Timmins, College of Pharmacy, University of New Mexico. To generate early exponential phase, non-quorum sensing bacteria, frozen stocks were cultured in trypticase soy broth (TSB) (Becton Dickinson) as described [17]. CFU were determined after washing in PBS/0.1% Triton X-100 and sonication to disrupt clumps by plating serial dilutions on blood agar plates. Growth in TSB was measured at OD_600_ in 96 well plates using a plate reader (Molecular Devices) at 37°C with shaking, reading at 30 min intervals for 16 hr. The initial cultures were sufficiently diluted such that the maximal OD_600_ was confirmed to be within the linear range of the plate reader (<1.25 OD_600_). Additionally, growth was measured in 5 ml cultures in 50 ml sterile conical tubes with shaking and the OD_600_ determined on 1∶2 and 1∶4 dilutions of the bacterial cultures to ensure that maximal growth was adequately detected and the OD_600_ of the diluted samples was under 0.8 and clearly within the linear range of the spectrophotometer.

### High-throughput screen

A fluorescence-based, high throughput assay was developed to screen 24,087 compounds selected for diversity from the Molecular Libraries Small Molecule Repository of the NIH Molecular Libraries Screening Center Network (summary available at http://pubchem.ncbi.nlm.nih.gov/assay/assay.cgi?aid=1206&loc=ea_ras). Using the Hypercyt flow cytometry sampling platform [49], a 384 well plate format was used that contained per well 2.5×10^7^ early exponential phase ALC1743 containing *agr*::P3 driving expression of GFP. After incubation for 3 hrs with 100 nM synthetic AIP1, the induced fluorescence of the bacteria was compared between vehicle controls and compounds in 0.2% DMSO. Erythrosin B generated singlet oxygen was used as a positive control to inactivate AIP1 [17], [19]. Secondary assays included evaluation with a separate reporter strain ALC3253 in 1 ml assays and analysis of viability at 3 hr by CFU determination.

### *agr:*:P3 promoter activation

Early exponential phase non-fluorescent *agr::* P3 reporter strains (2×10^7^/ml TSB) were incubated (200 rpm at 37°C) in polystyrene tubes with broth, 50 nM synthetic AIP, or indicated concentrations of savirin for the indicated time. After incubation, bacteria were centrifuged (3000 rpm, 4 minutes, 4°C), supernatants decanted, and the pellet washed with PBS/0.1% Triton X-100, fixed with 1% paraformaldehyde containing 25 mM CaCl_2_, sonicated, and then evaluated for fluorescence by flow cytometry (Accuri C6, Accuri Cytometers, Inc., Ann Arbor, MI). Promoter activation was measured as induction of fluorescence.

### Quantitative RT-PCR

Quantitative RT-PCR was carried out for transcripts of interest relative to 16S RNA using a probe-based assay as described with minor modifications [18], [19]. Early exponential phase *S. aureus* strains and clinical isolates were cultured as indicated in the figure legends. For *S. epidermidis*, overnight culture supernatant was used as a source of AIP. It was Millipore filtered and diluted 1∶2 with TSB. RNA was isolated and purified using the Qiagen RNA Protect Bacteria Reagent and RNeasy Mini Kit (Qiagen) using both mechanical and enzymatic disruption. RNA was purified with silica columns and subjected to DNase treatment to remove contaminating DNA. cDNA was generated using a high capacity cDNA RT kit with an RNAse inhibitor (Applied Biosystems) and a Bio-Rad thermocycler. Thermal cycling conditions were as follows: 10 minutes at 25°C, 120 minutes at 37°C, 5 minutes at 85°C, hold at 4°C. Quantitative PCR was performed using an ABI7500 Real-Time PCR system with Taqman Gene Expression master mix, ROX probe/quencher, and appropriate primer sequences (Applied Biosystems). Samples were assayed in triplicate. The data are represented as the fold increase of the transcript relative to 16S compared to the inoculum bacteria. The primer-probe sequences used were as follows: For *S. aureus*: *RNAIII* forward primer AATTAGCAAGTGAGTAACATTTGCTAGT, *RNAIII* reverse primer GATGTTGTTTACGATAGCTTACATGC, RNAIII probe FAM-AGTTAGTTTCCTTGGACTCAGT-GCTATGTATTTTTCTT-BHQ; *psm*α forward primer TAAG-CTTAATCGAACAATTC, *psmα* reverse primer CCCCTTCAAATA-AGATGTTCATATC, *psm*α probe FAM-AAAGACCTCCTTTGTTTGTTA-TGAAATCTTATTTACCAG-BHQ; *hla* forward primer ACAATTTTAGAGAGCCCAACTGAT, *hla* reverse primer TCCCCAATTTTGATTCACCAT, *hla* probe FAM-AAAAAGTAGGCTGGAAAGTGATA-BHQ; *pvl-lukS* forward primer CACAAAATGCCAGTGTTATCCA, *pvl-lukS* reverse primer TTTGCAGCGTTTTGTTTTCG, *pvl-lukS* probe FAM-AGGTAACTTCAATCCAGAATT-TATTGGTGTCCTATC-BHQ-2; *16S* forward primer TGATCCTGGCTCAGGATGA, *16S* reverse primer TTCGCTCGACTTGCATGTA, *16S* probe FAM-CGCTGGCGGCGTGCCTA-BHQ; *agrA* forward primer CTACAAAGTTGCAGCGATGGA, *agrA* reverse primer TGGGCAATGAGTCTGTGAGA, *agrA* probe FAM-AGAAACTGCACATACACGCT-BHQ; *agrC* forward primer AAGATGACATGCCTGGCCTA, *agrC* reverse primer TGTGCACGTAAAATTTTCGCAG, *agrC* probe FAM- TGGTATCGAGAATCTTAAAGTACGTG-BHQ; and *set7* forward primer ACGGAAAAACCAGTTCATGC, *set7* reverse primer GCTTATCTTTGCCAATTAAAGCA, *set7* probe FAM-CAGGTTATATCAGTTTCATTCAACCA-BHQ. For *S. epidermidis*: *16S* forward primer TACACACCGCCCGTCACA, *16S* reverse primer CTTCGACGGCTAGCTCCAAAT, *16S* probe FAM-CACCCGAAGCCGGTGGAGTAACC-BHQ; and RNAIII forward primer ACTAAATCACCGATTGTAGAAATGATATCT, *RNAIII* reverse primer ATTTGCTTAATCTAGTCGAGTGAATGTTA, *RNAIII* probe FAM-ATTTGCTTAATCTAGTCGAGTGAATGTTA-BHQ.

### Membrane integrity

Membrane integrity was measured as described using propidium iodide [25]. LAC was cultured overnight (18 hr) in RPMI supplemented with 1% casamino acids in the presence of savirin (5 µg ml^−1^) or vehicle control. The cultures were washed by centrifugation and the pellet resuspended in PBS supplemented with 1% BSA. The samples were set to an OD_600_ of 0.4 and an aliquot was heat killed (90°C for 10 minutes) to serve as a positive control. Samples (50 µl) were mixed with 1 ml PBS/1% BSA containing propidium iodide. Membrane damage was determined by measuring bacterial fluorescence by flow cytometry (Accuri C6).

### Membrane potential

Membrane potential was measured using the BacLight Membrane Potential Kit (Molecular Probes) following the manufacturer's recommendations. Membrane potential in this assay is based on the shift between the green fluorescence of DiOC_2_ to red in the cytosol of bacteria with higher membrane potential. The proton ionophore CCCP was used as a positive control for disrupting membrane potential. LAC was cultured with 50 nM AIP1 for 5 hr in TSB in the presence of savirin (5 µg ml^−1^) or vehicle control. After diluting into TSB, the bacteria were incubated with 30 µM DiOC_2_ in the dark for 16 min prior to analyzing by flow cytometry (Accuri, C6). Measurements from both the red and green channels were taken and data presented as a ratio of red channel divided by the green channel to reflect the shift to greater change in membrane potential.

### *In silico* docking to AgrA_c_

Savirin (PubChem ID SMR000016143) was docked onto the C-terminal domain of AgrA of *S. aureus* AgrA_c_ (residues 137–238 with an initiator methionine) deposited in the Protein Data Bank (PDB) accession number 3BS1 [50] using the online server SwissDock (http://www.swissdock.ch) [28]. The docking origin was set near Val235 with a search area of 10 Å in all directions and allowing for flexible side chains within 3 Å of the ligand. A model of the *S. epidermidis* AgrA DNA binding domain was prepared by threading the amino acid sequence (UniProt database accession number Q84FX9) onto the structural coordinates of the *S. aureus* protein (PDB 3BS1) using the I-TASSER server (http://zhanglab.ccmb.med.umich.edu/I-TASSER/). Savirin docking to *S. epidermidis* AgrA_c_ was performed as described above for *S. aureus* AgrA_c_ with the origin set to the Cα atom of Phe229. Structural images were generated using PyMOL (PyMOL Molecular Graphics System, v. 1.5.04, Schrödinger, LLC).

### AgrA EMSA

*E. coli* expressing the 6X-histidine tagged C-terminal DNA binding domain of AgrA (AgrA_C_) from *S. aureus* isolate Newman was provided by Dr. Chuan He (University of Chicago, Chicago, IL, USA). Expression and purification of AgrA_C_ was carried out as previously described with minor modifications [29]. Briefly, AgrA_C_ expressing *E. coli* were grown in Terrific broth to an OD_600_ of 0.6 and induced with 1 mM isopropyl β-D-1 thiogalactopyranoside overnight at room temperature. Harvested cells were flash frozen in liquid nitrogen, thawed and lysed using lysozyme and sonication. Soluble AgrA_C_ was affinity purified using Talon Superflow Metal Affinity Resin (Clonetech) followed by gel filtration on a Superdex S200 column (GE Healthcare). Tris (2-carboxyethyl) phosphine (TCEP) at 1 mM was used as a reducing agent throughout purification. Protein was stored at −80°C in PBS, 20% glyercol, 5 mM DTT, 1 mM TCEP and 1 mM MgCl_2_. Electrophoretic mobility shift assays (EMSA) using purified AgrA_C_ (2 µM) were performed as described [27] using a 16 base pair DNA duplex probe (0.1 µM) containing the high affinity LytTR binding site present in both *agr* P2 and P3 [27]. It was synthesized with a 3′ 6-fluorescein (FAM) to facilitate detection (Integrated DNA Technologies, USA). Samples including AgrA_C_, DNA probe, vehicle and/or savirin (5–160 µg ml^−^1 or 13.5–432 µM) were loaded in Tris-acetate-EDTA (TAE) buffer containing 10 mM dithiothreitol. Assays including the 16 bp probe were run with 10% native polyacrylamide gels.

### AgrA activation reporter assay

An AgrA-dependent *lux* reporter strain, AH3048, was generated by transforming *S. aureus* Δ*agr* strain ROJ48 [31] with pCM63 [51]. Plasmid pCM63 consists of the *agrA* gene cloned into plasmid pEPSA5, which placed transcription of *agrA* under the control of the xylose-inducible Tx5 promoter. To construct plasmid pCM63, the *agrA* gene was PCR amplified from AH1263 genomic DNA using primers CML609 (5′-GTTGTTGAATTCCCATAAGGATGTGAATG-3′) and CLM610 (5′-GTTGTTTCTAGACTTATTATATTTTTTTAACGTTTCTCACCG-3′), the PCR product was digested with EcoRI and XbaI, and ligated into similarly digested pEPSA5. Preliminary experiments demonstrated that light production by AH3048 increased in a xylose dose-dependent fashion, without impacting growth, up to a xylose concentration of 0.25%. For testing the impact of savirin on light production, AH3048 cultures were not induced with xylose because the constitutive level of *agrA* transcription from pCM63 was sufficient for luminescence induction. An overnight culture of AH3048 grown in TSB with 10 µg ml^−1^ chloramphenicol (for plasmid maintenance) was used to inoculate (at 1∶500 dilution) TSB containing antibiotic in 96-well microtiter plates (Costar 3603) at 200 µl per well. A 2-fold serial dilution series of savirin (0.4–6.3 µM or 0.29–2.33 µg ml^−1^) was used and the concentrations were tested in quadruplicate. Microtiter plates were incubated at 37°C with shaking (1000 rpm) in a Stuart SI505 incubator (Bibby Scientific, Burlington, NJ) with a humidified chamber. Luminescence and OD_600_ readings were recorded at 30 min increments using a Tecan Systems (San Jose, CA) Infinite M200 plate reader. Maximal light production occurred after 6 hrs of growth. As a specificity control, a 2-fold dilution series (0.5 nM to 1000 nM) of AIP-2 (Anaspec, Fremont, CA) was tested in quadruplicate, as well as 12 control wells containing vehicle (DMSO). As positive controls, two compounds demonstrated by others to inhibit AgrA_c_ in EMSA assays, diflunisal and 4-phenoxyphenol (Sigma) [27], [32], were evaluated for luminescence inhibition in the same assay at concentrations from 1.56–100 µM.

### Microarray analysis

To compare the transcript levels of LAC and the Δ*agr* mutant in the presence or absence of savirin (5 µg ml^−1^), the bacteria were grown for 5 hr in TSB with 50 nM AIP1 or an equivalent amount of DMSO as the vehicle control and processed for microarray analysis as described [33]. The comparisons were LAC vehicle vs. LAC savirin, Δ*agr* vehicle vs. Δ*agr* savirin, and LAC vehicle vs. Δ*agr* vehicle, n = 3. The bacterial RNA was purified as described [32]. Samples were hybridized to a custom Affymetrix GeneChip (RMLchip7) that contains all open reading frames of the USA300 genome. Samples were scanned using Affymetrix 7Gplus GeneChip scanner according to standard GeneChip protocols with the image files converted using GeneChip Operating Software (GCOS v1.4). The data were quantile-normalized and a 3-way ANOVA with multiple test correction using the false discovery rate (p<0.05) was performed using Partek Genomics Suite software (Partek, inc. v6.5). These data were combined with fold change values, signal confidence (above background), and call consistency (as a percent) as calculated using custom Excel templates to generate final gene lists for each comparison. The microarray data were deposited in the Gene Expression Omnibus (GEO) database (http://www.ncbi.nlm.nih.gov/projects/geo/) under the accession number GSE52978. All microarray data are MIAME compliant.

### Alpha hemolysin

Alpha hemolysin activity was measured in 0.45 µm filtered cultured supernatant standardized by OD_600_ after bacterial strains were grown overnight in TSB in the presence or absence of savirin (5 µg ml^−1^). The assay was performed using rabbit erythrocyte lysis as described [18]. One unit of hemolytic activity was defined as the amount of bacterial supernatant able to liberate half of the total hemoglobin from the erythrocytes and expressed as HA_50_.

### Neutrophil lysis

The ability of secreted toxins to lyse human neutrophils was determined by LDH release. Overnight supernatant from MRSA *agr* group I clinical isolate #32 generated with either savirin (5 ug ml^−1^) or DMSO vehicle control was 0.45 µm filtered, stored at −80°C, and thawed on ice. Human neutrophils (AllCells) were washed twice in saline to remove EDTA, suspended in RPMI with 10 mM HEPES and 1% HSA, and assessed for viability by Trypan blue staining (>97%). The experiment was run in triplicate and each tube contained 3×10^6^ neutrophils in 100 µl RPMI to which was added 100 µl of either RPMI, TSB diluted 1∶5 or 1∶10 in RPMI, or treated supernatants diluted 1∶5 or 1∶10 in RPMI. PBS with 0.1% Triton-X100 (100 µl) was used for 100% lysis. Tubes were incubated at 37°C in a 5% CO2 incubator for 1 and 2 hours. At each time, the tubes were centrifuged at 13,000 rpm, at 4°C, for 5 minutes. Cell free supernatant (100 µl) was transferred to a micotiter plate and immediately processed for LDH according to the Cytotoxicity Detection Kit (Roche). A blank was created for each plate with 10%TSB in RPMI. The data are depicted as the percentage of total lysis after correction for LDH release stimulated by media alone.

### Mouse infection models

For all *in vivo* experiments, savirin was solubilized at 1 mg ml^−1^ in 0.5% hydroxypropyl methylcellulose (Sigma) in endotoxin-free sterile water made 3.0 mM in NaOH with cell culture tested 1 N NaOH (Sigma), and put through a 0.22 µM filter (Millex-GV). The vehicle control was the HPMC used to solubilize the savirin. Sample sizes were determined by preliminary experiments to determine the number of mice required to observe significance. *Dermonecrosis model*: SKH1 hairless immunocompetent mice (≈8–16 wk, ≈26–34 g, male) were obtained from Charles Rivers (Wilmington, MA). At Day 0, early exponential phase bacteria (4×10^7^) washed in sterile normal saline were injected concurrently with savirin (5 µg) or vehicle in 50 µl subcutaneously into the flank using a 3/10 cc insulin syringe with a 28 ½ gauge needle (Becton Dickinson). For delayed delivery, 10 µg savirin was administered 24 and 48 hr after infection in 50 µl. The animals were divided into two groups to have equivalent mean abscess sizes prior to administering drug or vehicle. Abscess area (maximal on Day 1) and ulcer area (necrosis optimal on Days 3–4) were measured with calipers as described [15], [38] and recorded daily in addition to weight loss. The slightly raised abscess area (mm^2^) was calculated from the equation (π/2)[(length of the abscess)×(width of the abscess)]. The flat ulcer area (mm^2^) was calculated from the equation (length of the ulcer)×(width of the ulcer) or alternatively from digital images using Adobe Photoshop standardized to a micrometer with equivalent results. On Day 3 or Day 7, the mice were euthanized using isoflurane inhalation. The abscess/ulcer area was excised (1.5 cm^2^) and the spleens removed. Tissues and spleens were placed in 1 ml of HBSS/0.1% HSA in a bead-beating tube containing sterile 2.3 mm beads (Biospec) and were processed for bacterial CFU by homogenizing the spleens in a bead beater, diluting all samples 1∶10 in 1 ml PBS/0.1%Triton, sonicating, and plating serial dilutions on blood agar as described [17]–[19]. *Airpouch model*: age matched *Nox2^−/−^* male mice (Jackson Labs) or C57BL/6 male mice (Charles Rivers) were infected with either 2×10^7^ early exponential phase non-fluorescent AH1677 bacteria (*Nox2^−/−^*) or 5×10^7^ LAC Δ*agr* (C57BL/6) into an air pouch generated by the injection of 5 ml of air subcutaneously as described [17]–[19]. Savirin (10 µg) vs. vehicle in 50 µl was injected into the pouch at time 0. After 24 hours, weight loss was determined, the air pouch was lavaged with HBSS/0.1% HSA and the kidneys removed. The bacteria in the lavage were analyzed by flow cytometry for promoter activation (AH1677) and both the lavage and kidneys processed as above for CFU determination.

### Macrophage intracellular killing

Early exponential phase LAC+50 nM synthetic AIP1 or Δ*agr* LAC (2×10^7^/ml TSB) were incubated for 5 hr at 37°C with shaking (200 rpm) in the presence of savirin (5 µg ml−1) or vehicle control. Bacteria were opsonized (1×10^8^/ml) with rabbit IgG anti-*Staphylcoccus aureus* (Accurate Antibody YVS6881) (100 µg/ml) in phenol red-free Dulbecco's Modified Eagle Media, DMEM, containing 4.5 g/L D-glucose/2%Hepes+1% FCS). The experiment was performed in triplicate. Murine macrophage RAW264 cells (5×10^6^) in 250 µl of DMEM+2% FCS were combined with 5×10^6^ opsonized bacteria in 250 µl of DMEM+1% FCS (MOI 1∶1) in sterile polystyrene 12×75 mm tubes, centrifuged briefly to initiate contact, and incubated for 1 hr at 37°C in 5% CO_2_. The infected cells were treated with lysostaphin (Sigma) (2 µg/ml for 15 min) to kill extracellular bacteria and then washed and suspended in fresh media. Half of the samples were incubated for an additional 4 hrs. To determine the intracellular CFU at 1 and 5 hr, the relevant cells were centrifuged, suspended in PBS/0.1% Triton-X-100 and sonicated to disrupt cells and dilutions plated on blood agar. The cell line was tested for *Mycoplasma sp*. contamination by PCR (Life Technologies).

### Low pH and linoleic acid killing

Early exponential phase LAC+50 nM synthetic AIP1 or Δ*agr* LAC (1×10^8^/ml DMEM, 4.5 g/L D-glucose/2%Hepes) were incubated for 5 hr at 37°C with shaking (200 rpm) in the presence of savirin (5 µg ml^−1^) or vehicle control. Bacteria were centrifuged, washed, resuspended in DMEM/2%Hepes acidified with either HCl to pH 2.5 or 10 µg ml^−1^ linoleic acid (Sigma) and incubated for the indicated times. Dilutions were plated on sheep blood agar to determine the residual viability.

### Resistance

#### *In vivo* passage

The induction of resistance to clindamycin in LAC served as a positive control and is based on the presence of ermC (SAUSA300 pUSA 030007) which confers erythromycin and clindamycin resistance [44]. Early exponential phase bacteria (4×10^7^) were injected simultaneously with erythromycin (Sigma) (16 µg) and clindamycin (Sigma) (0.12 µg) or savirin (5 µg) into the flank of a SKH1 mouse as described above. At 24 hr, weight loss was recorded and the mouse was euthanized using isoflurane inhalation. The abscess area was measured, excised, and placed in 1 ml of HBSS/0.1% HSA in a bead-beating tube and processed as described above. Serial dilutions of the bacteria from the abscess were plated on blood agar plates and were incubated at 37°C for 24 hr. Colonies were lifted from the blood agar plates and sonicated in saline until a desired OD_600_ was reached. These bacteria were injected simultaneously with erythromycin (16 µg) and clindamycin (0.12 µg) or savirin (5 µg) into the flank of the next SKH1 mouse. The procedure was repeated through ten mice total for each treatment. After the tenth mouse in each group was euthanized, the bacteria taken from the plate were concentrated and stored in TSB/10% glycerol at −80°C for subsequent analysis.

#### *In vitro* passage

early exponential phase LAC (1×10^6^ ml^−1^) were incubated in 5 ml TSB with the addition of erythromycin (16 µg ml^−1^) and clindamycin (0.12 µg ml^−1^) or savirin (5 µg ml^−1^) at 37°C with shaking (200 rpm). After 24 hr, the bacteria were washed by centrifugation and diluted into 5 ml fresh TSB and new addition of drug or savirin was added to the respective tubes for a total of ten days. The passaged bacteria exposed to erythromycin and clindamycin either in vivo or in vitro and non-passaged bacteria (1×10^6^ in 5 ml TSB) were incubated overnight (18 hr) with erythromycin (16 µg ml^−1^) and increasing doses of clindamycin (0.06–1.0 µg ml^−1^) and plated to determine viability. Either in vivo or in vitro passaged bacteria exposed to savirin and non-passaged bacteria (2×10^7^ ml^−1^ TSB) were evaluated for the ability to respond to savirin by incubation in 1 ml cultures with 50 nM synthetic AIP1 in the presence of additional savirin (1–5 µg ml^−1^) for 2 hr at 37°C with shaking (200 rpm). Expression of RNAIII was determined by qRT-PCR as described above.

#### Evaluation of *in vivo* passaged bacteria on milk agar plates

To evaluate resistance at the colony level, agar plates were made with 2.5% skim milk containing either 10 µg ml^−1^ savirin in DMSO or an equivalent amount of DMSO as a vehicle control to determine savirin's ability to inhibit *agr*-dependent protease production. The in vivo passaged bacteria were washed, suspended in TSB, standardized by OD_600_, diluted with TSB to give approximately 15–20 colonies per plate, and spread onto the milk agar plates. After incubation for 72 hr at 37°C, colonies were counted and evaluated for proteolysis by measuring the clear zone surrounding the colony with a micrometer. Colonies with zones ≥1 mm were counted as proteolytic. Non-passaged Δ*agr* LAC were plated for comparison.

### Statistical evaluation

*In vitro* data were analyzed by the two-tailed Student's *t*-test or two way measures ANOVA as indicated in figure legends. *In vivo* data were analyzed by the two-tailed Mann-Whitney U test for non-parametrics. All evaluations were conducted using GraphPad Prism v. 5.o and results were considered significantly different with *p*<0.05.

## Supporting Information

Figure S1 Effect of savirin on *agr::* P3 promoter activation in strain Newman (ALC3243) (**A**) at 3 hr of incubation compared to (**B**) log CFU at 3 hr from a starting CFU of 2×10^7^/ml. Mean ± SEM, n = 5. ***p<0.001 by two-tailed Student's *t*-test.
(TIF)

Figure S2 Savirin inhibits *agr*::P3 promoter activation in all 4 *agr* alleles. Effect of 5 µg ml^−1^ savirin vs vehicle on *agr*::P3 promoter activation in an *agr* I strain (AH1677), an *agr* II strain (AH430), an *agr* III strain (AH1747), and an *agr* IV strain (AH1872) after incubation for 14 hr. Data are represented as mean fluorescence units of total *S. aureus* ± SEM, n = 3. *** p<0.001 by two-tailed Student's *t*-test.
(TIF)

Figure S3 Effect of savirin on growth of *S. aureus* and *S. epidermidis* in bulk cultures. (A) *S. aureus* and (B) *S. epidermidis* were incubated with savirin (5 µg ml^−1^) (red line) vs. vehicle (blue line) in 5 ml cultures with shaking for the indicated times. The cultures were diluted 1∶2 and 1∶4 before reading at OD_600_ to ensure that the readings were within the linear range of the spectrophotometer. Mean ± SEM, n = triplicates of a representative experiment. ***p<0.001 **p<0.01, *p<0.05 by two-tailed Student's *t*-test.
(TIF)

Figure S4 Savirin does not affect membrane potential or membrane integrity. (**A**) Membrane integrity measured as propidium iodide uptake by LAC *agr*+ cultured overnight with savirin (5 µg ml^−1^) vs. vehicle control. Heat killed LAC was a positive control for the assay. Mean ± SEM, n = 3. (**B**) Membrane potential measured as a shift in fluorescence (DiOC2) of LAC plus 50 nM AIP1 cultured for 5 hr with savirin (5 µg ml^−1^) vs. vehicle control. CCCP-treated LAC was a positive control for the assay and demonstrated collapse of membrane potential. Mean ± SEM., n = 3. ***p<0.001 **p<0.01, *p<0.05 by two-tailed Student's *t*-test.
(TIF)

Figure S5 Effect of diflunisal and 4-phenoxyphenol on AgrA promoter activation. Effect of increasing concentrations of diflunisal and 4-phenoxyphenol (1.56–100 µM) vs vehicle on *agrA* reporter activation in an *agr* null strain expressing a plasmid for *agrA* where *agr*::P3 drives luminescence, AH3048, after 6 hr of growth. AIP2 as an inhibitor of non-*agr*II AgrC signaling was used as a specificity control. Viability is represented as OD_600_. Data are represented as the mean ± SEM of quadruplicates of a representative experiment.
(TIF)

Figure S6 Savirin inhibits *agr*-dependent virulence factor production in clinical isolates. Effect of savirin (5 µg ml^−1^) vs vehicle on overnight alpha-hemolysin production (measured as HA_50_) by current clinical isolates that represent MRSA and MSSA from multiple different sites of infection and of *agr* alleles I–III.
(TIF)

Figure S7 Comparison of USA300 LAC *agr* ^+^ vs Δ*agr* LAC for clearance from the skin. Hairless SKH1 mice were infected with 4×10^7^ bacteria and the abscess CFU determined at the indicated times. Data are represented as mean ± SEM, n = 4 mice per group. *p<0.05, **p<0.01 by two-tailed Mann-Whitney U test.
(TIF)

Table S1 Changes in the USA300 LAC transcriptome by microarray.
(DOCX)

Table S2 Transcripts upregulated by savirin in LAC *agr*+ and *Δagr*.
(DOC)
