# Supplementary figures and images for: Selective Chemical Inhibition of agr Quorum Sensing in Staphylococcus aureus Promotes Host Defense with Minimal Impact on Resistance
Source: PLoS Pathog. 2014 Jun 12;10(6):e1004174. doi: 10.1371/journal.ppat.1004174 (PMC4055767; doi:10.1371/journal.ppat.1004174)

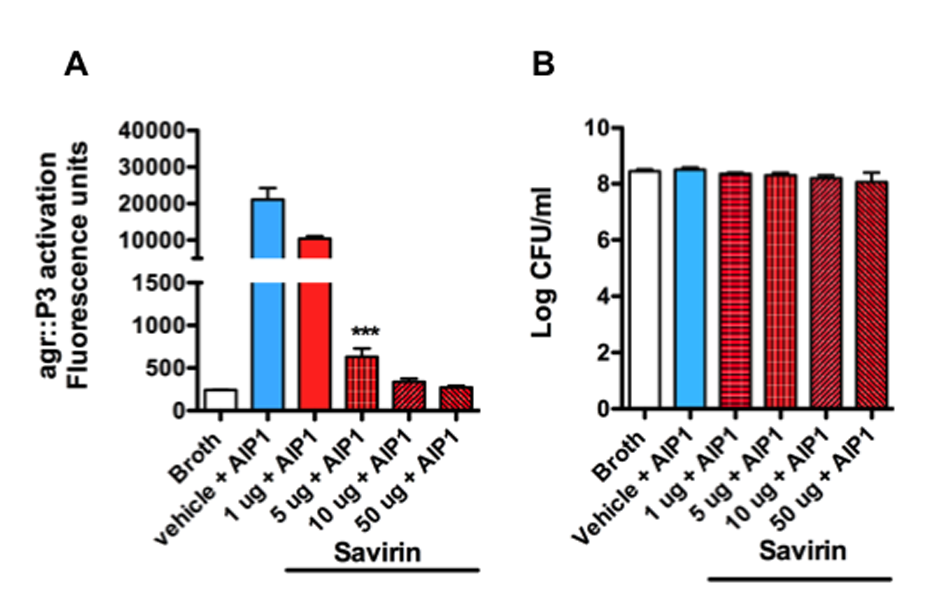

Supplement: Figure S1 — Effect of savirin on agr:: P3 promoter activation in strain Newman (ALC3243) (A) at 3 hr of incubation compared to (B) log CFU at 3 hr from a starting CFU of 2×10^7^/ml. Mean ± SEM, n = 5. ***p<0.001 by two-tailed Student's t-test. (TIF) [file ppat.1004174.s001.tif]

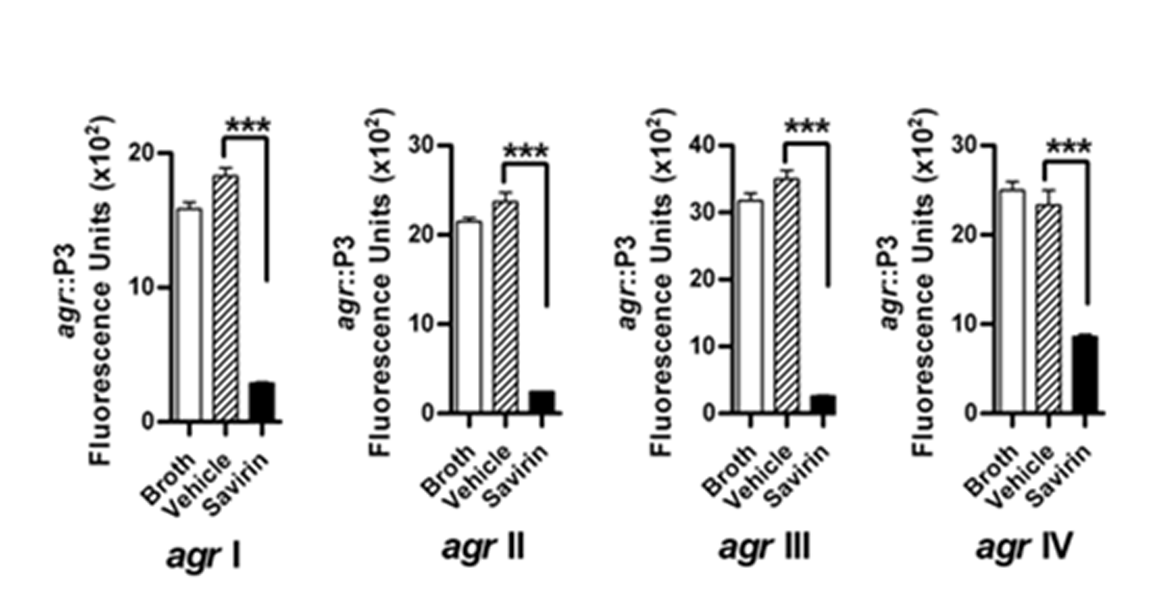

Supplement: Figure S2 — Savirin inhibits agr::P3 promoter activation in all 4 agr alleles. Effect of 5 µg ml−1 savirin vs vehicle on agr::P3 promoter activation in an agr I strain (AH1677), an agr II strain (AH430), an agr III strain (AH1747), and an agr IV strain (AH1872) after incubation for 14 hr. Data are represented as mean fluorescence units of total S. aureus ± SEM, n = 3. *** p<0.001 by two-tailed Student's t-test. (TIF) [file ppat.1004174.s002.tif]

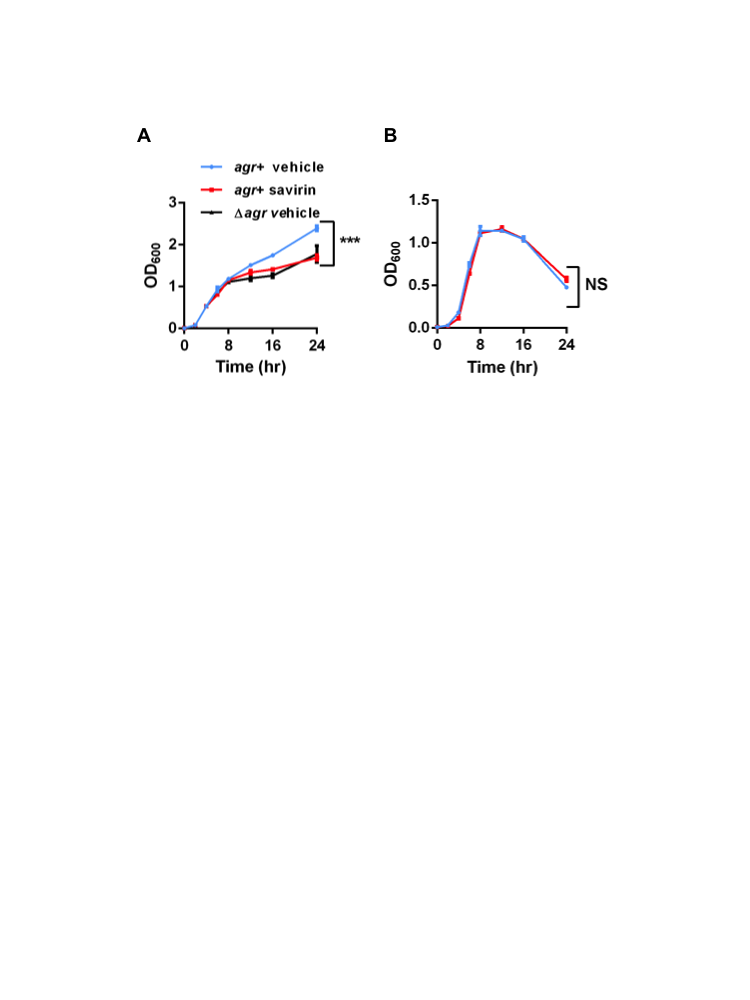

Supplement: Figure S3 — Effect of savirin on growth of S. aureus and S. epidermidis in bulk cultures. (A) S. aureus and (B) S. epidermidis were incubated with savirin (5 µg ml−1) (red line) vs. vehicle (blue line) in 5 ml cultures with shaking for the indicated times. The cultures were diluted 1∶2 and 1∶4 before reading at OD600 to ensure that the readings were within the linear range of the spectrophotometer. Mean ± SEM, n = triplicates of a representative experiment. ***p<0.001 **p<0.01, *p<0.05 by two-tailed Student's t-test. (TIF) [file ppat.1004174.s003.tif]

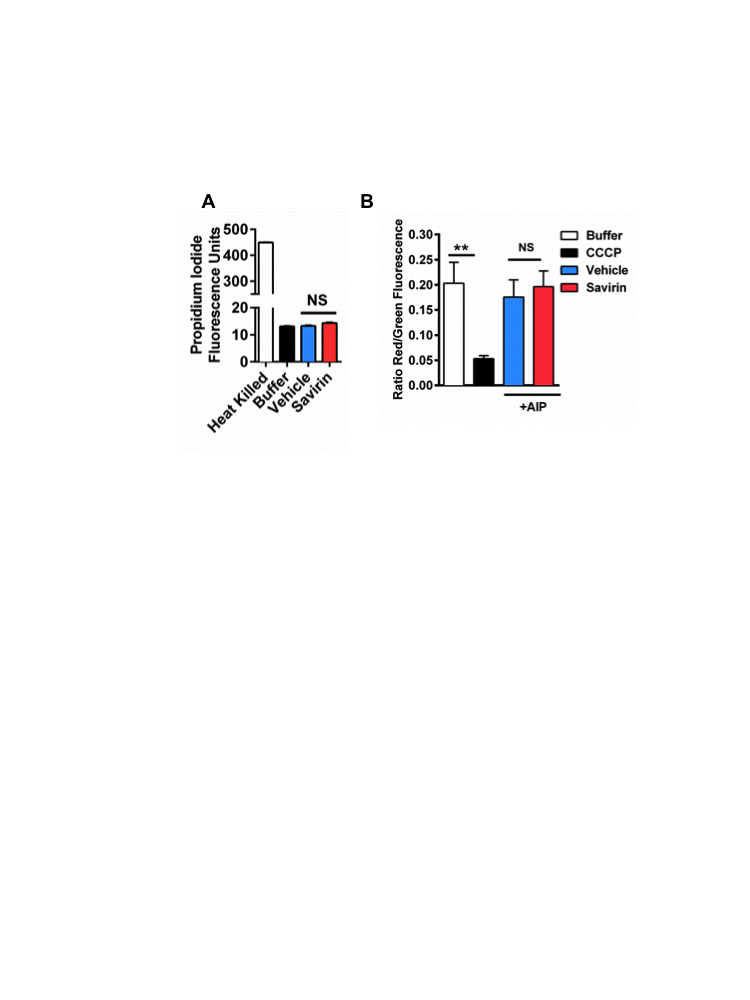

Supplement: Figure S4 — Savirin does not affect membrane potential or membrane integrity. (A) Membrane integrity measured as propidium iodide uptake by LAC agr+ cultured overnight with savirin (5 µg ml−1) vs. vehicle control. Heat killed LAC was a positive control for the assay. Mean ± SEM, n = 3. (B) Membrane potential measured as a shift in fluorescence (DiOC2) of LAC plus 50 nM AIP1 cultured for 5 hr with savirin (5 µg ml−1) vs. vehicle control. CCCP-treated LAC was a positive control for the assay and demonstrated collapse of membrane potential. Mean ± SEM., n = 3. ***p<0.001 **p<0.01, *p<0.05 by two-tailed Student's t-test. (TIF) [file ppat.1004174.s004.tif]

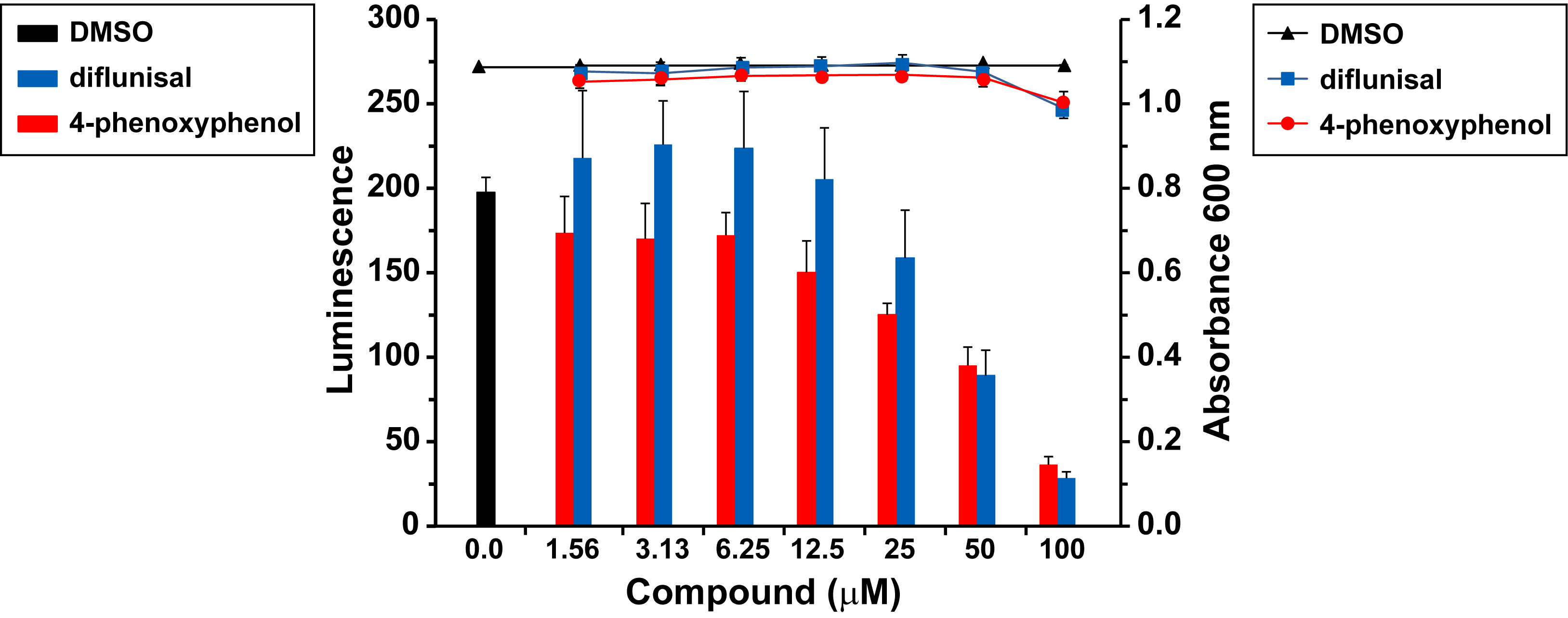

Supplement: Figure S5 — Effect of diflunisal and 4-phenoxyphenol on AgrA promoter activation. Effect of increasing concentrations of diflunisal and 4-phenoxyphenol (1.56–100 µM) vs vehicle on agrA reporter activation in an agr null strain expressing a plasmid for agrA where agr::P3 drives luminescence, AH3048, after 6 hr of growth. AIP2 as an inhibitor of non-agrII AgrC signaling was used as a specificity control. Viability is represented as OD600. Data are represented as the mean ± SEM of quadruplicates of a representative experiment. (TIF) [file ppat.1004174.s005.tif]

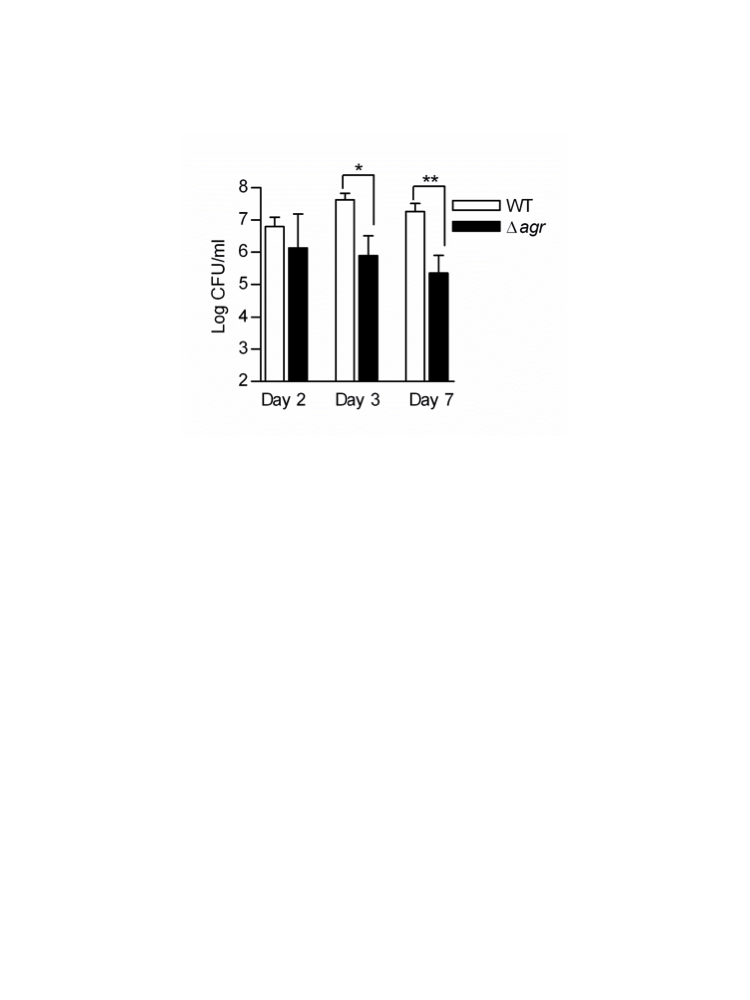

Supplement: Figure S7 — Comparison of USA300 LAC agr + vs Δagr LAC for clearance from the skin. Hairless SKH1 mice were infected with 4×10^7^ bacteria and the abscess CFU determined at the indicated times. Data are represented as mean ± SEM, n = 4 mice per group. *p<0.05, **p<0.01 by two-tailed Mann-Whitney U test. (TIF) [file ppat.1004174.s007.tif]
