# Supplementary material for: Selective Chemical Inhibition of agr Quorum Sensing in Staphylococcus aureus Promotes Host Defense with Minimal Impact on Resistance
Source: PLoS Pathog. 2014 Jun 12;10(6):e1004174. doi: 10.1371/journal.ppat.1004174 (PMC4055767; doi:10.1371/journal.ppat.1004174)
Supplement: Table S1 — Changes in the USA300 LAC transcriptome by microarray. (DOCX) [file ppat.1004174.s008.docx]

**Table S1.** Changes in the USA300 LAC transcriptome by microarray

| **ORF** | **Gene** | **Description/function** |  |  |  |
| --- | --- | --- | --- | --- | --- |
|  |  |  | **LAC WT**  **vehicle vs. savirin** | **Δ*agr***  **vehicle vs Savirin** | **LAC WT vs*.Δagr*** |
| SAUSA300_0001 | *dnaA* | Chromosomal replication initiator protein dnaA |  |  |  |
| SAUSA300_0002 | *dnaN* | DNA polymerase III, beta chain (EC 2.7.7.7) |  |  |  |
| SAUSA300_0003 | *-* | Hypothetical cytosolic protein |  |  |  |
| SAUSA300_0004 | *recF* | DNA replication and repair protein recF |  |  |  |
| SAUSA300_0005 | *gyrB* | DNA gyrase subunit B (EC 5.99.1.3) |  |  |  |
| SAUSA300_0006 | *gyrA* | DNA gyrase subunit A (EC 5.99.1.3) |  |  |  |
| SAUSA300_0007 | *-* | Sugar kinase | 2.18 |  | 2.27 |
| SAUSA300_0008 | *hutH* | Histidine ammonia-lyase (EC 4.3.1.3) |  | 4.89 | -2.11 |
| SAUSA300_0009 | *serS* | Seryl-tRNA synthetase (EC 6.1.1.11) |  |  |  |
| SAUSA300_0010 | *-* | Branched-chain amino acid transport protein AzlC |  |  |  |
| SAUSA300_0011 | *-* | Branched-chain amino acid transport protein AzlD |  |  |  |
| SAUSA300_0012 | *-* | Homoserine O-acetyltransferase (EC 2.3.1.31) |  |  |  |
| SAUSA300_0013 | *-* | Hypothetical protein |  |  |  |
| SAUSA300_0014 | *-* | Phosphoesterase, DHH family protein |  |  |  |
| SAUSA300_0015 | *rplI* | LSU ribosomal protein L9P |  |  |  |
| SAUSA300_0016 | *dnaB* | Replicative DNA helicase (EC 3.6.1.-) |  |  |  |
| SAUSA300_0017 | *purA* | Adenylosuccinate synthetase (EC 6.3.4.4) |  |  |  |
| SAUSA300_0020 | *-* | Two-component response regulator yycF |  |  |  |
| SAUSA300_0021 | *-* | Two-component sensor kinase yycG (EC 2.7.3.-) |  |  |  |
| SAUSA300_0022 | *-* | Hypothetical membrane protein yycH |  |  |  |
| SAUSA300_0023 | *-* | Hypothetical protein |  |  |  |
| SAUSA300_0024 | *-* | Hypothetical protein |  |  |  |
| SAUSA300_0025 | *-* | 5'-nucleotidase (EC 3.1.3.5) |  |  |  |
| SAUSA300_0026 | *-* | Hypothetical protein |  |  |  |
| SAUSA300_0027 | *-* | Hypothetical protein |  |  |  |
| SAUSA300_0028 | *-* | Transposase |  |  |  |
| SAUSA300_0029 | *-* | Hydroxymethylglutaryl-CoA synthase (EC 2.3.3.10) |  |  |  |
| SAUSA300_0030 | *-* | Glycerophosphoryl diester phosphodiesterase (EC 3.1.4.46) |  |  |  |
| SAUSA300_0031 | *-* | (R)-specific enoyl-CoA hydratase (EC 4.2.1.-) |  |  |  |
| SAUSA300_0032 | *mecA* | MecA protein |  |  | -2.67 |
| SAUSA300_0033 | *-* | Methicillin resistance mecR1 protein |  |  |  |
| SAUSA300_0034 | *-* | Transposase |  |  |  |
| SAUSA300_0035 | *-* | Type I restriction-modification system restriction subunit (EC 3.1.21.3) |  |  |  |
| SAUSA300_0036 | *-* | Hypothetical protein |  |  |  |
| SAUSA300_0037 | *ccrB* | Site-specific recombinase |  |  |  |
| SAUSA300_0038 | *ccrA* | Site-specific recombinase |  |  |  |
| SAUSA300_0039 | *-* | Hypothetical protein |  |  |  |
| SAUSA300_0040 | *-* | Hypothetical protein |  |  |  |
| SAUSA300_0041 | *-* | Hypothetical protein |  |  |  |
| SAUSA300_0042 | *-* | Transcriptional regulator |  |  |  |
| SAUSA300_0043 | *-* | Hypothetical protein |  |  |  |
| SAUSA300_0044 | *-* | Hydroxyacylglutathione hydrolase (EC 3.1.2.6) |  |  |  |
| SAUSA300_0045 | *-* | Hypothetical protein |  |  |  |
| SAUSA300_0046 | *-* | Hypothetical protein |  |  |  |
| SAUSA300_0047 | *-* | Type II restriction-modification system restriction subunit (EC 3.1.21.4) |  |  |  |
| SAUSA300_0048 | *-* | Hypothetical protein |  |  |  |
| SAUSA300_0049 | *-* | Hypothetical protein |  |  |  |
| SAUSA300_0050 | *-* | Hypothetical membrane spanning protein |  |  |  |
| SAUSA300_0052 | *-* | Type I restriction-modification system specificity subunit |  |  |  |
| SAUSA300_0053 | *speG* | Spermidine N1-acetyltransferase (EC 2.3.1.57) |  |  |  |
| SAUSA300_0054 | *-* | Hypothetical protein |  |  |  |
| SAUSA300_0055 | *-* | Alcohol dehydrogenase (EC 1.1.1.1) |  |  |  |
| SAUSA300_0056 | *-* | Hypothetical protein |  |  |  |
| SAUSA300_0057 | *-* | Pyridoxal phosphate-dependent enzymes |  |  |  |
| SAUSA300_0058 | *-* | Hypothetical protein |  |  |  |
| SAUSA300_0059 | *-* | Hypothetical protein |  |  |  |
| SAUSA300_0060 | *-* | Transposase |  |  |  |
| SAUSA300_0061 | *arcC* | Carbamate kinase (EC 2.7.2.2) |  |  |  |
| SAUSA300_0062 | *arcB* | Ornithine carbamoyltransferase (EC 2.1.3.3) |  |  |  |
| SAUSA300_0063 | *-* | Transcription regulator, crp family |  |  |  |
| SAUSA300_0064 | *arcD* | Arginine/ornithine antiporter |  |  |  |
| SAUSA300_0065 | *arcA* | Arginine deiminase (EC 3.5.3.6) |  |  |  |
| SAUSA300_0066 | *argR* | Arginine repressor, ArgR |  |  |  |
| SAUSA300_0067 | *-* | Putative transcription factors |  |  |  |
| SAUSA300_0068 | *-* | Lead, cadmium, zinc and mercury transporting ATPase (EC 3.6.3.-) |  |  |  |
| SAUSA300_0070 | *-* | Lysophospholipase L2 (EC 3.1.1.5) |  |  |  |
| SAUSA300_0071 | *-* | Transposase |  |  |  |
| SAUSA300_0072 | *-* | Hypothetical protein |  |  |  |
| SAUSA300_0073 | *-* | Nickel-binding protein |  |  |  |
| SAUSA300_0074 | *opp-3B* | Hypothetical protein |  |  |  |
| SAUSA300_0075 | *opp-3C* | Dipeptide transport system permease protein DppC |  |  |  |
| SAUSA300_0076 | *-* | Hypothetical protein |  |  |  |
| SAUSA300_0077 | *-* | Nickel transport ATP-binding protein NikE |  |  |  |
| SAUSA300_0078 | *copA* | Copper-silver efflux ATPase (EC 3.6.3.-) |  |  |  |
| SAUSA300_0079 | *-* | Hypothetical exported protein |  |  |  |
| SAUSA300_0080 | *-* | Hypothetical protein |  |  |  |
| SAUSA300_0081 | *-* | Hypothetical protein |  |  |  |
| SAUSA300_0082 | *-* | Nitrogen regulation protein NIFR3 |  |  |  |
| SAUSA300_0083 | *-* | Hypothetical membrane spanning protein |  |  |  |
| SAUSA300_0084 | *-* | Hypothetical cytosolic protein |  |  |  |
| SAUSA300_0085 | *-* | Rhodanese-related sulfurtransferases / Hypothetical protein |  |  |  |
| SAUSA300_0086 | *-* | Hydroxyacylglutathione hydrolase (EC 3.1.2.6) |  |  |  |
| SAUSA300_0088 | *-* | Hypothetical protein |  |  |  |
| SAUSA300_0089 | *-* | Nitrogen regulation protein NIFR3 |  |  |  |
| SAUSA300_0090 | *-* | Hypothetical cytosolic protein |  |  |  |
| SAUSA300_0091 | *-* | Macrolide-efflux protein |  |  |  |
| SAUSA300_0092 | *-* | Trp repressor binding protein |  |  |  |
| SAUSA300_0093 | *-* | Transcriptional regulators, LysR family |  |  |  |
| SAUSA300_0094 | *-* | Hypothetical protein |  |  |  |
| SAUSA300_0095 | *-* | Transcriptional regulators, LysR family |  |  |  |
| SAUSA300_0096 | *-* | Hypothetical membrane spanning protein |  |  |  |
| SAUSA300_0097 | *-* | Superfamily I DNA helicase |  |  |  |
| SAUSA300_0098 | *-* | Hypothetical protein |  |  |  |
| SAUSA300_0099 | *plc* | 1-phosphatidylinositol phosphodiesterase precursor (EC 4.6.1.13) |  |  |  |
| SAUSA300_0100 | *-* | membrane lipoprotein |  |  |  |
| SAUSA300_0101 | *-* | membrane lipoprotein |  |  |  |
| SAUSA300_0102 | *-* | membrane lipoprotein |  |  |  |
| SAUSA300_0103 | *-* | membrane lipoprotein |  |  |  |
| SAUSA300_0104 | *-* | Transcriptional regulator, AraC family |  |  |  |
| SAUSA300_0105 | *-* | Carboxypeptidase, M20(D) family |  |  |  |
| SAUSA300_0106 | *-* | Multidrug resistance protein B |  |  |  |
| SAUSA300_0107 | *-* | Sodium-dependent phosphate transporter |  |  |  |
| \SAUSA300_0108 | *sok* | Myosin-crossreactive antigen | 3.59 |  |  |
| SAUSA300_0109 | *-* | Transporter, drug/metabolite exporter family |  |  |  |
| SAUSA300_0110 | *-* | Transcriptional regulator, GntR family / tyrosine aminotransferase (ec 2.6.1.5) |  |  |  |
| SAUSA300_0111 | *-* | Hypothetical protein |  |  |  |
| SAUSA300_0112 | *lctP* | L-lactate permease |  |  |  |
| SAUSA300_0113 | *spa* | Immunoglobulin G binding protein A precursor | 7.80 |  | 6.94 |
| SAUSA300_0114 | *-* | Staphylococcal accessory regulator A |  |  | 2.78 |
| SAUSA300_0115 | *sirC* | Staphylobactin transport system permease protein |  |  |  |
| SAUSA300_0116 | *sirB* | Staphylobactin transport system permease protein |  |  |  |
| SAUSA300_0117 | *sirA* | Staphylobactin-binding protein |  |  |  |
| SAUSA300_0118 | *-* | Cysteine synthase (EC 2.5.1.47) |  |  |  |
| SAUSA300_0119 | *-* | Ornithine cyclodeaminase family protein |  |  |  |
| SAUSA300_0120 | *sbnC* | Siderophore biosynthesis IucC protein (EC 6.-.-.-) | 2.82 |  |  |
| SAUSA300_0121 | *-* | Multidrug resistance efflux pump |  |  |  |
| SAUSA300_0122 | *sbnE* | IucA/IucC family siderophore biosynthesis protein |  |  |  |
| SAUSA300_0123 | *sbnF* | IucC family siderophore biosynthesis protein |  |  |  |
| SAUSA300_0124 | *-* | 4-hydroxy-2-oxovalerate aldolase (EC 4.1.2.-) |  |  | 2.61 |
| SAUSA300_0125 | *-* | Diaminopimelate decarboxylase (EC 4.1.1.20) |  |  |  |
| SAUSA300_0126 | *-* | Siderphore biosynthesis protein SbnI |  |  |  |
| SAUSA300_0127 | *-* | Hypothetical protein | -2.30 |  | -3.41 |
| SAUSA300_0128 | *-* | Hypothetical protein |  |  |  |
| SAUSA300_0129 | *-* | (R,R)-butanediol dehydrogenase (EC 1.1.1.4) / Acetoin dehydrogenase (EC 1.1.1.5) |  |  |  |
| SAUSA300_0130 | *-* | Hypothetical protein |  |  |  |
| SAUSA300_0131 | *-* | Undecaprenyl-phosphate galactosephosphotransferase (EC 2.7.8.6) |  |  |  |
| SAUSA300_0132 | *-* | beta-D-Glcp alpha-1,6-galactosyltransferase (EC 2.4.1.-) |  |  |  |
| SAUSA300_0133 | *-* | Secreted polysaccharide polymerase |  |  |  |
| SAUSA300_0134 | *-* | Transcriptional activator amrA |  |  |  |
| SAUSA300_0135 | *-* | Superoxide dismutase (EC 1.15.1.1) |  |  |  |
| SAUSA300_0136 | *-* | Hypothetical protein | 3.59 |  | 3.30 |
| SAUSA300_0137 | *-* | Transcriptional regulator, GntR family |  |  |  |
| SAUSA300_0138 | *deoD* | Purine nucleoside phosphorylase (EC 2.4.2.1) |  |  |  |
| SAUSA300_0139 | *-* | Permease |  |  |  |
| SAUSA300_0140 | *deoC* | Deoxyribose-phosphate aldolase (EC 4.1.2.4) |  |  |  |
| SAUSA300_0141 | *deoB* | Phosphopentomutase (EC 5.4.2.7) |  |  |  |
| SAUSA300_0142 | *phnE* | Phosphonates transport system permease protein PhnE |  |  |  |
| SAUSA300_0143 | *phnE* | Phosphonates transport system permease protein phnE |  |  |  |
| SAUSA300_0144 | *phnC* | Phosphonates transport ATP-binding protein phnC |  |  |  |
| SAUSA300_0145 | *-* | Phosphonates-binding protein |  |  |  |
| SAUSA300_0146 | *-* | Hypothetical protein |  |  |  |
| SAUSA300_0147 | *-* | 2',3'-cyclic-nucleotide 2'-phosphodiesterase (EC 3.1.4.16) / 3'-nucleotidase (EC 3.1.3.6) |  |  |  |
| SAUSA300_0148 | *-* | Hypothetical protein | -3.72 |  |  |
| SAUSA300_0151 | *adhE* | Alcohol dehydrogenase (EC 1.1.1.1) /Acetaldehyde dehydrogenase [acetylating] (EC 1.2.1.10) |  |  |  |
| SAUSA300_0152 | *cap5A* | Hypothetical protein | -11.28 |  | -29.50 |
| SAUSA300_0153 | *cap5B* | Tyrosine-protein kinase (capsular polysaccharide biosynthesis) | -10.69 |  | -30.52 |
| SAUSA300_0154 | *cap5C* | Phosphotyrosine-protein phosphatase (capsular polysaccharide biosynthesis) (EC 3.1.3.48) | -14.03 |  | -26.05 |
| SAUSA300_0156 | *cap5E* | UDP-N-acetylglucosamine 4,6-dehydratase (EC 4.2.1.-) / UDP-2-acetamido-2,6-dideoxy-alpha-D-xylo-4-hexulose 3,5-epimerase (EC 5.1.3.-) | -10.18 |  | -16.06 |
| SAUSA300_0157 | *cap5F* | UDP-2-acetamido-2,6-dideoxy-beta-L-talose 4-dehydrogenase (EC 1.1.1.-) | -7.96 |  | -13.41 |
| SAUSA300_0158 | *cap5G* | UDP-2-acetamido-2,6-dideoxy-beta-L-talose 2-epimerase (EC 5.1.3.-) | -6.95 |  | -9.03 |
| SAUSA300_0159 | *cap5H* | O-acetyl transferase (EC 2.3.1.-) | -7.02 |  | -9.33 |
| SAUSA300_0160 | *cap5I* | Glycosyltransferase (EC 2.4.1.-) | -5.46 |  | -8.24 |
| SAUSA300_0161 | *cap5J* | Capsular polysaccharide synthesis protein Cap5J | -4.71 |  | -6.53 |
| SAUSA300_0162 | *cap5K* | Capsular polysaccharide synthesis protein Cap5K | -5.50 |  | -5.14 |
| SAUSA300_0163 | *cap5L* | Hypothetical protein | -5.09 |  | -4.18 |
| SAUSA300_0164 | *cap5M* | Undecaprenyl-phosphate beta-N-acetyl-D-fucosaminephosphotransferase (EC 2.7.8.-) | -2.51 |  | -3.38 |
| SAUSA300_0165 | *cap5N* | UDP-N-acetyl-D-quinovosamine 4-epimerase (EC 5.1.3.-) | -2.66 |  | -3.44 |
| SAUSA300_0166 | *cap5O* | UDP-N-acetyl-D-mannosamine 6-dehydrogenase (EC 1.1.1.-) | -2.57 |  | -3.28 |
| SAUSA300_0167 | *cap5P* | UDP-N-acetylglucosamine 2-epimerase (EC 5.1.3.14) |  |  |  |
| SAUSA300_0168 | *isdI* | Heme-degrading monooxygenase IsdI |  |  |  |
| SAUSA300_0169 | *-* | Hypothetical membrane spanning protein |  |  |  |
| SAUSA300_0170 | *-* | Aldehyde dehydrogenase B (EC 1.2.1.22) | -3.99 | 3.25 | -5.31 |
| SAUSA300_0171 | *-* | Cobalt-zinc-cadmium resistance protein CzcD |  |  |  |
| SAUSA300_0172 | *-* | Hypothetical protein |  |  |  |
| SAUSA300_0173 | *-* | alpha-helical coiled-coil protein SrpF | -2.01 |  | -2.66 |
| SAUSA300_0174 | *-* | ABC transporter ATP-binding protein | -2.80 |  | -3.84 |
| SAUSA300_0175 | *-* | ABC transporter substrate-binding protein | -3.45 |  | -3.84 |
| SAUSA300_0176 | *-* | ABC transporter permease protein | -2.70 |  | -2.83 |
| SAUSA300_0177 | *-* | Isovaleryl-CoA dehydrogenase (EC 1.3.99.10) | -3.24 |  | -3.28 |
| SAUSA300_0178 | *-* | Hypothetical cytosolic protein | -2.71 |  | -2.37 |
| SAUSA300_0179 | *-* | NAD-dependent formate dehydrogenase (EC 1.2.1.2) | -17.45 |  | -14.05 |
| SAUSA300_0180 | *-* | Multidrug resistance protein ImrP |  |  |  |
| SAUSA300_0181 | *-* | Surfactin synthetase subunit 2 | -2.34 |  |  |
| SAUSA300_0182 | *-* | 4'-phosphopantetheinyl transferase (EC 2.7.8.-) |  |  |  |
| SAUSA300_0183 | *-* | Hypothetical membrane associated protein |  |  |  |
| SAUSA300_0184 | *argB* | Acetylglutamate kinase (EC 2.7.2.8) |  |  |  |
| SAUSA300_0185 | *argJ* | Glutamate N-acetyltransferase (EC 2.3.1.35) / Amino-acid acetyltransferase (EC 2.3.1.1) |  |  |  |
| SAUSA300_0186 | *argC* | N-acetyl-gamma-glutamyl-phosphate reductase (EC 1.2.1.38) |  |  |  |
| SAUSA300_0187 | *rocD* | Ornithine aminotransferase (EC 2.6.1.13) |  |  |  |
| SAUSA300_0188 | *brnQ* | Branched-chain amino acid transport system carrier protein |  |  |  |
| SAUSA300_0189 | *entB* | Isochorismatase family protein |  |  |  |
| SAUSA300_0190 | *ipdC* | Indole-3-pyruvate decarboxylase (EC 4.1.1.74) | -3.91 |  |  |
| SAUSA300_0191 | *ptsG* | PTS system, glucose-specific IIABC component (EC 2.7.1.69) |  |  |  |
| SAUSA300_0192 | *-* | outer surface protein | -23.36 |  | -11.48 |
| SAUSA300_0193 | *murQ* | Glucokinase regulatory protein | -20.63 |  | -14.64 |
| SAUSA300_0194 | *-* | PTS system, sucrose-specific IIBC component (EC 2.7.1.69) | -2.95 |  | -3.44 |
| SAUSA300_0195 | *-* | Transcriptional regulator, RpiR family | -4.60 |  | -3.88 |
| SAUSA300_0196 | *hsdR* | Type I restriction-modification system restriction subunit (EC 3.1.21.3) |  |  |  |
| SAUSA300_0197 | *-* | Hypothetical cytosolic protein |  |  |  |
| SAUSA300_0198 | *-* | Hypothetical cytosolic protein |  |  |  |
| SAUSA300_0199 | *-* | Hypothetical protein |  |  |  |
| SAUSA300_0200 | *-* | Oligopeptide transport ATP-binding protein OppD / Oligopeptide transport ATP-binding protein OppF |  |  |  |
| SAUSA300_0201 | *-* | Oligopeptide transport system permease protein OppB |  |  |  |
| SAUSA300_0202 | *-* | Oligopeptide transport system permease protein OppC |  |  |  |
| SAUSA300_0203 | *-* | Oligopeptide-binding protein OppA |  |  |  |
| SAUSA300_0204 | *ggt* | Gamma-glutamyltranspeptidase (EC 2.3.2.2) |  |  |  |
| SAUSA300_0205 | *-* | membrane lipoprotein | -2.26 |  |  |
| SAUSA300_0206 | *-* | FMN-dependent NADH-azoreductase (EC 1.6.5.2) | 2.20 |  |  |
| SAUSA300_0207 | *-* | Lipoprotein nlpd/lppb homolog |  |  |  |
| SAUSA300_0208 | *-* | Hypothetical protein | -2.88 | 2.45 | -2.70 |
| SAUSA300_0209 | *-* | Maltose/maltodextrin-binding protein | -2.50 | 2.49 | -2.29 |
| SAUSA300_0210 | *-* | Maltodextrin transport system permease protein malC | -2.73 |  |  |
| SAUSA300_0211 | *-* | Hypothetical protein | -2.63 |  |  |
| SAUSA300_0212 | *-* | NAD-dependent oxidoreductase | -2.74 |  |  |
| SAUSA300_0213 | *-* | Hypothetical protein | -2.15 |  |  |
| SAUSA300_0214 | *-* | IolE protein homolog | -2.06 |  |  |
| SAUSA300_0215 | *-* | Hypothetical membrane spanning protein |  |  |  |
| SAUSA300_0216 | *uhpT* | Hexose phosphate transport protein |  |  |  |
| SAUSA300_0217 | *-* | Two-component response regulator VesN |  |  |  |
| SAUSA300_0218 | *-* | Two-component sensor kinase VesM (EC 2.7.3.-) |  |  |  |
| SAUSA300_0219 | *-* | Iron(III)-binding protein |  |  |  |
| SAUSA300_0220 | *pflB* | Formate acetyltransferase (EC 2.3.1.54) |  |  |  |
| SAUSA300_0221 | *pflA* | Pyruvate formate-lyase activating enzyme (EC 1.97.1.4) |  |  |  |
| SAUSA300_0222 | *-* | Hypothetical protein / Glycerophosphodiester phosphodiesterase (EC 3.1.4.46) |  |  |  |
| SAUSA300_0223 | *-* | Hypothetical protein |  |  |  |
| SAUSA300_0224 | *coa* | Staphylocoagulase precursor |  |  |  |
| SAUSA300_0225 | *-* | 3-ketoacyl-CoA thiolase (EC 2.3.1.16) |  |  |  |
| SAUSA300_0226 | *-* | Enoyl-CoA hydratase (EC 4.2.1.17) / Delta(3)-cis-delta(2)-trans-enoyl-CoA isomerase (EC 5.3.3.8) / 3-hydroxyacyl-CoA dehydrogenase (EC 1.1.1.35) |  |  |  |
| SAUSA300_0227 | *fadD* | Glutaryl-CoA dehydrogenase (EC 1.3.99.7) |  |  |  |
| SAUSA300_0228 | *fadE* | Long-chain-fatty-acid--CoA ligase (EC 6.2.1.3) |  |  |  |
| SAUSA300_0229 | *-* | Acetyl-CoA:acetoacetyl-CoA transferase alpha subunit (EC 2.8.3.-) | -2.83 |  | -2.61 |
| SAUSA300_0230 | *-* | Hypothetical membrane spanning protein |  |  |  |
| SAUSA300_0231 | *-* | Dipeptide-binding protein |  |  |  |
| SAUSA300_0232 | *-* | Hypothetical cytosolic protein |  |  |  |
| SAUSA300_0233 | *-* | Hypothetical protein |  |  |  |
| SAUSA300_0234 | *-* | Flavohemoprotein / Dihydropteridine reductase (EC 1.5.1.34) / Nitric oxide dioxygenase (EC 1.14.12.17) |  |  |  |
| SAUSA300_0235 | *-* | L-lactate dehydrogenase (EC 1.1.1.27) |  |  |  |
| SAUSA300_0236 | *-* | PTS system, glucose-specific IIBC component (EC 2.7.1.69) | 3.04 |  |  |
| SAUSA300_0237 | *-* | Inosine-uridine preferring nucleoside hydrolase (EC 3.2.2.1) |  |  |  |
| SAUSA300_0238 | *-* | Transcription antiterminator, BglG family / PTS system, mannitol (Cryptic)-specific IIA component (EC 2.7.1.69) |  |  |  |
| SAUSA300_0239 | *-* | PTS system, galactitol-specific IIA component (EC 2.7.1.69) |  |  |  |
| SAUSA300_0240 | *-* | PTS system, galactitol-specific IIB component (EC 2.7.1.69) |  |  |  |
| SAUSA300_0241 | *-* | PTS system, galactitol-specific IIC component (EC 2.7.1.69) |  |  |  |
| SAUSA300_0242 | *gutB* | Sorbitol dehydrogenase (EC 1.1.1.14) |  |  |  |
| SAUSA300_0243 | *-* | Hypothetical protein |  |  |  |
| SAUSA300_0244 | *-* | Sorbitol dehydrogenase (EC 1.1.1.14) |  |  |  |
| SAUSA300_0245 | *-* | D-ribitol-5-phosphate cytidylyltransferase (EC 2.7.7.40) |  |  |  |
| SAUSA300_0246 | *-* | Ribitol-5-phosphate 2-dehydrogenase (EC 1.1.1.137) |  |  |  |
| SAUSA300_0247 | *-* | CDP-ribitol ribitolphosphotransferase (EC 2.7.8.14) |  |  |  |
| SAUSA300_0248 | *-* | CDP-glycerol glycerophosphotransferase (EC 2.7.8.12) |  |  |  |
| SAUSA300_0249 | *ispD* | D-ribitol-5-phosphate cytidylyltransferase (EC 2.7.7.40) |  |  |  |
| SAUSA300_0250 | *-* | Ribitol-5-phosphate 2-dehydrogenase (EC 1.1.1.137) |  |  |  |
| SAUSA300_0251 | *-* | CDP-ribitol ribitolphosphotransferase (EC 2.7.8.14) |  |  |  |
| SAUSA300_0252 | *-* | Hypothetical protein |  |  |  |
| SAUSA300_0253 | *scdA* | Nitric oxide-dependent regulator DnrN |  |  |  |
| SAUSA300_0254 | *-* | Autolysin sensor kinase (EC 2.7.3.-) |  |  |  |
| SAUSA300_0255 | *-* | Autolysin response regulator |  |  |  |
| SAUSA300_0256 | *lrgA* | Murein hydrolase exporter |  |  |  |
| SAUSA300_0257 | *lrgB* | Murein hydrolase export regulator |  |  |  |
| SAUSA300_0258 | *-* | Hypothetical protein |  |  |  |
| SAUSA300_0259 | *-* | PTS system, beta-glucoside-specific IIABC component (EC 2.7.1.69) |  |  |  |
| SAUSA300_0260 | *bglA* | 6-phospho-beta-glucosidase (EC 3.2.1.86) |  |  |  |
| SAUSA300_0261 | *-* | Hypothetical protein |  |  |  |
| SAUSA300_0262 | *rbsK* | Ribokinase (EC 2.7.1.15) |  |  |  |
| SAUSA300_0263 | *rbsD* | D-ribose mutarotase (EC 5.1.3.-) |  |  |  |
| SAUSA300_0264 | *-* | Ribose uptake protein |  |  |  |
| SAUSA300_0265 | *-* | Transcriptional repressor |  |  |  |
| SAUSA300_0266 | *-* | Hypothetical cytosolic protein | 2.05 |  |  |
| SAUSA300_0267 | *-* | Transposase |  |  |  |
| SAUSA300_0268 | *-* | Multidrug resistance protein B |  |  |  |
| SAUSA300_0269 | *-* | Choloylglycine hydrolase (EC 3.5.1.24) |  |  |  |
| SAUSA300_0270 | *lytM* | Peptidoglycan hydrolase |  |  |  |
| SAUSA300_0271 | *-* | ABC transporter ATP-binding protein |  |  |  |
| SAUSA300_0272 | *-* | Hypothetical protein |  |  |  |
| SAUSA300_0273 | *-* | Hypothetical protein |  |  |  |
| SAUSA300_0274 | *-* | Hypothetical protein |  |  |  |
| SAUSA300_0275 | *-* | Hypothetical protein |  |  |  |
| SAUSA300_0276 | *-* | Hypothetical protein |  |  |  |
| SAUSA300_0277 | *-* | Secretory antigen precursor SsaA | -2.66 |  | -4.28 |
| SAUSA300_0278 | *-* | Hypothetical protein |  |  |  |
| SAUSA300_0279 | *-* | Hypothetical membrane spanning protein | -2.15 |  | -2.52 |
| SAUSA300_0280 | *-* | Hypothetical protein |  |  |  |
| SAUSA300_0281 | *-* | Hypothetical protein | -2.07 |  | -2.65 |
| SAUSA300_0282 | *-* | Hypothetical membrane associated protein |  |  | -2.65 |
| SAUSA300_0283 | *-* | DNA segregation ATPase and related proteins (FtsK/SpoIIIE family) | -2.26 |  | -2.22 |
| SAUSA300_0284 | *-* | Hypothetical protein | -3.21 |  |  |
| SAUSA300_0285 | *-* | Hypothetical protein | -2.47 |  | -2.62 |
| SAUSA300_0286 | *-* | Hypothetical protein | -2.60 |  | -2.52 |
| SAUSA300_0287 | *-* | Hypothetical protein |  |  |  |
| SAUSA300_0288 | *-* | Hypothetical cytosolic protein |  |  |  |
| SAUSA300_0289 | *-* | Hypothetical cytosolic protein |  |  |  |
| SAUSA300_0290 | *-* | Hypothetical protein |  |  |  |
| SAUSA300_0291 | *-* | Hypothetical protein |  |  |  |
| SAUSA300_0292 | *-* | Hypothetical protein | -2.88 |  | -2.67 |
| SAUSA300_0293 | *-* | Hypothetical cytosolic protein | -2.54 |  | -2.57 |
| SAUSA300_0294 | *-* | Hypothetical cytosolic protein | -2.02 |  | -3.08 |
| SAUSA300_0295 | *-* | Hypothetical cytosolic protein |  |  |  |
| SAUSA300_0296 | *-* | Hypothetical cytosolic protein |  |  |  |
| SAUSA300_0297 | *-* | Hypothetical protein |  |  |  |
| SAUSA300_0298 | *-* | Hypothetical cytosolic protein | 2.94 |  |  |
| SAUSA300_0299 | *-* | Hypothetical cytosolic protein |  |  |  |
| SAUSA300_0300 | *-* | Hypothetical cytosolic protein |  |  |  |
| SAUSA300_0301 | *-* | Hypothetical cytosolic protein |  |  |  |
| SAUSA300_0302 | *-* | Hypothetical cytosolic protein |  |  |  |
| SAUSA300_0303 | *-* | Hypothetical protein |  |  | -2.26 |
| SAUSA300_0304 | *-* | Hypothetical protein | -2.20 |  |  |
| SAUSA300_0305 | *-* | Formate/nitrite transporter family protein |  |  |  |
| SAUSA300_0306 | *brnQ* | Branched-chain amino acid transport system carrier protein |  |  |  |
| SAUSA300_0307 | *-* | Acid phosphatase (EC 3.1.3.2) |  |  |  |
| SAUSA300_0308 | *-* | ABC transporter permease protein |  |  |  |
| SAUSA300_0309 | *-* | ABC transporter ATP-binding protein |  |  |  |
| SAUSA300_0310 | *pfoR* | Transcriptional regulator PfoR |  |  |  |
| SAUSA300_0311 | *-* | Ribokinase (EC 2.7.1.15) |  |  |  |
| SAUSA300_0312 | *-* | Sugar kinases |  |  |  |
| SAUSA300_0313 | *-* | Nucleoside permease NupC |  |  |  |
| SAUSA300_0314 | *-* | Hypothetical protein |  |  |  |
| SAUSA300_0315 | *nanA* | N-acetylneuraminate lyase (EC 4.1.3.3) |  |  |  |
| SAUSA300_0316 | *-* | Glucokinase (EC 2.7.1.2) |  | 2.73 | -2.91 |
| SAUSA300_0317 | *-* | Transcriptional regulator, RpiR family |  |  |  |
| SAUSA300_0318 | *-* | N-acetylmannosamine-6-phosphate 2-epimerase (EC 5.1.3.9) |  |  |  |
| SAUSA300_0319 | *-* | Hypothetical membrane spanning protein |  |  |  |
| SAUSA300_0320 | *-* | Lipase (EC 3.1.1.3) | -7.97 |  | -7.39 |
| SAUSA300_0321 | *-* | Acetyl esterase (EC 3.1.1.-) |  |  |  |
| SAUSA300_0322 | *-* | Probable NADH-dependent flavin oxidoreductase VqiG (EC 1.-.-.-) |  |  |  |
| SAUSA300_0323 | *-* | Hypothetical protein |  |  |  |
| SAUSA300_0324 | *-* | Luciferase-like monooxygenase (EC 1.14.-.-) |  |  |  |
| SAUSA300_0325 | *-* | Glycine cleavage system H protein |  |  |  |
| SAUSA300_0326 | *-* | ATPase associated with chromosome architecture/replication |  |  |  |
| SAUSA300_0327 | *-* | SIR2 family protein |  |  |  |
| SAUSA300_0328 | *-* | Lipoate-protein ligase A (EC 6.3.2.-) |  |  |  |
| SAUSA300_0329 | *-* | Cprd14 protein |  |  |  |
| SAUSA300_0330 | *ulaA* | Putative transport protein SgaT | -3.21 |  |  |
| SAUSA300_0331 | *-* | Pts system, IIB component (ec 2.7.1.69) | -2.11 |  |  |
| SAUSA300_0332 | *-* | PTS system, mannitol-specific IIA component (EC 2.7.1.69) | -2.79 |  |  |
| SAUSA300_0333 | *-* | Transcription antiterminator, BglG family / PTS system, mannitol (Cryptic)-specific IIA component (EC 2.7.1.69) | -3.09 | 2.93 |  |
| SAUSA300_0334 | *-* | Transcriptional regulator, MarR family | 4.51 | 2.28 |  |
| SAUSA300_0335 | *-* | Na+ driven multidrug efflux pump | 3.71 | 2.77 |  |
| SAUSA300_0336 | *-* | Hypothetical cytosolic protein |  |  |  |
| SAUSA300_0337 | *glpT* | Glycerol-3-phosphate transporter |  |  |  |
| SAUSA300_0338 | *-* | Hypothetical protein |  |  |  |
| SAUSA300_0339 | *-* | Luciferase-like monooxygenase (EC 1.14.-.-) |  |  |  |
| SAUSA300_0340 | *-* | FMN reductase (EC 1.5.1.29) |  |  |  |
| SAUSA300_0341 | *-* | Hypothetical membrane spanning protein |  |  |  |
| SAUSA300_0342 | *-* | Hypothetical protein |  |  |  |
| SAUSA300_0343 | *-* | Ribosomal-protein-serine acetyltransferase (EC 2.3.1.-) | 2.24 |  |  |
| SAUSA300_0344 | *-* | Lipoprotein |  |  |  |
| SAUSA300_0345 | *-* | Dyp-type peroxidase family protein |  |  |  |
| SAUSA300_0346 | *-* | High-affinity iron permease |  |  |  |
| SAUSA300_0347 | *tatC* | Sec-independent protein translocase protein TatC |  |  |  |
| SAUSA300_0348 | *-* | Sec-independent protein translocase protein TatA |  |  |  |
| SAUSA300_0349 | *-* | Sec-independent protein translocase protein TatA |  |  |  |
| SAUSA300_0350 | *-* | Transcriptional regulator, Cro/CI family |  |  |  |
| SAUSA300_0351 | *-* | ABC transporter permease protein |  |  |  |
| SAUSA300_0352 | *-* | ABC transporter ATP-binding protein |  |  |  |
| SAUSA300_0353 | *-* | ABC transporter permease protein |  |  |  |
| SAUSA300_0354 | *ltrA* | Low temperature requirement protein A |  |  |  |
| SAUSA300_0355 | *-* | Acetyl-CoA acetyltransferase (EC 2.3.1.9) |  |  |  |
| SAUSA300_0356 | *-* | Polyketide cyclase family protein |  |  |  |
| SAUSA300_0357 | *metE* | 5-methyltetrahydropteroyltriglutamate--homocysteine methyltransferase (EC 2.1.1.14) |  |  |  |
| SAUSA300_0358 | *-* | 5-methyltetrahydrofolate--homocysteine methyltransferase (EC 2.1.1.13) homocysteine-binding subunit / Methylenetetrahydrofolate reductase (EC 1.5.1.20) | -2.30 | -3.08 | 2.03 |
| SAUSA300_0359 | *-* | Cystathionine beta-lyase (EC 4.4.1.8) | -2.14 | -2.85 |  |
| SAUSA300_0360 | *-* | Cystathionine gamma-synthase (EC 2.5.1.48) | -3.33 | -5.61 |  |
| SAUSA300_0361 | *-* | Chromosome partitioning protein parB |  |  |  |
| SAUSA300_0362 | *-* | Mechanosensitive ion channel |  |  |  |
| SAUSA300_0363 | *-* | Hypothetical cytosolic protein |  |  |  |
| SAUSA300_0364 | *ychF* | GTP-binding protein, probable translation factor |  |  |  |
| SAUSA300_0365 | *-* | Hypothetical protein |  | -2.02 | 2.05 |
| SAUSA300_0366 | *rpsF* | SSU ribosomal protein S6P |  |  |  |
| SAUSA300_0367 | *ssb* | Single-strand DNA binding protein |  |  |  |
| SAUSA300_0368 | *rpsR* | SSU ribosomal protein S18P |  |  |  |
| SAUSA300_0369 | *-* | Hypothetical protein |  |  |  |
| SAUSA300_0370 | *-* | Exotoxin |  |  |  |
| SAUSA300_0371 | *-* | Hypothetical protein |  |  |  |
| SAUSA300_0372 | *-* | Hypothetical exported protein |  |  |  |
| SAUSA300_0373 | *-* | Hypothetical protein |  |  |  |
| SAUSA300_0374 | *-* | Hypothetical membrane spanning protein |  |  |  |
| SAUSA300_0375 | *-* | Phosphoglycerate mutase family protein |  |  |  |
| SAUSA300_0376 | *-* | Hypothetical membrane spanning protein |  |  |  |
| SAUSA300_0377 | *-* | Hypothetical protein |  |  |  |
| SAUSA300_0378 | *-* | Hypothetical protein |  |  |  |
| SAUSA300_0379 | *ahpF* | Peroxiredoxin reductase (NAD(P)H) (EC 1.8.1.-) / NADH oxidase H2O2-forming (EC 1.6.3.-) |  |  |  |
| SAUSA300_0380 | *ahpC* | Peroxiredoxin (EC 1.11.1.15) |  |  |  |
| SAUSA300_0381 | *-* | Chromate reductase (EC 1.-.-.-) / NADPH-dependent FMN reductase (EC 1.5.1.-) / Oxygen-insensitive NADPH nitroreductase (EC 1.-.-.-) |  |  |  |
| SAUSA300_0382 | *-* | Sodium:dicarboxylate symporter family protein VhcL |  |  |  |
| SAUSA300_0383 | *-* | Hypothetical protein |  |  |  |
| SAUSA300_0384 | *-* | Hypothetical protein |  |  |  |
| SAUSA300_0385 | *-* | Transcriptional regulator |  |  |  |
| SAUSA300_0386 | *xpt* | Xanthine phosphoribosyltransferase (EC 2.4.2.-) |  |  |  |
| SAUSA300_0387 | *pbuX* | Xanthine permease |  |  |  |
| SAUSA300_0388 | *guaB* | Inosine-5'-monophosphate dehydrogenase (EC 1.1.1.205) |  |  |  |
| SAUSA300_0389 | *guaA* | GMP synthase [glutamine-hydrolyzing] (EC 6.3.5.2) |  |  |  |
| SAUSA300_0390 | *-* | Hypothetical protein |  |  |  |
| SAUSA300_0391 | *-* | Phage protein |  |  |  |
| SAUSA300_0392 | *-* | Hypothetical protein |  |  |  |
| SAUSA300_0393 | *-* | Hypothetical membrane spanning protein |  |  |  |
| SAUSA300_0394 | *-* | putative nucleoside-diphosphate-sugar epimerases |  |  |  |
| SAUSA300_0395 | *-* | Exotoxin |  |  |  |
| SAUSA300_0396 | *set7* | Exotoxin | 2.11 |  |  |
| SAUSA300_0397 | *-* | Exotoxin |  |  |  |
| SAUSA300_0398 | *-* | Exotoxin |  |  |  |
| SAUSA300_0399 | *-* | Exotoxin |  |  |  |
| SAUSA300_0400 | *-* | Hypothetical protein |  |  |  |
| SAUSA300_0401 | *-* | Exotoxin |  |  |  |
| SAUSA300_0402 | *-* | Exotoxin |  |  |  |
| SAUSA300_0403 | *-* | Exotoxin |  |  |  |
| SAUSA300_0404 | *-* | Exotoxin |  |  |  |
| SAUSA300_0405 | *hsdM* | Type I restriction-modification system methylation subunit |  |  |  |
| SAUSA300_0406 | *-* | Type I restriction-modification system specificity subunit |  |  |  |
| SAUSA300_0407 | *-* | Exotoxin |  |  |  |
| SAUSA300_0408 | *-* | Hypothetical cytosolic protein |  |  |  |
| SAUSA300_0409 | *-* | Hypothetical exported protein | -2.52 |  | -2.14 |
| SAUSA300_0410 | *-* | membrane lipoprotein |  |  |  |
| SAUSA300_0411 | *-* | membrane lipoprotein |  |  |  |
| SAUSA300_0413 | *-* | membrane lipoprotein |  |  |  |
| SAUSA300_0414 | *-* | membrane lipoprotein |  |  |  |
| SAUSA300_0415 | *lpl3* | membrane lipoprotein |  |  |  |
| SAUSA300_0416 | *-* | membrane lipoprotein |  |  |  |
| SAUSA300_0417 | *-* | membrane lipoprotein |  |  |  |
| SAUSA300_0418 | *-* | membrane lipoprotein |  |  |  |
| SAUSA300_0419 | *-* | membrane lipoprotein |  |  | -2.18 |
| SAUSA300_0420 | *-* | Hypothetical cytosolic protein |  |  |  |
| SAUSA300_0421 | *-* | Hypothetical cytosolic protein |  |  |  |
| SAUSA300_0422 | *-* | Hypothetical protein |  |  |  |
| SAUSA300_0423 | *-* | Hypothetical protein |  |  |  |
| SAUSA300_0424 | *-* | Low-affinity zinc transport protein |  |  |  |
| SAUSA300_0425 | *-* | NADH-quinone oxidoreductase chain L (EC 1.6.5.3) | 2.07 |  |  |
| SAUSA300_0426 | *-* | Hypothetical protein |  |  |  |
| SAUSA300_0427 | *-* | Hypothetical cytosolic protein |  |  |  |
| SAUSA300_0428 | *-* | Hypothetical protein |  |  |  |
| SAUSA300_0429 | *-* | Phosphatidylglycerophosphatase B homolog |  |  |  |
| SAUSA300_0430 | *-* | Carboxylesterase (EC 3.1.1.1) |  |  |  |
| SAUSA300_0431 | *-* | Hypothetical protein |  |  |  |
| SAUSA300_0432 | *-* | Sodium/neurotransmitter symporter family protein |  | 2.37 |  |
| SAUSA300_0433 | *cysM* | Cysteine synthase (EC 2.5.1.47) |  |  | -2.13 |
| SAUSA300_0434 | *metB* | Cystathionine beta-lyase (EC 4.4.1.8) / Cystathionine gamma-lyase (EC 4.4.1.1) |  |  | -2.03 |
| SAUSA300_0435 | *-* | ABC transporter ATP-binding protein |  |  |  |
| SAUSA300_0436 | *-* | ABC transporter permease protein |  |  |  |
| SAUSA300_0437 | *-* | ABC transporter substrate-binding protein |  |  |  |
| SAUSA300_0438 | *-* | Putative endopeptidase LytE precursor |  |  |  |
| SAUSA300_0439 | *-* | Hypothetical cytosolic protein |  |  |  |
| SAUSA300_0440 | *-* | Phosphohydrolase (MutT/nudix family protein) |  |  |  |
| SAUSA300_0441 | *-* | Acetyltransferase (EC 2.3.1.-) |  |  |  |
| SAUSA300_0442 | *-* | Hypothetical membrane spanning protein |  |  |  |
| SAUSA300_0443 | *-* | Hypothetical membrane spanning protein |  |  |  |
| SAUSA300_0444 | *gltC* | Transcriptional regulatory protein gltC |  |  |  |
| SAUSA300_0445 | *gltB* | Glutamate synthase [NADPH] large chain (EC 1.4.1.13) | 3.90 |  | 2.85 |
| SAUSA300_0446 | *gltD* | Glutamate synthase [NADPH] small chain (EC 1.4.1.13) | 2.76 |  | 2.30 |
| SAUSA300_0448 | *treP* | PTS system, trehalose-specific IIBC component (EC 2.7.1.69) |  |  |  |
| SAUSA300_0449 | *treC* | Trehalose-6-phosphate hydrolase (EC 3.2.1.93) |  |  |  |
| SAUSA300_0450 | *treR* | Trehalose operon transcriptional repressor |  |  |  |
| SAUSA300_0451 | *-* | Acetyltransferase (EC 2.3.1.-) |  |  |  |
| SAUSA300_0452 | *dnaX* | DNA polymerase III subunit gamma/tau (EC 2.7.7.7) |  |  |  |
| SAUSA300_0453 | *-* | Hypothetical transcriptional regulatory protein |  |  |  |
| SAUSA300_0454 | *recR* | Recombination protein recR |  |  |  |
| SAUSA300_0458 | *-* | Arginine decarboxylase (EC 4.1.1.19) |  |  |  |
| SAUSA300_0459 | *tmk* | Thymidylate kinase (EC 2.7.4.9) |  |  |  |
| SAUSA300_0460 | *-* | Hypothetical cytosolic protein |  |  |  |
| SAUSA300_0461 | *holB* | DNA polymerase III, delta' subunit (EC 2.7.7.7) |  |  |  |
| SAUSA300_0462 | *-* | Phosphorelay inhibitor |  |  |  |
| SAUSA300_0463 | *-* | Initiation-control protein |  |  |  |
| SAUSA300_0464 | *-* | Methyltransferase (EC 2.1.1.-) |  |  |  |
| SAUSA300_0465 | *-* | Hypothetical protein with endo/excinuclease domain |  |  |  |
| SAUSA300_0466 | *-* | Tetrapyrrole (Corrin/Porphyrin) methylase family protein |  |  |  |
| SAUSA300_0467 | *metS* | Methionyl-tRNA synthetase (EC 6.1.1.10) / Protein secretion chaperonin CsaA |  |  |  |
| SAUSA300_0468 | *-* | DNase, TatD family (EC 3.1.-.-) |  |  |  |
| SAUSA300_0469 | *-* | Ribonuclease M5 (EC 3.1.26.8) |  |  |  |
| SAUSA300_0470 | *ksgA* | Dimethyladenosine transferase (EC 2.1.1.-) |  |  |  |
| SAUSA300_0471 | *-* | VEG protein |  |  |  |
| SAUSA300_0472 | *ipk* | 4-diphosphocytidyl-2-C-methyl-D-erythritol kinase (EC 2.7.1.148) |  |  |  |
| SAUSA300_0473 | *purR* | Pur operon repressor |  |  |  |
| SAUSA300_0474 | *-* | Translation initiation inhibitor |  |  |  |
| SAUSA300_0475 | *-* | Stage V sporulation protein G |  |  |  |
| SAUSA300_0476 | *-* | Hypothetical protein |  |  |  |
| SAUSA300_0477 | *glmU* | Glucosamine-1-phosphate acetyltransferase (EC 2.3.1.157) / UDP-N-acetylglucosamine pyrophosphorylase (EC 2.7.7.23) |  |  |  |
| SAUSA300_0478 | *prs* | Ribose-phosphate pyrophosphokinase (EC 2.7.6.1) |  |  |  |
| SAUSA300_0479 | *-* | LSU ribosomal protein L25P |  |  |  |
| SAUSA300_0480 | *pth* | Peptidyl-tRNA hydrolase (EC 3.1.1.29) |  |  |  |
| SAUSA300_0481 | *mfd* | Transcription-repair coupling factor |  |  |  |
| SAUSA300_0482 | *-* | Export protein for polysaccharides and teichoic acids |  |  |  |
| SAUSA300_0483 | *-* | Hypothetical protein |  |  |  |
| SAUSA300_0484 | *-* | Heat shock protein 15 |  |  |  |
| SAUSA300_0485 | *-* | Cell division protein DIVIC |  |  |  |
| SAUSA300_0486 | *-* | S1-type RNA-binding domain |  |  |  |
| SAUSA300_0487 | *-* | tRNA(Ile)-lysidine synthetase TilS |  |  |  |
| SAUSA300_0488 | *hpt* | Hypoxanthine-guanine phosphoribosyltransferase (EC 2.4.2.8) |  |  |  |
| SAUSA300_0489 | *-* | Cell division protein ftsH (EC 3.4.24.-) |  |  |  |
| SAUSA300_0490 | *hslO* | 33 kDa chaperonin |  |  |  |
| SAUSA300_0491 | *cysK* | Cysteine synthase (EC 2.5.1.47) |  |  |  |
| SAUSA300_0492 | *folP* | Dihydropteroate synthase (EC 2.5.1.15) |  |  |  |
| SAUSA300_0493 | *folB* | Dihydroneopterin aldolase (EC 4.1.2.25) |  |  |  |
| SAUSA300_0494 | *folK* | 2-amino-4-hydroxy-6-hydroxymethyldihydropteridine pyrophosphokinase (EC 2.7.6.3) |  |  |  |
| SAUSA300_0495 | *-* | Hypothetical protein |  |  |  |
| SAUSA300_0496 | *lysS* | Lysyl-tRNA synthetase (EC 6.1.1.6) |  |  |  |
| SAUSA300_0503 | *-* | Transcriptional regulator, gntr family / aminotransferase class-i (ec 2.6.1.-) |  |  |  |
| SAUSA300_0504 | *-* | pyridoxine biosynthesis protein |  |  |  |
| SAUSA300_0505 | *-* | pyridoxine biosynthesis amidotransferase (EC 2.4.2.-) |  |  |  |
| SAUSA300_0506 | *nupC* | Nucleoside permease NupC |  |  |  |
| SAUSA300_0507 | *ctsR* | Transcriptional regulator CtsR |  |  |  |
| SAUSA300_0508 | *-* | Clpc atpase |  |  |  |
| SAUSA300_0509 | *-* | Arginine kinase (EC 2.7.3.3) |  |  |  |
| SAUSA300_0510 | *clpC* | Negative regulator of genetic competence *clpC*/*mecB* / Hemolysin *tlyB* |  |  |  |
| SAUSA300_0511 | *radA* | DNA repair protein RadA |  |  |  |
| SAUSA300_0512 | *-* | Hypothetical protein |  |  |  |
| SAUSA300_0513 | *gltX* | Glutamyl-tRNA synthetase (EC 6.1.1.17) |  |  |  |
| SAUSA300_0514 | *cysE* | Serine acetyltransferase (EC 2.3.1.30) |  |  |  |
| SAUSA300_0515 | *cysS* | Cysteinyl-tRNA synthetase (EC 6.1.1.16) |  |  |  |
| SAUSA300_0516 | *-* | Hypothetical protein |  |  |  |
| SAUSA300_0517 | *-* | 23S rRNA Gm2251 methyltransferase (EC 2.1.1.-) |  |  |  |
| SAUSA300_0518 | *-* | Hypothetical cytosolic protein |  |  |  |
| SAUSA300_0519 | *-* | RNA polymerase sigma-H factor |  |  |  |
| SAUSA300_0520 | *secE* | Protein translocase subunit secE |  |  |  |
| SAUSA300_0521 | *nusG* | Transcription antitermination protein nusG |  |  |  |
| SAUSA300_0522 | *rplK* | LSU ribosomal protein L11P |  |  |  |
| SAUSA300_0523 | *rplA* | LSU ribosomal protein L1P |  |  |  |
| SAUSA300_0524 | *rplJ* | LSU ribosomal protein L10P |  |  | 2.44 |
| SAUSA300_0525 | *rplL* | LSU ribosomal protein L12P (L7/L12) | 2.04 |  | 3.28 |
| SAUSA300_0526 | *-* | 16S rRNA m(2)G 1207 methyltransferase (EC 2.1.1.52) |  |  |  |
| SAUSA300_0527 | *rpoB* | DNA-directed RNA polymerase beta chain (EC 2.7.7.6) |  |  |  |
| SAUSA300_0528 | *rpoC* | DNA-directed RNA polymerase beta' chain (EC 2.7.7.6) |  |  |  |
| SAUSA300_0529 | *-* | LSU ribosomal protein L7AE |  |  |  |
| SAUSA300_0530 | *rpsL* | SSU ribosomal protein S12P |  |  |  |
| SAUSA300_0531 | *-* | SSU ribosomal protein S7P |  |  |  |
| SAUSA300_0532 | *fusA* | Protein Translation Elongation Factor G (EF-G) |  |  |  |
| SAUSA300_0533 | *tuf* | Protein Translation Elongation Factor Tu (EF-TU) |  |  |  |
| SAUSA300_0534 | *-* | N-acyl-L-amino acid amidohydrolase (EC 3.5.1.14) |  |  |  |
| SAUSA300_0535 | *-* | 2-amino-3-ketobutyrate coenzyme A ligase (EC 2.3.1.29) |  |  |  |
| SAUSA300_0536 | *-* | ThiJ/PfpI family protein |  |  |  |
| SAUSA300_0537 | *-* | L-ribulokinase (EC 2.7.1.16) |  |  |  |
| SAUSA300_0538 | *-* | L-threonine 3-dehydrogenase (EC 1.1.1.103) |  |  |  |
| SAUSA300_0539 | *ilvE* | Branched-chain amino acid aminotransferase (EC 2.6.1.42) |  |  |  |
| SAUSA300_0540 | *-* | Putative phosphatase |  |  |  |
| SAUSA300_0541 | *-* | Deoxyadenosine kinase (EC 2.7.1.76) / Deoxycytidine kinase (EC 2.7.1.74) |  |  |  |
| SAUSA300_0542 | *-* | Deoxyguanosine kinase (EC 2.7.1.113) |  |  |  |
| SAUSA300_0543 | *-* | tRNA-specific adenosine deaminase (EC 3.5.4.-) |  |  |  |
| SAUSA300_0544 | *-* | Hydrolase (HAD superfamily) | 2.19 |  |  |
| SAUSA300_0545 | *-* | Acyl carrier protein phosphodiesterase (EC 3.1.4.14) | 2.10 |  |  |
| SAUSA300_0546 | *sdrC* | Fibronectin-binding protein |  |  |  |
| SAUSA300_0547 | *sdrD* | Fibronectin-binding protein | 3.53 |  | 4.56 |
| SAUSA300_0548 | *sdrE* | Fibronectin-binding protein |  |  |  |
| SAUSA300_0549 | *-* | Poly(Glycerol-phosphate) alpha-glucosyltransferase (EC 2.4.1.52) |  |  |  |
| SAUSA300_0550 | *-* | Poly(Glycerol-phosphate) alpha-glucosyltransferase (EC 2.4.1.52) |  |  |  |
| SAUSA300_0551 | *-* | Hypothetical cytosolic protein |  |  |  |
| SAUSA300_0552 | *-* | GlcNAc-PI de-N-acetylase family protein |  |  |  |
| SAUSA300_0553 | *-* | Hypothetical protein |  |  |  |
| SAUSA300_0554 | *-* | Glucosamine-6-phosphate isomerase (EC 3.5.99.6) |  |  |  |
| SAUSA300_0555 | *-* | Hexulose-6-phosphate synthase (EC 4.1.2.-) |  |  |  |
| SAUSA300_0556 | *-* | Hexulose-6-phosphate isomerase (EC 5.-.-.-) |  |  |  |
| SAUSA300_0557 | *-* | Putative phosphatase |  |  |  |
| SAUSA300_0558 | *-* | Proline/betaine transporter |  |  |  |
| SAUSA300_0559 | *-* | Long-chain-fatty-acid--CoA ligase (EC 6.2.1.3) |  |  |  |
| SAUSA300_0560 | *vraB* | Acetyl-CoA acetyltransferase (EC 2.3.1.9) |  |  |  |
| SAUSA300_0561 | *-* | Hypothetical cytosolic protein |  |  |  |
| SAUSA300_0562 | *thiD* | Phosphomethylpyrimidine kinase (EC 2.7.4.7) / Hydroxymethylpyrimidine kinase (EC 2.7.1.49) |  |  |  |
| SAUSA300_0563 | *ung* | Uracil-DNA glycosylase (EC 3.2.2.-) |  |  |  |
| SAUSA300_0564 | *-* | Hypothetical protein |  |  |  |
| SAUSA300_0565 | *-* | Hypothetical membrane spanning protein |  |  |  |
| SAUSA300_0566 | *-* | Arginine permease |  |  |  |
| SAUSA300_0567 | *-* | Threonine/Serine Exporter |  |  |  |
| SAUSA300_0568 | *-* | Threonine/Serine Exporter |  |  |  |
| SAUSA300_0569 | *-* | Hypothetical protein |  |  |  |
| SAUSA300_0570 | *eutD* | Phosphate acetyltransferase (EC 2.3.1.8) |  |  |  |
| SAUSA300_0571 | *-* | Lipoate-protein ligase A (EC 6.3.2.-) |  |  |  |
| SAUSA300_0572 | *mvk* | Mevalonate kinase (EC 2.7.1.36) |  |  |  |
| SAUSA300_0573 | *mvaD* | Diphosphomevalonate decarboxylase (EC 4.1.1.33) |  |  |  |
| SAUSA300_0574 | *-* | Phosphomevalonate kinase (EC 2.7.4.2) |  |  |  |
| SAUSA300_0575 | *-* | Hypothetical cytosolic protein |  |  |  |
| SAUSA300_0576 | *-* | Pyridine nucleotide-disulphide oxidoreductase family protein (EC 1.-.-.-) |  |  |  |
| SAUSA300_0577 | *-* | Rrf2 family protein |  |  |  |
| SAUSA300_0578 | *-* | Hypothetical protein |  |  |  |
| SAUSA300_0579 | *-* | Phage protein |  |  | -2.09 |
| SAUSA300_0580 | *-* | Hypothetical membrane spanning protein |  |  |  |
| SAUSA300_0581 | *-* | Hypothetical membrane spanning protein |  |  |  |
| SAUSA300_0582 | *-* | Hypothetical membrane spanning protein |  |  |  |
| SAUSA300_0583 | *-* | Hypothetical membrane spanning protein | -2.95 |  |  |
| SAUSA300_0584 | *-* | Hypothetical membrane spanning protein | -2.20 |  |  |
| SAUSA300_0585 | *-* | Hypothetical membrane spanning protein |  |  |  |
| SAUSA300_0586 | *-* | Hypothetical membrane spanning protein |  |  |  |
| SAUSA300_0587 | *-* | Hypothetical membrane spanning protein |  |  |  |
| SAUSA300_0588 | *-* | Hypothetical membrane spanning protein |  |  |  |
| SAUSA300_0589 | *-* | IolS protein |  |  |  |
| SAUSA300_0590 | *-* | Trp repressor binding protein |  |  |  |
| SAUSA300_0591 | *-* | Hypothetical protein |  |  |  |
| SAUSA300_0592 | *-* | dGTP triphosphohydrolase |  |  |  |
| SAUSA300_0593 | *-* | Hypothetical cytosolic protein |  |  |  |
| SAUSA300_0594 | *adh* | Alcohol dehydrogenase (EC 1.1.1.1) |  |  |  |
| SAUSA300_0595 | *-* | Hypothetical protein |  |  |  |
| SAUSA300_0596 | *argS* | Arginyl-tRNA synthetase (EC 6.1.1.19) |  |  |  |
| SAUSA300_0597 | *-* | Endonuclease III (EC 4.2.99.18) |  |  |  |
| SAUSA300_0598 | *-* | Cobalamin-binding protein | 2.03 |  |  |
| SAUSA300_0599 | *-* | Transporter |  |  |  |
| SAUSA300_0600 | *-* | Hydrolase (HAD superfamily) |  |  |  |
| SAUSA300_0601 | *-* | 3-oxoadipate enol-lactonase (EC 3.1.1.24) |  |  |  |
| SAUSA300_0602 | *-* | Hypothetical exported protein |  |  |  |
| SAUSA300_0603 | *-* | Hypothetical protein |  |  |  |
| SAUSA300_0604 | *-* | Esterase (EC 3.1.1.-) | 2.51 |  |  |
| SAUSA300_0605 | *sarA* | Staphylococcal accessory regulator A |  |  |  |
| SAUSA300_0606 | *-* | Hypothetical membranre spanning protein |  | -2.04 |  |
| SAUSA300_0607 | *-* | Hypothetical protein |  |  |  |
| SAUSA300_0608 | *-* | Hypothetical protein |  |  |  |
| SAUSA300_0609 | *-* | DNA integration/recombination/inversion protein |  |  |  |
| SAUSA300_0610 | *-* | Sodium/proton antiporter protein ShaA |  |  |  |
| SAUSA300_0611 | *-* | Sodium/proton antiporter protein ShaB |  |  |  |
| SAUSA300_0612 | *-* | Sodium/proton antiporter protein shaC |  |  |  |
| SAUSA300_0613 | *-* | Hypothetical protein |  |  |  |
| SAUSA300_0614 | *-* | Sodium/proton antiporter protein ShaE |  |  |  |
| SAUSA300_0615 | *-* | Sodium/proton antiporter protein ShaF |  |  |  |
| SAUSA300_0616 | *-* | Sodium/proton antiporter protein ShaG |  |  |  |
| SAUSA300_0617 | *-* | Na+/H+ antiporter nhaP |  |  |  |
| SAUSA300_0618 | *-* | Manganese-binding protein |  |  |  |
| SAUSA300_0619 | *-* | Manganese transport system membrane protein |  |  |  |
| SAUSA300_0620 | *-* | Manganese transport system ATP-binding protein |  |  |  |
| SAUSA300_0621 | *-* | Iron-dependent repressor |  |  |  |
| SAUSA300_0622 | *-* | Hypothetical membrane spanning protein |  |  |  |
| SAUSA300_0623 | *tagA* | N-acetylglucosaminyldiphosphoundecaprenol N-acetyl-beta-D-mannosaminyltransferase (EC 2.4.1.187) |  |  |  |
| SAUSA300_0624 | *tagH* | Teichoic acid translocation ATP-binding protein tagH |  |  |  |
| SAUSA300_0625 | *tagG* | Teichoic acid translocation permease protein tagG |  |  |  |
| SAUSA300_0626 | *tagB* | Teichoic acid biosynthesis protein B precursor |  |  |  |
| SAUSA300_0627 | *tagX* | TAGX | -2.00 |  |  |
| SAUSA300_0628 | *-* | Glycerol-3-phosphate cytidylyltransferase (EC 2.7.7.39) |  |  |  |
| SAUSA300_0629 | *pbp4* | Hypothetical protein |  |  |  |
| SAUSA300_0630 | *-* | Multidrug resistance ABC transporter ATP-binding and permease protein |  |  |  |
| SAUSA300_0631 | *-* | Nucleoside permease nupC |  |  |  |
| SAUSA300_0632 | *-* | Hypothetical membrane spanning protein |  |  |  |
| SAUSA300_0633 | *fhuA* | Ferrichrome transport ATP-binding protein fhuC |  |  |  |
| SAUSA300_0634 | *fhuB* | Ferrichrome transport system permease protein fhuB |  |  |  |
| SAUSA300_0635 | *fhuG* | Ferrichrome transport system permease protein FhuG |  |  |  |
| SAUSA300_0636 | *-* | Dihydroxyacetone kinase (EC 2.7.1.29) |  | 2.33 | -3.04 |
| SAUSA300_0637 | *-* | Dihydroxyacetone kinase (EC 2.7.1.29) |  | 2.08 | -2.94 |
| SAUSA300_0638 | *-* | Dihydroxyacetone kinase phosphotransfer protein | -2.42 |  | -2.89 |
| SAUSA300_0639 | *-* | Hypothetical protein |  |  |  |
| SAUSA300_0640 | *-* | Hypothetical membrane spanning protein |  |  |  |
| SAUSA300_0641 | *-* | Lipase (EC 3.1.1.3) |  |  |  |
| SAUSA300_0642 | *-* | Hypothetical protein |  |  | 2.69 |
| SAUSA300_0643 | *-* | Acetyltransferase, GNAT family |  |  |  |
| SAUSA300_0644 | *-* | NADH-ubiquinone oxidoreductase 39 kDa subunit (EC 1.6.5.3) / NADH dehydrogenase (EC 1.6.99.3) |  |  |  |
| SAUSA300_0645 | *-* | Two-component response regulator ytsA |  |  |  |
| SAUSA300_0646 | *-* | Hypothetical protein |  |  | 3.33 |
| SAUSA300_0647 | *-* | Bacitracin transport ATP-binding protein ytsC |  |  |  |
| SAUSA300_0648 | *-* | ABC transporter permease protein |  |  |  |
| SAUSA300_0649 | *-* | Putative pit accessory protein |  |  |  |
| SAUSA300_0650 | *-* | Low-affinity inorganic phosphate transporter |  |  |  |
| SAUSA300_0651 | *-* | Secretory antigen precursor SsaA |  |  |  |
| SAUSA300_0652 | *-* | Transporter |  |  |  |
| SAUSA300_0653 | *-* | Transcriptional regulator, AraC family |  |  |  |
| SAUSA300_0654 | *sarX* | Staphylococcal accessory regulator a |  | -2.08 |  |
| SAUSA300_0655 | *-* | Hypothetical cytosolic protein |  |  |  |
| SAUSA300_0656 | *-* | Cupin superfamily protein |  |  |  |
| SAUSA300_0657 | *-* | Hypothetical protein |  |  |  |
| SAUSA300_0658 | *-* | Hydrogen peroxide-inducible genes activator |  |  |  |
| SAUSA300_0659 | *-* | Sugar efflux transporter A |  |  |  |
| SAUSA300_0660 | *-* | Hypothetical protein |  |  |  |
| SAUSA300_0661 | *-* | Hypothetical membrane spanning protein |  |  |  |
| SAUSA300_0662 | *-* | AMINOGLYCOSIDE 6'-N-ACETYLTRANSFERASE IK |  |  |  |
| SAUSA300_0663 | *-* | Hypothetical exported protein |  |  |  |
| SAUSA300_0664 | *-* | Hypothetical protein |  |  |  |
| SAUSA300_0665 | *-* | Ribosomal-protein-alanine acetyltransferase (EC 2.3.1.128) |  |  |  |
| SAUSA300_0666 | *-* | Lysine decarboxylase family |  |  |  |
| SAUSA300_0667 | *-* | EMG2 protein |  |  |  |
| SAUSA300_0668 | *-* | Hypothetical protein |  |  |  |
| SAUSA300_0669 | *-* | Undecaprenyl-diphosphatase (EC 3.6.1.27) (Bacitracin resistance protein) |  |  |  |
| SAUSA300_0670 | *-* | Transport ATP-binding protein CydD |  |  |  |
| SAUSA300_0671 | *-* | Hypothetical protein |  |  |  |
| SAUSA300_0672 | *-* | Transcriptional regulator, MarR family | -2.21 |  |  |
| SAUSA300_0673 | *-* | Low-affinity zinc transport protein |  |  |  |
| SAUSA300_0674 | *-* | Oxidoreductase (EC 1.1.1.-) |  |  |  |
| SAUSA300_0675 | *-* | Hypothetical protein |  |  |  |
| SAUSA300_0676 | *-* | Transporter, Divalent Anion:Sodium Symporter family |  |  |  |
| SAUSA300_0677 | *-* | Deoxyribodipyrimidine photolyase (EC 4.1.99.3) |  |  |  |
| SAUSA300_0678 | *-* | Permease |  |  |  |
| SAUSA300_0679 | *-* | hypothetical DNA-binding protein |  |  |  |
| SAUSA300_0680 | *norA* | Quinolone resistance protein NorA |  |  |  |
| SAUSA300_0681 | *-* | Hypothetical protein |  |  |  |
| SAUSA300_0682 | *ybaK* | Regulatory protein |  |  |  |
| SAUSA300_0683 | *-* | Fructose repressor | -2.02 |  |  |
| SAUSA300_0684 | *fruB* | 1-phosphofructokinase (EC 2.7.1.56) |  |  |  |
| SAUSA300_0685 | *fruA* | PTS system, fructose-specific IIABC component (EC 2.7.1.69) |  |  |  |
| SAUSA300_0686 | *nagA* | N-acetylglucosamine-6-phosphate deacetylase (EC 3.5.1.25) |  |  |  |
| SAUSA300_0687 | *-* | Magnesium and cobalt efflux protein corC |  |  |  |
| SAUSA300_0688 | *-* | 2,5-diketo-D-gluconic acid reductase (EC 1.1.1.274) |  |  |  |
| SAUSA300_0689 | *-* | Undecaprenyl-phosphate beta-N-acetylglucosaminyltransferase (EC 2.4.1.-) |  |  |  |
| SAUSA300_0690 | *saeS* | Sensory transduction protein kinase SaeS (EC 2.7.3.-) |  |  |  |
| SAUSA300_0691 | *saeR* | Two-component response regulator SaeR |  |  |  |
| SAUSA300_0692 | *-* | Hypothetical protein |  |  |  |
| SAUSA300_0693 | *-* | Hypothetical protein |  |  |  |
| SAUSA300_0694 | *-* | Hypothetical protein |  |  |  |
| SAUSA300_0695 | *-* | Queuosine biosynthesis protein QueE |  |  |  |
| SAUSA300_0696 | *-* | 6-pyruvoyl tetrahydropterin synthase (EC 4.2.3.12) |  |  |  |
| SAUSA300_0697 | *-* | Queuosine biosynthesis protein QueC | 2.17 |  | 2.34 |
| SAUSA300_0698 | *pabA* | Anthranilate synthase component II (EC 4.1.3.27) / Para-aminobenzoate synthase glutamine amidotransferase component II (EC 6.3.5.8) |  |  |  |
| SAUSA300_0699 | *-* | Para-aminobenzoate synthetase component I (EC 6.3.5.8) |  |  |  |
| SAUSA300_0700 | *-* | 4-amino-4-deoxychorismate lyase (EC 4.1.3.38) |  |  |  |
| SAUSA300_0701 | *-* | Kinase autophosphorylation inhibitor kipI |  |  |  |
| SAUSA300_0702 | *-* | Regulator of kinase autophosphorylation inhibitor |  |  |  |
| SAUSA300_0703 | *-* | Sulfatase family protein |  |  |  |
| SAUSA300_0704 | *-* | ABC transporter ATP-binding protein Uup |  |  |  |
| SAUSA300_0705 | *recQ* | ATP-dependent DNA helicase recQ (EC 3.6.1.-) |  |  |  |
| SAUSA300_0706 | *-* | Glycine betaine transport ATP-binding protein |  |  |  |
| SAUSA300_0707 | *-* | Glycine betaine transport system permease protein / Glycine betaine-binding protein |  |  |  |
| SAUSA300_0708 | *hisC* | Histidinol-phosphate aminotransferase (EC 2.6.1.9) |  |  |  |
| SAUSA300_0709 | *-* | Hydrolase (HAD superfamily) |  |  |  |
| SAUSA300_0710 | *-* | Hypothetical protein |  |  |  |
| SAUSA300_0711 | *-* | Hypothetical protein |  |  |  |
| SAUSA300_0712 | *-* | Di-/tripeptide transporter |  |  |  |
| SAUSA300_0713 | *folE* | Queuosine biosynthesis protein QueF |  |  |  |
| SAUSA300_0714 | *-* | Transporter, drug/metabolite exporter family |  |  |  |
| SAUSA300_0715 | *nrdI* | NrdI protein |  |  |  |
| SAUSA300_0716 | *-* | Ribonucleoside-diphosphate reductase alpha chain (EC 1.17.4.1) |  |  |  |
| SAUSA300_0717 | *nrdF* | Ribonucleoside-diphosphate reductase beta chain (EC 1.17.4.1) |  |  |  |
| SAUSA300_0718 | *sstA* | Ferric anguibactin transport system permease protein fatD |  |  |  |
| SAUSA300_0719 | *sstB* | Ferric anguibactin transport system permease protein fatC |  |  |  |
| SAUSA300_0720 | *sstC* | Ferric anguibactin transport ATP-binding protein |  |  |  |
| SAUSA300_0721 | *sstD* | Ferric anguibactin-binding protein |  |  |  |
| SAUSA300_0722 | *murB* | UDP-N-acetylenolpyruvoylglucosamine reductase (EC 1.1.1.158) |  |  |  |
| SAUSA300_0723 | *-* | Glutamate-rich protein GrpB |  |  |  |
| SAUSA300_0724 | *-* | Hypothetical protein |  |  |  |
| SAUSA300_0725 | *-* | Hypothetical cytosolic protein |  |  |  |
| SAUSA300_0726 | *-* | Glycerate kinase (EC 2.7.1.31) |  |  |  |
| SAUSA300_0727 | *pepT* | Tripeptidase T (EC 3.4.11.4) |  |  |  |
| SAUSA300_0728 | *-* | Threonine/Serine Exporter |  |  |  |
| SAUSA300_0729 | *-* | Threonine/Serine Exporter |  |  |  |
| SAUSA300_0730 | *-* | Sensory transduction protein kinase (EC 2.7.3.-) |  |  |  |
| SAUSA300_0731 | *-* | Undecaprenyl-phosphate alpha-N-acetylglucosaminephosphotransferase (EC 2.7.8.-) |  |  |  |
| SAUSA300_0732 | *-* | Hypothetical protein |  |  |  |
| SAUSA300_0733 | *-* | DegV family protein |  |  |  |
| SAUSA300_0734 | *-* | COMF operon protein 1 |  |  |  |
| SAUSA300_0735 | *-* | COMF operon protein 3 |  |  |  |
| SAUSA300_0736 | *yfiA* | Ribosome-associated factor Y |  |  |  |
| SAUSA300_0737 | *secA* | Protein translocase subunit SecA |  |  |  |
| SAUSA300_0739 | *-* | Secretory antigen precursor SsaA |  |  |  |
| SAUSA300_0740 | *-* | Hydrolase (HAD superfamily) |  |  |  |
| SAUSA300_0741 | *uvrB* | Excinuclease ABC subunit B |  |  |  |
| SAUSA300_0742 | *uvrA* | Excinuclease ABC subunit A |  |  |  |
| SAUSA300_0743 | *hprK* | HPR(SER) KINASE (EC 2.7.1.-) / PHOSPHATASE (EC 3.1.3.-) |  |  |  |
| SAUSA300_0744 | *lgt* | Prolipoprotein diacylglyceryl transferase (EC 2.4.99.-) |  |  |  |
| SAUSA300_0745 | *-* | O-acetyltransferase (EC 2.3.1.-) |  |  |  |
| SAUSA300_0746 | *-* | Tetratricopeptide repeat family protein |  |  |  |
| SAUSA300_0747 | *trxB* | Thioredoxin reductase (EC 1.8.1.9) |  |  |  |
| SAUSA300_0748 | *-* | ATP-binding protein (contains P-loop) |  |  |  |
| SAUSA300_0749 | *-* | Hypothetical membrane associated protein |  |  |  |
| SAUSA300_0750 | *-* | Hypothetical cytosolic protein |  |  |  |
| SAUSA300_0752 | *clpP* | ATP-dependent endopeptidase clp proteolytic subunit clpP (EC 3.4.21.92) |  |  |  |
| SAUSA300_0753 | *-* | Cell division inhibitor |  |  |  |
| SAUSA300_0754 | *-* | Hypothetical protein |  |  |  |
| SAUSA300_0755 | *-* | Central glycolytic genes regulator |  |  |  |
| SAUSA300_0756 | *gap* | Glyceraldehyde 3-phosphate dehydrogenase (EC 1.2.1.12) |  |  |  |
| SAUSA300_0757 | *pgk* | Phosphoglycerate kinase (EC 2.7.2.3) |  |  |  |
| SAUSA300_0758 | *tpiA* | Triosephosphate isomerase (EC 5.3.1.1) |  |  | 2.15 |
| SAUSA300_0759 | *gpmI* | Phosphoglycerate mutase (EC 5.4.2.1) |  |  |  |
| SAUSA300_0760 | *eno* | Enolase (EC 4.2.1.11) |  |  |  |
| SAUSA300_0761 | *-* | Hypothetical membrane spanning protein |  |  |  |
| SAUSA300_0762 | *secG* | Protein translocase subunit secG |  |  |  |
| SAUSA300_0763 | *est* | Carboxylesterase (EC 3.1.1.1) |  |  |  |
| SAUSA300_0764 | *rnr* | Exoribonuclease II (EC 3.1.13.1) |  |  |  |
| SAUSA300_0765 | *smpB* | SsrA-binding protein |  |  |  |
| SAUSA300_0767 | *-* | Hypothetical protein |  |  |  |
| SAUSA300_0768 | *-* | Hypothetical protein |  |  |  |
| SAUSA300_0769 | *-* | Hypothetical exported protein |  |  |  |
| SAUSA300_0770 | *-* | Hypothetical protein | 2.26 |  |  |
| SAUSA300_0771 | *-* | Acetyltransferase (EC 2.3.1.-) |  |  |  |
| SAUSA300_0772 | *clfA* | Fibronectin-binding protein |  |  |  |
| SAUSA300_0773 | *-* | Staphylocoagulase precursor |  |  |  |
| SAUSA300_0774 | *empbp* | Extracellular matrix binding protein / Fibrinogen-binding protein |  | 2.35 |  |
| SAUSA300_0775 | *-* | extracellular matrix and plasma binding protein |  |  |  |
| SAUSA300_0776 | *nuc* | Thermonuclease (EC 3.1.31.1) |  |  |  |
| SAUSA300_0777 | *-* | Cold shock protein |  |  |  |
| SAUSA300_0778 | *-* | Hypothetical protein |  |  |  |
| SAUSA300_0779 | *-* | Phage protein |  |  |  |
| SAUSA300_0780 | *-* | Hypothetical exported protein |  |  |  |
| SAUSA300_0781 | *-* | Hypothetical protein |  |  |  |
| SAUSA300_0782 | *-* | Hypothetical protein |  |  |  |
| SAUSA300_0783 | *-* | Phosphoglycerate mutase family protein | -4.28 | -3.14 |  |
| SAUSA300_0784 | *-* | Transporter, LysE family |  |  |  |
| SAUSA300_0785 | *-* | Acetyltransferase (EC 2.3.1.-) |  |  |  |
| SAUSA300_0786 | *-* | Organic hydroperoxide resistance protein | 2.36 |  |  |
| SAUSA300_0787 | *aroD* | 3-dehydroquinate dehydratase (EC 4.2.1.10) |  |  |  |
| SAUSA300_0788 | *-* | Nitroreductase family |  |  |  |
| SAUSA300_0789 | *-* | Thioredoxin |  |  |  |
| SAUSA300_0790 | *-* | Arsenate reductase family protein |  |  |  |
| SAUSA300_0791 | *gcvH* | Glycine cleavage system H protein |  |  |  |
| SAUSA300_0792 | *-* | Hypothetical protein | -2.91 |  |  |
| SAUSA300_0793 | *-* | Hypothetical cytosolic protein | -2.24 |  |  |
| SAUSA300_0794 | *-* | DNA primase (bacterial type) and small primase-like proteins |  |  |  |
| SAUSA300_0795 | *-* | Thioredoxin |  |  |  |
| SAUSA300_0796 | *-* | ABC transporter ATP-binding protein |  |  |  |
| SAUSA300_0797 | *-* | ABC transporter permease protein |  |  |  |
| SAUSA300_0798 | *-* | ABC transporter substrate-binding protein |  |  | 2.27 |
| SAUSA300_0799 | *int* | DNA integration/recombination/inversion protein |  |  |  |
| SAUSA300_0800 | *sek* | Enterotoxin |  |  |  |
| SAUSA300_0801 | *seq* | Enterotoxin |  |  |  |
| SAUSA300_0802 | *-* | Zn-dependent alcohol dehydrogenases and related dehydrogenases |  |  |  |
| SAUSA300_0803 | *-* | Phage transcriptional repressor |  |  |  |
| SAUSA300_0804 | *-* | Transcriptional regulator, Cro family |  |  | 2.35 |
| SAUSA300_0805 | *-* | Phage protein |  |  | 2.03 |
| SAUSA300_0806 | *-* | Hypothetical protein |  |  | 2.30 |
| SAUSA300_0807 | *-* | Hypothetical protein |  | -2.17 | 2.18 |
| SAUSA300_0808 | *-* | Hypothetical protein |  |  |  |
| SAUSA300_0809 | *-* | DNA primase |  | -2.12 | 2.42 |
| SAUSA300_0810 | *-* | Hypothetical protein |  | -2.36 | 2.25 |
| SAUSA300_0811 | *-* | Hypothetical protein |  | -2.10 | 2.00 |
| SAUSA300_0812 | *-* | Phage Prohead Protease |  | -2.28 |  |
| SAUSA300_0813 | *-* | Hypothetical protein |  | -2.31 | 2.15 |
| SAUSA300_0814 | *-* | Phage-related protein |  |  |  |
| SAUSA300_0815 | *ear* | Hypothetical protein | -4.16 |  | -2.97 |
| SAUSA300_0816 | *-* | Hypothetical protein |  |  |  |
| SAUSA300_0817 | *-* | Integral membrane protein |  |  |  |
| SAUSA300_0818 | *sufC* | ATP-dependent transporter sufC |  |  |  |
| SAUSA300_0819 | *sufD* | SufD protein |  |  |  |
| SAUSA300_0820 | *sufS* | Cysteine desulfurase (EC 2.8.1.7) / Selenocysteine lyase (EC 4.4.1.16) |  |  |  |
| SAUSA300_0821 | *-* | IscU protein |  |  |  |
| SAUSA300_0822 | *sufB* | ABC transporter-associated protein SufB |  |  |  |
| SAUSA300_0823 | *-* | Hypothetical protein |  |  |  |
| SAUSA300_0824 | *-* | Magnesium and cobalt efflux protein corC |  |  |  |
| SAUSA300_0825 | *-* | Nitropropane dioxygenase / Trans-enoyl-CoA reductase family |  |  |  |
| SAUSA300_0826 | *-* | Hypothetical cytosolic protein |  |  |  |
| SAUSA300_0827 | *-* | Hypothetical membrane spanning protein |  |  |  |
| SAUSA300_0828 | *-* | 5'-nucleotidase (EC 3.1.3.5) |  |  |  |
| SAUSA300_0829 | *lipA* | Lipoic acid synthetase (EC 2.8.-.-) |  |  |  |
| SAUSA300_0830 | *-* | Hypothetical cytosolic protein |  |  |  |
| SAUSA300_0831 | *-* | Hypothetical cytosolic protein |  |  |  |
| SAUSA300_0832 | *-* | Hypothetical cytosolic protein |  |  |  |
| SAUSA300_0833 | *-* | Hydrolase (HAD superfamily) |  |  |  |
| SAUSA300_0834 | *-* | Glyoxylate reductase (NADP+) (EC 1.1.1.79) / Glyoxylate reductase (NAD+) (EC 1.1.1.26) / Hydroxypyruvate reductase (EC 1.1.1.81) |  |  |  |
| SAUSA300_0835 | *dltA* | D-alanine-activating enzyme (EC 6.3.2.-) |  |  |  |
| SAUSA300_0836 | *dltB* | Protein DltB |  |  |  |
| SAUSA300_0837 | *dltC* | D-alanyl carrier protein |  |  |  |
| SAUSA300_0838 | *dltD* | Protein DltD precursor |  |  |  |
| SAUSA300_0839 | *-* | NifU-like protein |  |  |  |
| SAUSA300_0840 | *-* | Hypothetical protein |  |  |  |
| SAUSA300_0841 | *-* | NADH dehydrogenase family |  |  |  |
| SAUSA300_0842 | *-* | Hypothetical protein |  |  |  |
| SAUSA300_0843 | *-* | HesB protein family |  |  |  |
| SAUSA300_0844 | *-* | NADH dehydrogenase family |  |  |  |
| SAUSA300_0845 | *ampA* | Cytosol aminopeptidase (EC 3.4.11.1) |  |  |  |
| SAUSA300_0846 | *-* | transport protein | -2.17 | -5.72 | 2.42 |
| SAUSA300_0847 | *-* | ComA operon protein 2 |  |  |  |
| SAUSA300_0848 | *-* | Thioredoxin reductase/alkyl hydroperoxide reductase |  |  |  |
| SAUSA300_0849 | *mnhG* | Sodium/proton antiporter protein shaG |  |  |  |
| SAUSA300_0850 | *mnhF* | Sodium/proton antiporter protein shaF |  |  |  |
| SAUSA300_0851 | *mnhE* | Sodium/proton antiporter protein shaE |  |  |  |
| SAUSA300_0852 | *mnhD* | Sodium/proton antiporter protein shaD |  |  |  |
| SAUSA300_0853 | *mnhC* | Sodium/proton antiporter protein shaC |  |  |  |
| SAUSA300_0854 | *mnhB* | Sodium/proton antiporter protein shaB |  |  |  |
| SAUSA300_0855 | *mnhA* | Sodium/proton antiporter protein shaA |  |  |  |
| SAUSA300_0856 | *-* | Kinase-associated protein B |  |  |  |
| SAUSA300_0857 | *-* | Peptidyl-prolyl cis-trans isomerase (EC 5.2.1.8) |  |  |  |
| SAUSA300_0858 | *-* | S1-type RNA-binding domain |  |  |  |
| SAUSA300_0859 | *-* | Probable NADH-dependent flavin oxidoreductase yqiG (EC 1.-.-.-) |  |  |  |
| SAUSA300_0860 | *rocD* | Ornithine aminotransferase (EC 2.6.1.13) |  |  |  |
| SAUSA300_0861 | *gudB* | NAD-specific glutamate dehydrogenase (EC 1.4.1.2) |  |  |  |
| SAUSA300_0862 | *glpQ* | Glycerophosphoryl diester phosphodiesterase (EC 3.1.4.46) |  |  |  |
| SAUSA300_0863 | *argH* | Argininosuccinate lyase (EC 4.3.2.1) |  |  |  |
| SAUSA300_0864 | *argG* | Argininosuccinate synthase (EC 6.3.4.5) |  |  |  |
| SAUSA300_0865 | *pgi* | Glucose-6-phosphate isomerase (EC 5.3.1.9) |  |  |  |
| SAUSA300_0866 | *-* | Alkaline phosphatase like protein |  |  |  |
| SAUSA300_0867 | *spsA* | Hypothetical protein |  |  |  |
| SAUSA300_0868 | *spsB* | Signal peptidase I (EC 3.4.21.89) |  |  |  |
| SAUSA300_0869 | *rexB* | ATP-dependent nuclease subunit B |  |  |  |
| SAUSA300_0870 | *rexA* | ATP-dependent nuclease subunit A |  |  |  |
| SAUSA300_0871 | *-* | Fumarylacetoacetate hydrolase family protein |  |  |  |
| SAUSA300_0872 | *-* | Hypothetical protein | 2.09 |  |  |
| SAUSA300_0873 | *cdr* | CoA-disulfide reductase (EC 1.8.1.14) |  |  |  |
| SAUSA300_0874 | *-* | Hydrolase (HAD superfamily) |  |  |  |
| SAUSA300_0875 | *-* | Hypothetical cytosolic protein |  |  |  |
| SAUSA300_0876 | *-* | Acyltransferase family |  |  |  |
| SAUSA300_0877 | *clpB* | ClpB protein | 2.24 |  |  |
| SAUSA300_0878 | *-* | Citrate synthase I repressor | -4.54 |  | -3.13 |
| SAUSA300_0879 | *-* | 2-isopropylmalate synthase (EC 2.3.3.13) |  |  |  |
| SAUSA300_0880 | *-* | SUA5 protein |  |  |  |
| SAUSA300_0881 | *-* | Major Facilitator Superfamily |  |  |  |
| SAUSA300_0882 | *-* | Putative phosphatidylethanolamine-binding protein |  |  |  |
| SAUSA300_0883 | *-* | Outer membrane protein | -2.80 |  | -5.28 |
| SAUSA300_0884 | *-* | Hypothetical protein |  |  |  |
| SAUSA300_0885 | *fabH* | 3-oxoacyl-[acyl-carrier-protein] synthase III (EC 2.3.1.41) |  |  |  |
| SAUSA300_0886 | *fabF* | 3-oxoacyl-[acyl-carrier-protein] synthase (EC 2.3.1.41) |  |  |  |
| SAUSA300_0887 | *oppB* | Oligopeptide transport system permease protein oppB |  |  |  |
| SAUSA300_0888 | *oppC* | Oligopeptide transport system permease protein oppC |  |  |  |
| SAUSA300_0889 | *oppD* | Oligopeptide transport ATP-binding protein oppD |  |  |  |
| SAUSA300_0890 | *oppF* | Oligopeptide transport ATP-binding protein oppF |  |  |  |
| SAUSA300_0891 | *oppA* | Oligopeptide-binding protein oppA |  |  |  |
| SAUSA300_0892 | *oppA* | Oligopeptide-binding protein oppA |  |  |  |
| SAUSA300_0893 | *oppF* | Oligopeptide transport ATP-binding protein oppD |  |  |  |
| SAUSA300_0894 | *oppD* | Oligopeptide transport ATP-binding protein oppF |  |  |  |
| SAUSA300_0895 | *oppB* | Oligopeptide transport system permease protein oppB |  |  |  |
| SAUSA300_0896 | *oppC* | Oligopeptide transport system permease protein oppC |  |  |  |
| SAUSA300_0897 | *trpS* | Tryptophanyl-tRNA synthetase (EC 6.1.1.2) |  |  |  |
| SAUSA300_0898 | *spxA* | Arsenate reductase family protein |  |  |  |
| SAUSA300_0899 | *-* | Negative regulator of genetic competence mecA |  |  |  |
| SAUSA300_0900 | *-* | Putative competence protein/transcription factor |  |  |  |
| SAUSA300_0901 | *-* | Putative competence protein/transcription factor |  |  |  |
| SAUSA300_0902 | *pepF* | Oligoendopeptidase F (EC 3.4.24.-) |  |  |  |
| SAUSA300_0903 | *-* | Hypothetical cytosolic protein |  |  |  |
| SAUSA300_0904 | *-* | Globin Family Protein |  |  |  |
| SAUSA300_0905 | *-* | Organic phosphate binding CYTH family protein |  |  |  |
| SAUSA300_0906 | *-* | Hypothetical protein |  |  |  |
| SAUSA300_0907 | *-* | GTP pyrophosphokinase homolog |  |  |  |
| SAUSA300_0908 | *ppnK* | ATP-NAD kinase (EC 2.7.1.23) |  |  |  |
| SAUSA300_0909 | *-* | Ribosomal large subunit pseudouridine synthase D (EC 4.2.1.70) |  |  |  |
| SAUSA300_0910 | *mgtE* | Mg2+ transporter mgtE |  |  |  |
| SAUSA300_0911 | *-* | Na+/H+ antiporter napA |  |  |  |
| SAUSA300_0912 | *-* | Enoyl-[acyl-carrier protein] reductase (NADPH) (EC 1.3.1.10) |  |  |  |
| SAUSA300_0913 | *-* | Hypothetical membrane spanning protein |  |  |  |
| SAUSA300_0914 | *-* | Sodium/proton-dependent alanine carrier protein |  |  |  |
| SAUSA300_0915 | *-* | Acetyl esterase (EC 3.1.1.-) |  |  |  |
| SAUSA300_0916 | *-* | 2'-5' RNA ligase (EC 6.5.1.-) |  |  |  |
| SAUSA300_0917 | *-* | Permease |  |  |  |
| SAUSA300_0918 | *-* | 1,2-diacylglycerol 3-glucosyltransferase (EC 2.4.1.157) / glucosyldiacylglycerol 6-beta-glucosyltransferase (EC 2.4.1.-) |  |  |  |
| SAUSA300_0919 | *murE* | UDP-N-acetylmuramoylalanyl-D-glutamate--2,6-diaminopimelate ligase (EC 6.3.2.13) |  |  |  |
| SAUSA300_0920 | *-* | Hypothetical protein |  |  |  |
| SAUSA300_0921 | *prfC* | Bacterial Peptide Chain Release Factor 3 (RF-3) |  |  |  |
| SAUSA300_0922 | *-* | Tellurite resistance protein TerC |  |  |  |
| SAUSA300_0923 | *htrA* | Endopeptidase degP (EC 3.4.21.-) |  |  |  |
| SAUSA300_0924 | *-* | Potassium uptake protein ktrB |  |  |  |
| SAUSA300_0925 | *-* | 2',3'-cyclic-nucleotide 2'-phosphodiesterase (EC 3.1.4.16) / 3'-nucleotidase (EC 3.1.3.6) |  |  |  |
| SAUSA300_0928 | *comK* | Competence transcription factor | -10.6 | -3.28 | -3.49 |
| SAUSA300_0929 | *-* | Hypothetical protein | 2.52 |  |  |
| SAUSA300_0930 | *-* | Lipoate-protein ligase A (EC 6.3.2.-) |  |  |  |
| SAUSA300_0931 | *-* | Hypothetical protein |  |  |  |
| SAUSA300_0932 | *-* | Hypothetical protein |  |  |  |
| SAUSA300_0933 | *-* | Hypothetical protein |  |  |  |
| SAUSA300_0934 | *-* | Hypothetical protein |  |  |  |
| SAUSA300_0935 | *-* | Hypothetical protein |  |  |  |
| SAUSA300_0936 | *-* | ABC transporter ATP-binding protein |  |  |  |
| SAUSA300_0937 | *-* | Hypothetical protein |  |  | -2.33 |
| SAUSA300_0938 | *-* | Hypothetical protein |  |  |  |
| SAUSA300_0939 | *-* | Poly(Glycerol-phosphate) alpha-glucosyltransferase (EC 2.4.1.52) |  |  |  |
| SAUSA300_0940 | *-* | Hypothetical membrane spanning protein | 2.33 |  |  |
| SAUSA300_0941 | *-* | Iron(III) dicitrate-binding protein |  |  |  |
| SAUSA300_0942 | *-* | Hypothetical membrane spanning protein |  |  |  |
| SAUSA300_0943 | *-* | Hypothetical protein |  |  |  |
| SAUSA300_0944 | *menA* | 1,4-dihydroxy-2-naphthoate polyprenyltransferase (EC 2.5.1.-) |  |  |  |
| SAUSA300_0945 | *-* | Isochorismate synthase (EC 5.4.4.2) |  |  |  |
| SAUSA300_0946 | *menD* | 2-succinyl-6-hydroxy-2,4-cyclohexadiene-1-carboxylate synthase (EC 2.5.1.64) / 2-oxoglutarate decarboxylase (EC 4.1.1.71) |  |  |  |
| SAUSA300_0947 | *-* | Menaquinone biosynthesis related protein |  |  |  |
| SAUSA300_0948 | *menB* | Naphthoate synthase (EC 4.1.3.36) |  |  |  |
| SAUSA300_0949 | *sspC* | Hypothetical protein | -14.30 |  | -15.39 |
| SAUSA300_0950 | *sspB* | Staphopain (EC 3.4.22.-) | -12.65 |  | -16.51 |
| SAUSA300_0951 | *sspA* | Glutamyl endopeptidase precursor (EC 3.4.21.19) | -23.55 |  | -23.81 |
| SAUSA300_0952 | *-* | Aromatic amino acid aminotransferase (EC 2.6.1.57) / Acetyldiaminopimelate aminotransferase (EC 2.6.1.-) |  |  |  |
| SAUSA300_0953 | *-* | Permease | -2.03 |  |  |
| SAUSA300_0954 | *-* | Transcriptional regulator, MarR family |  |  | 2.36 |
| SAUSA300_0955 | *atl* | Peptidoglycan endo-beta-N-acetylglucosaminidase (EC 3.2.1.-) / N-acetylmuramoyl-L-alanine amidase (EC 3.5.1.28) |  |  |  |
| SAUSA300_0956 | *-* | Acetyltransferase, GNAT family | 2.17 |  |  |
| SAUSA300_0957 | *-* | Transcriptional regulator, LytR family |  |  |  |
| SAUSA300_0958 | *-* | Transcriptional regulator, LytR family |  |  |  |
| SAUSA300_0959 | *fmt* | Beta-lactamase family protein |  |  |  |
| SAUSA300_0960 | *qoxD* | Cytochrome aa3 quinol oxidase polypeptide IV (EC 1.9.3.-) |  |  |  |
| SAUSA300_0961 | *qoxC* | Cytochrome aa3 quinol oxidase polypeptide III (EC 1.9.3.-) |  |  |  |
| SAUSA300_0962 | *qoxB* | Cytochrome aa3 quinol oxidase polypeptide I (EC 1.9.3.-) |  |  |  |
| SAUSA300_0963 | *qoxA* | Cytochrome aa3 quinol oxidase polypeptide II (EC 1.9.3.-) |  |  |  |
| SAUSA300_0964 | *-* | Chitinase (EC 3.2.1.14) |  |  |  |
| SAUSA300_0965 | *folD* | Methylenetetrahydrofolate dehydrogenase (NADP+) (EC 1.5.1.5) / Methenyltetrahydrofolate cyclohydrolase (EC 3.5.4.9) |  |  |  |
| SAUSA300_0966 | *purE* | Phosphoribosylaminoimidazole carboxylase carboxyltransferase subunit (EC 4.1.1.21) |  |  |  |
| SAUSA300_0967 | *purK* | Phosphoribosylaminoimidazole carboxylase NCAIR mutase subunit (EC 4.1.1.21) |  |  |  |
| SAUSA300_0968 | *purC* | Phosphoribosylamidoimidazole-succinocarboxamide synthase (EC 6.3.2.6) |  |  |  |
| SAUSA300_0969 | *purS* | Phosphoribosylformylglycinamidine synthase, purS component (EC 6.3.5.3) |  |  |  |
| SAUSA300_0970 | *purQ* | Phosphoribosylformylglycinamidine synthase (EC 6.3.5.3) |  |  | 2.05 |
| SAUSA300_0971 | *purL* | Phosphoribosylformylglycinamidine synthase (EC 6.3.5.3) |  |  |  |
| SAUSA300_0972 | *purF* | Amidophosphoribosyltransferase (EC 2.4.2.14) |  |  |  |
| SAUSA300_0973 | *purM* | Phosphoribosylformylglycinamidine cyclo-ligase (EC 6.3.3.1) |  |  |  |
| SAUSA300_0974 | *purN* | Phosphoribosylglycinamide formyltransferase (EC 2.1.2.2) |  |  |  |
| SAUSA300_0975 | *purH* | Phosphoribosylaminoimidazolecarboxamide formyltransferase (EC 2.1.2.3) / IMP cyclohydrolase (EC 3.5.4.10) |  |  |  |
| SAUSA300_0976 | *purD* | Phosphoribosylamine--glycine ligase (EC 6.3.4.13) |  |  |  |
| SAUSA300_0977 | *-* | Hydroxymethylpyrimidine transport system permease protein |  |  |  |
| SAUSA300_0978 | *-* | Hydroxymethylpyrimidine transport ATP-binding protein |  |  |  |
| SAUSA300_0979 | *-* | Hydroxymethylpyrimidine transport system permease protein |  |  |  |
| SAUSA300_0980 | *-* | Hypothetical membrane spanning protein |  |  |  |
| SAUSA300_0981 | *-* | Methyltransferase (EC 2.1.1.-) |  |  |  |
| SAUSA300_0982 | *-* | Radical activating enzymes proteins |  |  |  |
| SAUSA300_0983 | *ptsH* | Phosphocarrier protein HPr |  |  |  |
| SAUSA300_0984 | *ptsI* | Phosphoenolpyruvate-protein phosphotransferase (EC 2.7.3.9) |  |  |  |
| SAUSA300_0985 | *-* | Hypothetical protein |  |  |  |
| SAUSA300_0986 | *-* | Cytochrome d ubiquinol oxidase subunit I (EC 1.10.3.-) |  |  |  |
| SAUSA300_0987 | *-* | Cytochrome d ubiquinol oxidase subunit II (EC 1.10.3.-) |  |  |  |
| SAUSA300_0988 | *trkA* | Potassium uptake protein ktrA |  |  |  |
| SAUSA300_0989 | *-* | Zn-dependent hydrolase (EC 3.-.-.-) |  |  |  |
| SAUSA300_0990 | *-* | Putative transcriptional regulator |  |  |  |
| SAUSA300_0991 | ***def*** | Peptide deformylase (EC 3.5.1.88) |  |  |  |
| SAUSA300_0992 | *-* | Hypothetical protein |  |  |  |
| SAUSA300_0993 | *pdhA* | Pyruvate dehydrogenase E1 component alpha subunit (EC 1.2.4.1) |  |  |  |
| SAUSA300_0994 | *pdhB* | Pyruvate dehydrogenase E1 component beta subunit (EC 1.2.4.1) |  |  |  |
| SAUSA300_0995 | *-* | Dihydrolipoamide acetyltransferase component of pyruvate dehydrogenase complex (EC 2.3.1.12) |  |  |  |
| SAUSA300_0996 | *lpdA* | Dihydrolipoamide dehydrogenase (EC 1.8.1.4) |  |  |  |
| SAUSA300_0997 | *-* | Hypothetical cytosolic protein |  |  |  |
| SAUSA300_0998 | *-* | spermidine/putrescine transport operon transcriptional regulator |  |  |  |
| SAUSA300_0999 | *potA* | Spermidine/putrescine transport ATP-binding protein potA |  |  |  |
| SAUSA300_1000 | *potB* | Spermidine/putrescine transport system permease protein PotB |  |  |  |
| SAUSA300_1001 | *potC* | Spermidine/putrescine transport system permease protein PotC |  |  |  |
| SAUSA300_1002 | *potD* | Spermidine/putrescine-binding protein |  |  |  |
| SAUSA300_1003 | *-* | Hypothetical protein |  |  |  |
| SAUSA300_1004 | *-* | Hypothetical protein |  |  |  |
| SAUSA300_1005 | *-* | Manganese transport protein MntH |  |  | -2.21 |
| SAUSA300_1006 | *-* | Hypothetical cytosolic protein |  |  |  |
| SAUSA300_1007 | *-* | Myo-inositol-1(or 4)-monophosphatase (EC 3.1.3.25) |  |  |  |
| SAUSA300_1008 | *-* | Hypothetical protein |  |  |  |
| SAUSA300_1009 | *typA* | GTP-binding protein TypA/BipA |  |  |  |
| SAUSA300_1010 | *-* | Hypothetical protein |  |  |  |
| SAUSA300_1011 | *-* | Hypothetical protein |  |  |  |
| SAUSA300_1012 | *-* | Hypothetical protein |  |  |  |
| SAUSA300_1013 | *-* | Cell division protein FtsW |  |  |  |
| SAUSA300_1014 | *pyc* | Pyruvate carboxylase (EC 6.4.1.1) |  |  |  |
| SAUSA300_1015 | *ctaA* | Heme O monooxygenase (EC 1.-.-.-) |  |  |  |
| SAUSA300_1016 | *cyoE* | Protoheme IX farnesyltransferase (EC 2.5.1.-) |  |  |  |
| SAUSA300_1017 | *-* | Hypothetical membrane spanning protein |  |  |  |
| SAUSA300_1018 | *-* | Hypothetical protein |  |  |  |
| SAUSA300_1019 | *-* | Hypothetical protein |  |  |  |
| SAUSA300_1020 | *-* | Glycerophosphoryl diester phosphodiesterase (EC 3.1.4.46) |  |  |  |
| SAUSA300_1021 | *-* | Hypothetical cytosolic protein |  |  |  |
| SAUSA300_1022 | *-* | Hypothetical protein |  |  |  |
| SAUSA300_1023 | *-* | Methyltransferase (EC 2.1.1.-) |  |  |  |
| SAUSA300_1024 | *coaD* | Phosphopantetheine adenylyltransferase (EC 2.7.7.3) |  |  |  |
| SAUSA300_1025 | *-* | Hypothetical cytosolic protein |  |  |  |
| SAUSA300_1026 | *-* | Hypothetical protein |  |  |  |
| SAUSA300_1027 | *rpmF* | LSU ribosomal protein L32P |  |  |  |
| SAUSA300_1028 | *isdB* | Iron transport associated domain-containing protein |  |  |  |
| SAUSA300_1029 | *isdA* | Iron transport associated domain-containing protein |  |  |  |
| SAUSA300_1030 | *isdC* | Iron transport associated domain-containing protein |  |  |  |
| SAUSA300_1031 | *isdD* | Hypothetical membrane associated protein |  |  |  |
| SAUSA300_1032 | *isdE* | Ferrichrome-binding protein |  |  |  |
| SAUSA300_1033 | *isdF* | Transporter |  |  |  |
| SAUSA300_1034 | *srtB* | Sortase B family protein |  |  |  |
| SAUSA300_1035 | *isdG* | Heme-degrading monooxygenase IsdG |  |  |  |
| SAUSA300_1036 | *-* | 23S rRNA methyltransferase (EC 2.1.1.-) |  |  |  |
| SAUSA300_1037 | *pheS* | Phenylalanyl-tRNA synthetase alpha chain (EC 6.1.1.20) |  |  |  |
| SAUSA300_1038 | *pheT* | Phenylalanyl-tRNA synthetase beta chain (EC 6.1.1.20) |  |  |  |
| SAUSA300_1039 | *rnhC* | Ribonuclease HIII (EC 3.1.26.4) |  |  |  |
| SAUSA300_1040 | *-* | Hypothetical protein |  |  |  |
| SAUSA300_1041 | *-* | Colicin V production protein |  |  |  |
| SAUSA300_1042 | *-* | DNA Polymerase X family (EC 2.7.7.7) |  |  |  |
| SAUSA300_1043 | *mutS2* | DNA mismatch repair protein mutS |  |  |  |
| SAUSA300_1044 | *trx* | Thioredoxin |  |  |  |
| SAUSA300_1045 | *uvrC* | Excinuclease ABC subunit C |  |  |  |
| SAUSA300_1046 | *sdhC* | Succinate dehydrogenase cytochrome b558 subunit (EC 1.3.99.1) |  |  |  |
| SAUSA300_1047 | *sdhA* | Succinate dehydrogenase flavoprotein subunit (EC 1.3.99.1) |  |  |  |
| SAUSA300_1048 | *sdhB* | Succinate dehydrogenase iron-sulfur protein (EC 1.3.99.1) |  |  |  |
| SAUSA300_1049 | *murI* | Glutamate racemase (EC 5.1.1.3) |  |  |  |
| SAUSA300_1050 | *-* | Xanthosine triphosphate pyrophosphatase (EC 3.6.1.-) |  |  |  |
| SAUSA300_1051 | *-* | putative phosphoesterase |  |  |  |
| SAUSA300_1052 | *-* | Fibrinogen-binding protein precursor | 2.05 |  | 5.38 |
| SAUSA300_1053 | *-* | Hypothetical protein | -2.00 |  |  |
| SAUSA300_1054 | *-* | Hypothetical membrane spanning protein |  |  |  |
| SAUSA300_1055 | *efb* | Fibrinogen-binding protein precursor |  |  |  |
| SAUSA300_1056 | *-* | Hypothetical protein |  |  |  |
| SAUSA300_1057 | *-* | Hypothetical protein |  |  |  |
| SAUSA300_1058 | *hla* | Alpha-hemolysin | -12.38 |  | -4.53 |
| SAUSA300_1059 | *-* | Exotoxin |  |  |  |
| SAUSA300_1060 | *-* | Exotoxin |  |  | 2.60 |
| SAUSA300_1061 | *-* | Exotoxin | 2.01 |  | 3.49 |
| SAUSA300_1062 | *argF* | Ornithine carbamoyltransferase | 5.09 |  |  |
| SAUSA300_1063 | *arcC* | Carbamate kinase (EC 2.7.2.2) |  |  |  |
| SAUSA300_1064 | *-* | Arginine/ornithine antiporter |  |  |  |
| SAUSA300_1065 | *-* | Transporter |  |  |  |
| SAUSA300_1067 | *-* | Antibacterial protein 3 | -5.82 |  | -336.62 |
| SAUSA300_1068 | *-* | Phenol soluble modulin beta 1 | -6.22 |  | -259.74 |
| SAUSA300_1069 | *-* | Hydrolase (HAD superfamily) |  |  |  |
| SAUSA300_1070 | *-* | Acetyltransferase (EC 2.3.1.-) |  |  |  |
| SAUSA300_1071 | *-* | Hypothetical membrane spanning protein |  |  |  |
| SAUSA300_1072 | *mraZ* | Cell division protein MraZ |  |  |  |
| SAUSA300_1073 | *mraW* | S-adenosyl-methyltransferase MraW (EC 2.1.1.-) |  |  |  |
| SAUSA300_1074 | *ftsL* | Cell division protein ftsL |  |  |  |
| SAUSA300_1075 | *pbpA* | Division specific D,D-transpeptidase / Cell division protein FtsI |  |  |  |
| SAUSA300_1076 | *mraY* | Phospho-N-acetylmuramoyl-pentapeptide-transferase (EC 2.7.8.13) |  |  |  |
| SAUSA300_1077 | *murD* | UDP-N-acetylmuramoylalanine--D-glutamate ligase (EC 6.3.2.9) |  |  |  |
| SAUSA300_1078 | *divIB* | Hypothetical protein |  |  |  |
| SAUSA300_1079 | *ftsA* | Cell division protein ftsA |  |  |  |
| SAUSA300_1080 | *ftsZ* | Cell division protein ftsZ |  |  |  |
| SAUSA300_1081 | *-* | Hypothetical cytosolic protein |  |  |  |
| SAUSA300_1082 | *-* | Pyridoxal-5'-phosphate family protein |  |  |  |
| SAUSA300_1083 | *-* | Hypothetical cytosolic protein |  |  |  |
| SAUSA300_1084 | *-* | Integral membrane protein |  |  |  |
| SAUSA300_1085 | *-* | RNA binding protein |  |  |  |
| SAUSA300_1086 | *-* | Cell division initiation protein DivIVA |  |  |  |
| SAUSA300_1087 | *ileS* | Isoleucyl-tRNA synthetase (EC 6.1.1.5) |  |  |  |
| SAUSA300_1088 | *-* | Glyoxalase family protein |  |  |  |
| SAUSA300_1089 | *lspA* | Lipoprotein signal peptidase (EC 3.4.23.36) |  |  |  |
| SAUSA300_1090 | *-* | Ribosomal large subunit pseudouridine synthase D (EC 4.2.1.70) |  |  |  |
| SAUSA300_1091 | *pyrR* | Uracil phosphoribosyltransferase (EC 2.4.2.9) / Pyrimidine operon regulatory protein PyrR |  |  |  |
| SAUSA300_1092 | *pyrP* | Uracil permease |  |  |  |
| SAUSA300_1093 | *pyrB* | Aspartate carbamoyltransferase (EC 2.1.3.2) |  |  |  |
| SAUSA300_1094 | *pyrC* | Dihydroorotase (EC 3.5.2.3) |  |  |  |
| SAUSA300_1095 | *carA* | Carbamoyl-phosphate synthase small chain (EC 6.3.5.5) |  |  |  |
| SAUSA300_1096 | *carB* | Carbamoyl-phosphate synthase large chain (EC 6.3.5.5) |  |  |  |
| SAUSA300_1097 | *pyrF* | Orotidine 5'-phosphate decarboxylase (EC 4.1.1.23) |  |  |  |
| SAUSA300_1098 | *pyrE* | Orotate phosphoribosyltransferase (EC 2.4.2.10) |  |  |  |
| SAUSA300_1099 | *-* | Hypothetical protein |  |  |  |
| SAUSA300_1100 | *-* | Glyoxalase family protein |  |  |  |
| SAUSA300_1101 | *-* | Fibronectin-binding protein / Fibrinogen-binding protein |  |  |  |
| SAUSA300_1102 | *gmk* | Guanylate kinase (EC 2.7.4.8) |  |  |  |
| SAUSA300_1103 | *rpoZ* | DNA-directed RNA polymerase omega chain (EC 2.7.7.6) |  |  |  |
| SAUSA300_1104 | *coaBC* | Phosphopantothenoylcysteine decarboxylase (EC 4.1.1.36) / Phosphopantothenate--cysteine ligase (EC 6.3.2.5) |  |  |  |
| SAUSA300_1105 | *priA* | Primosomal protein N' |  |  |  |
| SAUSA300_1106 | *-* | Hypothetical protein |  |  |  |
| SAUSA300_1107 | *-* | Hypothetical protein |  |  |  |
| SAUSA300_1108 | *def* | Peptide deformylase (EC 3.5.1.88) |  |  |  |
| SAUSA300_1109 | *fmt* | Methionyl-tRNA formyltransferase (EC 2.1.2.9) |  |  |  |
| SAUSA300_1110 | *sun* | 16S rRNA m(5)C 967 methyltransferase (EC 2.1.1.-) |  |  |  |
| SAUSA300_1111 | *-* | Radical SAM family enzyme |  |  |  |
| SAUSA300_1112 | *-* | Protein phosphatase 2C (EC 3.1.3.16) |  |  |  |
| SAUSA300_1113 | *pknB* | Serine/threonine protein kinase (EC 2.7.11.1) |  |  |  |
| SAUSA300_1114 | *rsgA* | GTPase (EC 3.6.1.-) |  |  |  |
| SAUSA300_1115 | *rpe* | Ribulose-phosphate 3-epimerase (EC 5.1.3.1) |  |  |  |
| SAUSA300_1116 | *-* | Thiamin pyrophosphokinase (EC 2.7.6.2) |  |  |  |
| SAUSA300_1117 | *rpmB* | LSU ribosomal protein L28P |  |  |  |
| SAUSA300_1118 | *-* | General stress protein, Gls24 family |  |  |  |
| SAUSA300_1119 | *-* | Dihydroxyacetone kinase family protein |  |  |  |
| SAUSA300_1120 | *recG* | ATP-dependent DNA helicase recG (EC 3.6.1.-) |  |  |  |
| SAUSA300_1121 | *-* | PaaI family protein, possible transcriptional regulator |  |  |  |
| SAUSA300_1122 | *plsX* | Fatty acid/phospholipid synthesis protein PlsX |  |  |  |
| SAUSA300_1123 | *fabD* | Malonyl-CoA-[acyl-carrier-protein] transacylase (EC 2.3.1.39) |  |  |  |
| SAUSA300_1124 | *fabG* | 3-oxoacyl-[acyl-carrier protein] reductase (EC 1.1.1.100) |  |  |  |
| SAUSA300_1125 | *acpP* | Acyl carrier protein |  |  |  |
| SAUSA300_1126 | *rnc* | Ribonuclease III (EC 3.1.26.3) |  |  |  |
| SAUSA300_1127 | *smc* | Chromosome partition protein Smc |  |  |  |
| SAUSA300_1128 | *ftsY* | Cell division protein FtsY |  |  |  |
| SAUSA300_1129 | *-* | Signal recognition particle associated protein |  |  |  |
| SAUSA300_1130 | *ffh* | SIGNAL RECOGNITION PARTICLE, SUBUNIT FFH/SRP54 |  |  |  |
| SAUSA300_1131 | *rpsP* | SSU ribosomal protein S16P |  |  |  |
| SAUSA300_1132 | *rimM* | 16S rRNA processing protein RimM |  |  |  |
| SAUSA300_1133 | *trmD* | tRNA (Guanine-N(1)-)-methyltransferase (EC 2.1.1.31) |  |  |  |
| SAUSA300_1134 | *rplS* | LSU ribosomal protein L19P |  |  | 2.63 |
| SAUSA300_1135 | *-* | Integral membrane protein | -2.32 |  |  |
| SAUSA300_1136 | *rbgA* | GTP-binding protein |  |  |  |
| SAUSA300_1137 | *rnhB* | Ribonuclease HII (EC 3.1.26.4) |  |  |  |
| SAUSA300_1138 | *sucC* | Succinyl-CoA synthetase beta chain (EC 6.2.1.5) |  |  |  |
| SAUSA300_1139 | *sucD* | Succinyl-CoA synthetase alpha chain (EC 6.2.1.5) |  |  |  |
| SAUSA300_1140 | *lytN* | Cell wall hydrolase LytN |  |  |  |
| SAUSA300_1141 | *-* | UDP-N-acetylmuramoylheptapeptide-glycine L-seryltransferase (EC 2.3.2.-) |  |  |  |
| SAUSA300_1142 | *dprA* | DNA processing protein |  |  |  |
| SAUSA300_1143 | *topA* | DNA topoisomerase I (EC 5.99.1.2) |  |  |  |
| SAUSA300_1144 | *gid* | Glucose inhibited division protein A |  |  |  |
| SAUSA300_1145 | *xerC* | Integrase/recombinase (XerC/CodV family) |  |  |  |
| SAUSA300_1146 | *hslV* | ATP-dependent endopeptidase hsl proteolytic subunit hslV (EC 3.4.25.-) |  |  |  |
| SAUSA300_1147 | *hslU* | ATP-dependent endopeptidase hsl ATP-binding subunit hslU |  |  |  |
| SAUSA300_1148 | *codY* | Transcription pleiotropic repressor codY |  |  |  |
| SAUSA300_1149 | *rpsB* | SSU ribosomal protein S2P |  |  |  |
| SAUSA300_1150 | *tsf* | Protein Translation Elongation Factor Ts (EF-Ts) |  |  |  |
| SAUSA300_1151 | *pyrH* | Uridylate kinase (EC 2.7.4.-) |  |  |  |
| SAUSA300_1152 | *frr* | Ribosome Recycling Factor (RRF) |  |  |  |
| SAUSA300_1153 | *uppS* | Undecaprenyl pyrophosphate synthetase (EC 2.5.1.31) |  |  |  |
| SAUSA300_1154 | *cdsA* | Phosphatidate cytidylyltransferase (EC 2.7.7.41) |  |  |  |
| SAUSA300_1155 | *-* | Membrane endopeptidase, M50 family |  |  |  |
| SAUSA300_1156 | *proS* | Prolyl-tRNA synthetase (EC 6.1.1.15) |  |  |  |
| SAUSA300_1157 | *polC* | DNA polymerase III alpha subunit (EC 2.7.7.7) |  |  |  |
| SAUSA300_1158 | *-* | Hypothetical cytosolic protein |  |  |  |
| SAUSA300_1159 | *nusA* | N utilization substance protein A |  |  |  |
| SAUSA300_1160 | *-* | Hypothetical cytosolic protein |  |  |  |
| SAUSA300_1161 | *-* | LSU ribosomal protein L7AE |  |  |  |
| SAUSA300_1162 | *infB* | Bacterial Protein Translation Initiation Factor 2 (IF-2) |  |  |  |
| SAUSA300_1163 | *rbfA* | Ribosome-binding factor A |  |  |  |
| SAUSA300_1164 | *truB* | tRNA pseudouridine synthase B (EC 4.2.1.70) |  |  |  |
| SAUSA300_1165 | *ribF* | Riboflavin kinase (EC 2.7.1.26) / FMN adenylyltransferase (EC 2.7.7.2) |  |  |  |
| SAUSA300_1166 | *rpsO* | SSU ribosomal protein S15P |  |  |  |
| SAUSA300_1167 | *pnpA* | Polyribonucleotide nucleotidyltransferase (EC 2.7.7.8) / Polynucleotide adenylyltransferase (EC 2.7.7.19) |  |  |  |
| SAUSA300_1168 | *-* | Zn-dependent hydrolase (EC 3.-.-.-) |  |  |  |
| SAUSA300_1169 | *ftsK* | Cell division protein FtsK |  |  |  |
| SAUSA300_1170 | *-* | Transcriptional regulator, GntR family |  |  |  |
| SAUSA300_1171 | *-* | Non-proteolytic protein, peptidase family M16 |  |  |  |
| SAUSA300_1172 | *-* | Peptidase, M16 family |  |  |  |
| SAUSA300_1173 | *-* | Acetoacetyl-CoA reductase (EC 1.1.1.36) |  |  |  |
| SAUSA300_1174 | *-* | Protein with ACT domain |  |  |  |
| SAUSA300_1175 | *-* | Transcriptional regulator |  |  |  |
| SAUSA300_1176 | *pgsA* | CDP-diacylglycerol--glycerol-3-phosphate 3-phosphatidyltransferase (EC 2.7.8.5) |  |  |  |
| SAUSA300_1177 | *cinA* | Colligrin |  |  |  |
| SAUSA300_1178 | *recA* | RecA protein |  |  |  |
| SAUSA300_1179 | *-* | Hydrolase (HAD superfamily) |  |  |  |
| SAUSA300_1180 | *-* | Hypothetical protein |  |  |  |
| SAUSA300_1181 | *-* | Hypothetical protein |  |  |  |
| SAUSA300_1182 | *-* | 2-oxoacid ferredoxin oxidoreductase, alpha subunit (EC 1.2.7.-) | 3.06 |  |  |
| SAUSA300_1183 | *-* | 2-oxoacid ferredoxin oxidoreductase, beta subunit (EC 1.2.7.-) | 2.94 |  |  |
| SAUSA300_1184 | *-* | Hypothetical protein |  |  |  |
| SAUSA300_1185 | *miaB* | tRNA 2-methylthioadenosine synthase |  |  |  |
| SAUSA300_1186 | *-* | Hypothetical protein |  |  |  |
| SAUSA300_1187 | *-* | Hydroxyethylthiazole permease |  |  |  |
| SAUSA300_1188 | *mutS* | DNA mismatch repair protein MutS |  |  |  |
| SAUSA300_1189 | *mutL* | DNA mismatch repair protein MutL |  |  |  |
| SAUSA300_1190 | *glpP* | Glycerol uptake operon antiterminator regulatory protein |  |  |  |
| SAUSA300_1191 | *glpF* | Glycerol uptake facilitator protein |  |  |  |
| SAUSA300_1192 | *glpK* | Glycerol kinase (EC 2.7.1.30) |  | 2.37 |  |
| SAUSA300_1193 | *glpD* | Glycerol-3-phosphate dehydrogenase (EC 1.1.99.5) |  |  |  |
| SAUSA300_1194 | *-* | Lysophospholipase L2 (EC 3.1.1.5) |  |  |  |
| SAUSA300_1195 | *miaA* | tRNA delta(2)-isopentenylpyrophosphate transferase (EC 2.5.1.8) |  |  |  |
| SAUSA300_1196 | *hfq* | RNA-binding protein, Hfq family |  |  |  |
| SAUSA300_1197 | *-* | Glutathione peroxidase (EC 1.11.1.9) |  |  |  |
| SAUSA300_1198 | *-* | GTP-binding protein hflX |  |  |  |
| SAUSA300_1199 | *-* | Aluminum resistance protein |  |  |  |
| SAUSA300_1200 | *glnR* | Transcriptional regulator, MerR family |  |  |  |
| SAUSA300_1201 | *glnA* | Glutamine synthetase (EC 6.3.1.2) |  |  |  |
| SAUSA300_1202 | *-* | Hypothetical protein |  |  |  |
| SAUSA300_1203 | *-* | Hypothetical protein | 2.08 |  |  |
| SAUSA300_1204 | *-* | Hypothetical protein |  |  | 2.15 |
| SAUSA300_1205 | *-* | Hypothetical protein |  |  |  |
| SAUSA300_1206 | *-* | Hypothetical protein |  |  |  |
| SAUSA300_1207 | *-* | Phage protein |  |  |  |
| SAUSA300_1208 | *-* | Hypothetical protein |  |  |  |
| SAUSA300_1209 | *-* | Phage protein |  |  |  |
| SAUSA300_1210 | *-* | Phage protein |  |  |  |
| SAUSA300_1211 | *-* | Hypothetical protein |  |  |  |
| SAUSA300_1212 | *-* | Phage protein |  |  |  |
| SAUSA300_1213 | *-* | Hypothetical protein |  |  |  |
| SAUSA300_1214 | *-* | Hypothetical protein | -3.66 |  | -5.43 |
| SAUSA300_1215 | *-* | Hypothetical protein |  |  |  |
| SAUSA300_1216 | *-* | Cardiolipin synthetase (EC 2.7.8.-) |  |  |  |
| SAUSA300_1217 | *-* | ABC transporter ATP-binding protein |  |  |  |
| SAUSA300_1218 | *-* | ABC transporter permease protein |  |  |  |
| SAUSA300_1219 | *-* | Sensory Transduction Histidine Kinase (EC 2.7.3.-) |  |  |  |
| SAUSA300_1220 | *-* | Two-component response regulator |  |  |  |
| SAUSA300_1221 | *-* | Hypothetical protein |  |  |  |
| SAUSA300_1222 | *nuc* | Thermonuclease (EC 3.1.31.1) |  |  |  |
| SAUSA300_1223 | *-* | Hypothetical protein |  |  |  |
| SAUSA300_1224 | *-* | Hypothetical protein | -2.84 |  |  |
| SAUSA300_1225 | *-* | Aspartokinase (EC 2.7.2.4) |  |  |  |
| SAUSA300_1226 | *-* | Homoserine dehydrogenase (EC 1.1.1.3) |  |  |  |
| SAUSA300_1227 | *thrC* | Threonine synthase (EC 4.2.3.1) |  |  |  |
| SAUSA300_1228 | *thrB* | Homoserine kinase (EC 2.7.1.39) |  |  |  |
| SAUSA300_1229 | *-* | Hydrolase (HAD superfamily) |  |  |  |
| SAUSA300_1230 | *-* | Hypothetical protein |  |  |  |
| SAUSA300_1231 | *-* | Lysine-specific permease |  |  |  |
| SAUSA300_1232 | *-* | Catalase (EC 1.11.1.6) |  |  |  |
| SAUSA300_1233 | *rpmG* | Lsu ribosomal protein L33P |  |  |  |
| SAUSA300_1234 | *rpsN* | Ssu ribosomal protein S14P |  |  |  |
| SAUSA300_1235 | *guaC* | GMP reductase (EC 1.7.1.7) |  |  | 2.17 |
| SAUSA300_1236 | *-* | Hypothetical exported protein |  |  |  |
| SAUSA300_1237 | *lexA* | LexA repressor (EC 3.4.21.88) |  |  |  |
| SAUSA300_1238 | *-* | Hypothetical cytosolic protein |  |  |  |
| SAUSA300_1239 | *tkt* | Transketolase (EC 2.2.1.1) |  |  |  |
| SAUSA300_1240 | *-* | Hypothetical exported protein |  |  |  |
| SAUSA300_1241 | *-* | CcdC protein |  |  |  |
| SAUSA300_1242 | *sbcD* | Exonuclease sbcD (EC 3.1.11.-) |  |  |  |
| SAUSA300_1243 | *sbcC* | Exonuclease SbcC (EC 3.1.11.-) |  |  |  |
| SAUSA300_1244 | *mscL* | Large-conductance mechanosensitive channel |  |  |  |
| SAUSA300_1245 | *opuD* | Glycine betaine transporter |  | -2.19 |  |
| SAUSA300_1246 | *acnA* | Aconitate hydratase (EC 4.2.1.3) |  |  |  |
| SAUSA300_1247 | *-* | Thioesterase (EC 3.1.2.-) |  |  |  |
| SAUSA300_1248 | *-* | Hypothetical cytosolic protein | -2.18 |  | -2.18 |
| SAUSA300_1249 | *-* | Hypothetical membrane spanning protein |  |  |  |
| SAUSA300_1250 | *parE* | Topoisomerase IV subunit B (EC 5.99.1.-) |  |  |  |
| SAUSA300_1251 | *parC* | Topoisomerase IV subunit A (EC 5.99.1.-) |  |  |  |
| SAUSA300_1252 | *-* | Na(+)-linked D-alanine glycine permease |  |  |  |
| SAUSA300_1253 | *glcT* | Transcription antiterminator, BglG family |  |  |  |
| SAUSA300_1254 | *-* | Hypothetical membrane spanning protein |  |  |  |
| SAUSA300_1255 | *fmtC* | Lysyltransferase (EC 2.3.2.3) |  |  |  |
| SAUSA300_1256 | *msrA* | Peptide methionine sulfoxide reductase MsrA (EC 1.8.4.11) |  |  |  |
| SAUSA300_1257 | *msrR* | Transcriptional regulator, LytR family |  |  | -2.03 |
| SAUSA300_1258 | *-* | 4-oxalocrotonate tautomerase (EC 5.3.2.-) |  |  |  |
| SAUSA300_1259 | *-* | ImpB/MucB/SamB family protein |  |  |  |
| SAUSA300_1260 | *-* | Arogenate dehydrogenase (EC 1.3.1.43) / Prephenate dehydrogenase (EC 1.3.1.12) |  |  |  |
| SAUSA300_1261 | *-* | Deblocking aminopeptidase (EC 3.4.11.-) |  |  |  |
| SAUSA300_1262 | *trpE* | Anthranilate synthase component I (EC 4.1.3.27) |  |  |  |
| SAUSA300_1263 | *trpG* | Anthranilate synthase component II (EC 4.1.3.27) / Para-aminobenzoate synthase glutamine amidotransferase component II (EC 6.3.5.8) |  |  |  |
| SAUSA300_1264 | *trpD* | Anthranilate phosphoribosyltransferase (EC 2.4.2.18) |  |  |  |
| SAUSA300_1265 | *trpC* | Indole-3-glycerol phosphate synthase (EC 4.1.1.48) |  | 2.02 |  |
| SAUSA300_1266 | *trpF* | N-(5'-phosphoribosyl)anthranilate isomerase (EC 5.3.1.24) |  |  |  |
| SAUSA300_1267 | *trpB* | Tryptophan synthase beta chain (EC 4.2.1.20) |  |  |  |
| SAUSA300_1268 | *trpA* | Tryptophan synthase alpha chain (EC 4.2.1.20) |  |  |  |
| SAUSA300_1269 | *femA* | Hypothetical protein |  |  |  |
| SAUSA300_1270 | *femB* | UDP-N-acetylmuramoylpentapeptide-triglycine glycyltransferase (EC 2.3.2.-) |  |  |  |
| SAUSA300_1271 | *-* | Hydrolase (HAD superfamily) |  |  |  |
| SAUSA300_1272 | *-* | Transcriptional regulator |  |  |  |
| SAUSA300_1273 | *opp-2F* | Hypothetical protein |  |  |  |
| SAUSA300_1274 | *-* | Nickel transport ATP-binding protein NikE |  |  |  |
| SAUSA300_1275 | *-* | Nickel transport system permease protein nikC |  |  |  |
| SAUSA300_1276 | *opp-2B* | Nickel transport system permease protein nikB | -2.10 |  | -2.12 |
| SAUSA300_1277 | *-* | Permease |  |  |  |
| SAUSA300_1278 | *pepF* | Oligoendopeptidase F (EC 3.4.24.-) |  |  |  |
| SAUSA300_1279 | *phoU* | Phosphate transport system protein PhoU |  |  |  |
| SAUSA300_1280 | *pstB* | Phosphate transport ATP-binding protein PstB |  |  |  |
| SAUSA300_1281 | *pstA* | Phosphate transport system permease protein PstA |  |  |  |
| SAUSA300_1282 | *pstC* | Phosphate transport system permease protein PstC |  |  |  |
| SAUSA300_1283 | *pstS* | Phosphate-binding protein |  |  |  |
| SAUSA300_1284 | *-* | S1 RNA binding domain |  |  |  |
| SAUSA300_1285 | *-* | ABC transporter ATP-binding protein |  |  |  |
| SAUSA300_1286 | *-* | Aspartokinase (EC 2.7.2.4) |  |  |  |
| SAUSA300_1287 | *asd* | Aspartate-semialdehyde dehydrogenase (EC 1.2.1.11) |  |  |  |
| SAUSA300_1288 | *dapA* | Dihydrodipicolinate synthase (EC 4.2.1.52) |  |  |  |
| SAUSA300_1289 | *dapB* | Dihydrodipicolinate reductase (EC 1.3.1.26) |  |  |  |
| SAUSA300_1290 | *dapD* | Tetrahydrodipicolinate N-acetyltransferase (EC 2.3.1.89) |  |  |  |
| SAUSA300_1291 | *-* | Putative N-acetyldiaminopimelate deacetylase (EC 3.5.1.47) |  |  |  |
| SAUSA300_1292 | *alr2* | Alanine racemase (EC 5.1.1.1) |  |  |  |
| SAUSA300_1293 | *lysA* | Diaminopimelate decarboxylase (EC 4.1.1.20) |  |  |  |
| SAUSA300_1294 | *-* | Hypothetical protein |  |  |  |
| SAUSA300_1295 | *-* | Cold shock protein |  |  |  |
| SAUSA300_1296 | *-* | Putative DNA binding protein |  |  |  |
| SAUSA300_1297 | *-* | Acylphosphatase (EC 3.6.1.7) |  |  |  |
| SAUSA300_1298 | *-* | XpaC protein |  |  |  |
| SAUSA300_1299 | *-* | Tellurite resistance protein |  |  |  |
| SAUSA300_1300 | *brnQ* | Branched-chain amino acid transport system carrier protein |  |  |  |
| SAUSA300_1301 | *-* | von Willebrand factor type A domain protein |  |  |  |
| SAUSA300_1302 | *-* | Hypothetical ATPase |  |  |  |
| SAUSA300_1303 | *-* | Hypothetical protein |  |  |  |
| SAUSA300_1304 | *-* | Glyoxalase family protein |  |  |  |
| SAUSA300_1305 | *sucB* | Dihydrolipoamide succinyltransferase component (E2) of 2-oxoglutarate dehydrogenase complex (EC 2.3.1.61) | -2.82 |  |  |
| SAUSA300_1306 | *sucA* | 2-oxoglutarate dehydrogenase E1 component (EC 1.2.4.2) | -3.76 |  | -2.40 |
| SAUSA300_1307 | *arlS* | Two-component sensor kinase arlS (EC 2.7.3.-) |  |  |  |
| SAUSA300_1308 | *arlR* | Two-component response regulator arlR |  |  |  |
| SAUSA300_1309 | *-* | Transposase |  |  |  |
| SAUSA300_1310 | *-* | Membrane-associated phospholipid phosphatase |  |  |  |
| SAUSA300_1311 | *murG* | UDP-N-acetylglucosamine--N-acetylmuramyl-(pentapeptide) pyrophosphoryl-undecaprenol N-acetylglucosamine transferase (EC 2.4.1.227) |  |  |  |
| SAUSA300_1312 | *-* | Acetyltransferase (EC 2.3.1.-) | 2.10 |  |  |
| SAUSA300_1313 | *ctpA* | Carboxy-terminal processing protease precursor (EC 3.4.21.102) |  |  |  |
| SAUSA300_1314 | *-* | Hypothetical cytosolic protein |  |  |  |
| SAUSA300_1315 | *crr* | PTS system, glucose-specific IIA component (EC 2.7.1.69) |  |  |  |
| SAUSA300_1316 | *msrB* | Peptide methionine sulfoxide reductase msrB (EC 1.8.4.11) |  |  |  |
| SAUSA300_1317 | *msrA* | Hypothetical protein |  |  |  |
| SAUSA300_1318 | *-* | Hypothetical protein |  |  |  |
| SAUSA300_1319 | *folA* | Dihydrofolate reductase (EC 1.5.1.3) |  |  |  |
| SAUSA300_1320 | *thyA* | Thymidylate synthase (EC 2.1.1.45) |  |  |  |
| SAUSA300_1321 | *-* | Hypothetical protein |  |  |  |
| SAUSA300_1322 | *-* | PBS lyase HEAT-like repeat |  |  |  |
| SAUSA300_1323 | *-* | PBS lyase HEAT-like repeat |  |  |  |
| SAUSA300_1324 | *-* | Hypothetical membrane spanning protein |  |  |  |
| SAUSA300_1325 | *-* | Hypothetical protein |  |  |  |
| SAUSA300_1326 | *-* | Ribonuclease HI (EC 3.1.26.4) / Cell wall enzyme EBSB |  |  |  |
| SAUSA300_1327 | *-* | Erythrocyte membrane binding protein |  |  |  |
| SAUSA300_1328 | *-* | Multidrug resistance protein B | 5.19 |  | 3.31 |
| SAUSA300_1329 | *-* | Amino acid permease | 5.86 |  | 3.00 |
| SAUSA300_1330 | *ilvA* | Threonine dehydratase (EC 4.3.1.19) | 4.17 |  | 2.57 |
| SAUSA300_1331 | *ald* | Alanine dehydrogenase (EC 1.4.1.1) | 5.62 | 2.86 |  |
| SAUSA300_1332 | *-* | 5'-3' exonuclease (EC 3.1.11.-) |  |  |  |
| SAUSA300_1333 | *-* | putative GTPases (dynamin-related) |  |  |  |
| SAUSA300_1334 | *-* | Sulfite reductase [NADPH] flavoprotein alpha-component (EC 1.8.1.2) |  |  | -2.33 |
| SAUSA300_1335 | *-* | Hypothetical protein |  |  |  |
| SAUSA300_1336 | *-* | Methyltransferase (EC 2.1.1.-) |  |  |  |
| SAUSA300_1337 | *-* | Cell division initiation protein DivIVA |  |  |  |
| SAUSA300_1338 | *-* | Hypothetical cytosolic protein |  |  |  |
| SAUSA300_1339 | *-* | Hypothetical protein |  |  |  |
| SAUSA300_1340 | *recU* | Recombination protein recU |  |  |  |
| SAUSA300_1341 | *pbp2* | Multimodular transpeptidase-transglycosylase PBP 1A |  |  |  |
| SAUSA300_1342 | *-* | Hypothetical protein |  |  |  |
| SAUSA300_1343 | *nth* | Endonuclease III (EC 4.2.99.18) |  |  |  |
| SAUSA300_1344 | *-* | DNA replication protein DnaD |  |  |  |
| SAUSA300_1345 | *asnC* | Asparaginyl-tRNA synthetase (EC 6.1.1.22) |  |  |  |
| SAUSA300_1346 | *-* | ATP-dependent helicase, DinG family |  |  |  |
| SAUSA300_1347 | *birA* | Biotin operon repressor / Biotin--[acetyl-CoA-carboxylase] synthetase (EC 6.3.4.15) |  |  |  |
| SAUSA300_1348 | *-* | tRNA nucleotidyltransferase (EC 2.7.7.25) |  |  |  |
| SAUSA300_1349 | *-* | Glycosyltransferase (EC 2.4.1.-) |  |  |  |
| SAUSA300_1350 | *-* | Pyrophosphohydrolase, MazG family |  |  |  |
| SAUSA300_1351 | *-* | Neutral zinc metallopeptidase family |  |  |  |
| SAUSA300_1352 | *-* | Hypothetical membrane spanning protein |  |  |  |
| SAUSA300_1353 | *-* | Hypothetical cytosolic protein |  |  |  |
| SAUSA300_1354 | *-* | Tetratricopeptide repeat family protein |  |  |  |
| SAUSA300_1355 | *aroA* | 3-phosphoshikimate 1-carboxyvinyltransferase (EC 2.5.1.19) |  |  |  |
| SAUSA300_1356 | *aroB* | 3-dehydroquinate synthase (EC 4.2.3.4) |  |  |  |
| SAUSA300_1357 | *aroC* | Chorismate synthase (EC 4.2.3.5) |  |  |  |
| SAUSA300_1358 | *ndk* | Nucleoside diphosphate kinase (EC 2.7.4.6) |  |  |  |
| SAUSA300_1359 | *-* | Farnesyl pyrophosphate synthetase (EC 2.5.1.1) / Geranyltranstransferase (EC 2.5.1.10) / Farnesyltransferase (EC 2.5.1.29) / Heptaprenyl |  |  |  |
| SAUSA300_1360 | *ubiE* | S-adenosylmethionine:2-demethylmenaquinone methyltransferase (EC 2.1.-.-) |  |  |  |
| SAUSA300_1361 | *-* | Heptaprenyl diphosphate synthase component I (EC 2.5.1.30) |  |  |  |
| SAUSA300_1362 | *hup* | DNA-binding protein HU |  |  |  |
| SAUSA300_1363 | *gpsA* | Glycerol-3-phosphate dehydrogenase [NAD(P)+] (EC 1.1.1.94) |  |  |  |
| SAUSA300_1364 | *engA* | GTP-binding protein |  |  |  |
| SAUSA300_1365 | *rpsA* | SSU ribosomal protein S1P |  |  |  |
| SAUSA300_1366 | *-* | Hypothetical protein |  |  |  |
| SAUSA300_1367 | *cmk* | Cytidylate kinase (EC 2.7.4.14) |  |  |  |
| SAUSA300_1368 | *ansA* | L-asparaginase (EC 3.5.1.1) |  |  |  |
| SAUSA300_1369 | *-* | Thioredoxin reductase (EC 1.8.1.9) |  |  |  |
| SAUSA300_1370 | *ebpS* | elastin binding protein |  |  |  |
| SAUSA300_1371 | *recQ* | ATP-dependent DNA helicase recQ (EC 3.6.1.-) |  |  |  |
| SAUSA300_1372 | *-* | Hypothetical protein |  |  |  |
| SAUSA300_1373 | *-* | Ferredoxin |  |  |  |
| SAUSA300_1374 | *-* | Riboflavin transporter |  |  |  |
| SAUSA300_1375 | *-* | Hypothetical protein |  |  |  |
| SAUSA300_1376 | *-* | Hypothetical cytosolic protein |  |  |  |
| SAUSA300_1377 | *-* | Hypothetical protein |  |  |  |
| SAUSA300_1378 | *-* | Hypothetical cytosolic protein |  |  |  |
| SAUSA300_1379 | *-* | Hypothetical cytosolic protein |  |  |  |
| SAUSA300_1380 | *-* | Hypothetical protein |  |  |  |
| SAUSA300_1381 | *lukF-PV* | Leukocidin F subunit | -5.11 |  | -10.70 |
| SAUSA300_1382 | *lukS-PV* | Leukocidin S subunit | -4.71 |  | -7.93 |
| SAUSA300_1383 | *-* | N-acetylmuramoyl-L-alanine amidase (EC 3.5.1.28) |  |  |  |
| SAUSA300_1384 | *-* | Holin |  |  |  |
| SAUSA300_1385 | *-* | Phage protein |  |  |  |
| SAUSA300_1386 | *-* | Phage protein |  |  |  |
| SAUSA300_1387 | *-* | Phage protein |  |  |  |
| SAUSA300_1388 | *-* | Phage protein |  |  |  |
| SAUSA300_1389 | *-* | Teichoic acid biosynthesis protein C |  |  |  |
| SAUSA300_1390 | *-* | Hypothetical protein |  |  |  |
| SAUSA300_1391 | *-* | Hypothetical protein |  |  |  |
| SAUSA300_1392 | *-* | Phage protein |  |  |  |
| SAUSA300_1393 | *-* | Phage protein |  |  |  |
| SAUSA300_1394 | *-* | Hypothetical protein |  |  |  |
| SAUSA300_1395 | *-* | Hypothetical protein |  |  |  |
| SAUSA300_1396 | *-* | Major tail protein | 2.13 |  |  |
| SAUSA300_1397 | *-* | Major tail protein |  |  |  |
| SAUSA300_1398 | *-* | Hypothetical protein |  |  |  |
| SAUSA300_1399 | *-* | Phage protein |  |  |  |
| SAUSA300_1400 | *-* | Phage protein |  |  |  |
| SAUSA300_1401 | *-* | Phage Prohead Protease |  |  |  |
| SAUSA300_1402 | *-* | ATP-dependent endopeptidase clp proteolytic subunit clpP (EC 3.4.21.92) |  |  |  |
| SAUSA300_1403 | *-* | Portal protein |  |  |  |
| SAUSA300_1404 | *-* | Terminase large subunit |  |  |  |
| SAUSA300_1405 | *-* | Terminase small subunit |  |  |  |
| SAUSA300_1406 | *-* | Phage endonuclease |  |  |  |
| SAUSA300_1407 | *-* | Transcriptional activator rinA |  |  |  |
| SAUSA300_1408 | *-* | Phage-related DNA helicase |  |  |  |
| SAUSA300_1409 | *-* | Phage-related protein |  |  |  |
| SAUSA300_1410 | *-* | Virulence-associated protein E |  |  |  |
| SAUSA300_1411 | *-* | Phage protein |  |  |  |
| SAUSA300_1412 | *-* | Transcriptional activator RinB |  |  |  |
| SAUSA300_1413 | *-* | Hypothetical protein |  |  |  |
| SAUSA300_1414 | *-* | Hypothetical protein |  |  |  |
| SAUSA300_1415 | *-* | Hypothetical protein |  |  |  |
| SAUSA300_1416 | *-* | Hypothetical protein |  |  |  |
| SAUSA300_1417 | *-* | Hypothetical cytosolic protein |  |  |  |
| SAUSA300_1418 | *-* | Phage protein |  |  |  |
| SAUSA300_1419 | *-* | Hypothetical protein |  |  |  |
| SAUSA300_1420 | *-* | Phage protein |  |  |  |
| SAUSA300_1421 | *-* | Phage protein |  |  |  |
| SAUSA300_1422 | *-* | Hypothetical cytosolic protein |  |  |  |
| SAUSA300_1423 | *polA* | PHAGE-RELATED DNA POLYMERASE (EC 2.7.7.7) |  |  |  |
| SAUSA300_1424 | *-* | Phage protein |  |  |  |
| SAUSA300_1425 | *-* | Phage protein |  |  |  |
| SAUSA300_1426 | *-* | Hypothetical protein |  |  |  |
| SAUSA300_1427 | *-* | Hypothetical cytosolic protein |  |  |  |
| SAUSA300_1428 | *-* | Hypothetical cytosolic protein |  |  |  |
| SAUSA300_1429 | *-* | Phage protein |  |  |  |
| SAUSA300_1430 | *-* | Phage protein |  |  |  |
| SAUSA300_1431 | *-* | Phage protein |  |  |  |
| SAUSA300_1432 | *-* | Hypothetical protein |  |  |  |
| SAUSA300_1433 | *-* | Transcriptional regulator, pbsX family |  |  |  |
| SAUSA300_1434 | *-* | Transcriptional regulator, MerR family |  |  |  |
| SAUSA300_1435 | *-* | Zn-dependent alcohol dehydrogenases and related dehydrogenases |  |  |  |
| SAUSA300_1436 | *-* | Hypothetical protein |  |  |  |
| SAUSA300_1437 | *-* | Hypothetical protein |  |  |  |
| SAUSA300_1438 | *-* | DNA integration/recombination/inversion protein |  |  |  |
| SAUSA300_1440 | *-* | Hypothetical cytosolic protein |  |  |  |
| SAUSA300_1441 | *srrB* | Sensor protein resE (EC 2.7.3.-) |  |  |  |
| SAUSA300_1442 | *srrA* | Transcriptional regulatory protein ResD |  |  |  |
| SAUSA300_1443 | *rluB* | Ribosomal large subunit pseudouridine synthase B (EC 4.2.1.70) |  |  |  |
| SAUSA300_1444 | *scpB* | Segregation and condensation protein ScpB |  |  |  |
| SAUSA300_1445 | *scpA* | Segregation and condensation protein ScpA |  |  |  |
| SAUSA300_1446 | *-* | Hypothetical protein |  |  |  |
| SAUSA300_1447 | *xerD* | Integrase/recombinase (XerD/RipX family) |  |  |  |
| SAUSA300_1448 | *-* | Ferric uptake regulation protein |  |  |  |
| SAUSA300_1449 | *-* | ADP-ribose pyrophosphatase (EC 3.6.1.13) |  |  |  |
| SAUSA300_1450 | *-* | Oxidoreductase (EC 1.1.1.-) |  |  |  |
| SAUSA300_1451 | *-* | Hypothetical protein |  |  |  |
| SAUSA300_1452 | *proC* | Pyrroline-5-carboxylate reductase (EC 1.5.1.2) |  |  |  |
| SAUSA300_1453 | *-* | Ribonuclease Z (EC 3.1.26.11) |  |  |  |
| SAUSA300_1454 | *zwf* | Glucose-6-phosphate 1-dehydrogenase (EC 1.1.1.49) |  |  |  |
| SAUSA300_1455 | *-* | Transcriptional activator aarP |  |  |  |
| SAUSA300_1456 | *-* | Exo-alpha-1,4-glucosidase (EC 3.2.1.20) |  |  |  |
| SAUSA300_1457 | *malR* | Maltose operon transcriptional repressor | -2.28 |  |  |
| SAUSA300_1458 | *-* | Lactoylglutathione lyase (EC 4.4.1.5) |  |  |  |
| SAUSA300_1459 | *gnd* | 6-phosphogluconate dehydrogenase (EC 1.1.1.44) |  |  |  |
| SAUSA300_1460 | *-* | Tripeptidase T (EC 3.4.11.4) |  |  |  |
| SAUSA300_1461 | *-* | Hypothetical protein |  |  |  |
| SAUSA300_1462 | *-* | Integral membrane protein |  |  |  |
| SAUSA300_1463 | *-* | Hypothetical protein |  |  |  |
| SAUSA300_1464 | *-* | Lipoamide acyltransferase component of branched-chain alpha-keto acid dehydrogenase complex (EC 2.3.1.-) |  |  |  |
| SAUSA300_1465 | *-* | 2-oxoisovalerate dehydrogenase beta subunit (EC 1.2.4.4) |  |  |  |
| SAUSA300_1466 | *-* | 2-oxoisovalerate dehydrogenase alpha subunit (EC 1.2.4.4) |  |  |  |
| SAUSA300_1467 | *lpdA* | Dihydrolipoamide dehydrogenase (EC 1.8.1.4) |  |  |  |
| SAUSA300_1468 | *recN* | DNA repair protein recN |  |  |  |
| SAUSA300_1469 | *argR* | Arginine repressor, argR |  |  |  |
| SAUSA300_1470 | *-* | Dimethylallyltransferase (EC 2.5.1.1) / Geranyltranstransferase (EC 2.5.1.10) |  |  |  |
| SAUSA300_1471 | *xseB* | Exodeoxyribonuclease VII small subunit (EC 3.1.11.6) |  |  |  |
| SAUSA300_1472 | *xseA* | Exodeoxyribonuclease VII large subunit (EC 3.1.11.6) |  |  |  |
| SAUSA300_1473 | *nusB* | N utilization substance protein B |  |  |  |
| SAUSA300_1474 | *-* | General stress protein, Gls24 family |  |  |  |
| SAUSA300_1475 | *accC* | Biotin carboxylase (EC 6.3.4.14) |  |  |  |
| SAUSA300_1476 | *accB* | Biotin carboxyl carrier protein of acetyl-CoA carboxylase (EC 6.4.1.2) |  |  |  |
| SAUSA300_1478 | *-* | Hypothetical protein |  |  |  |
| SAUSA300_1479 | *-* | Hypothetical protein |  |  |  |
| SAUSA300_1480 | *-* | Secretory antigen precursor SsaA |  |  |  |
| SAUSA300_1481 | *-* | Hypothetical protein |  |  |  |
| SAUSA300_1482 | *-* | FtsK/SpoIIIE family |  |  |  |
| SAUSA300_1483 | *-* | DNA segregation ATPase and related proteins |  |  |  |
| SAUSA300_1484 | *-* | Hypothetical protein |  |  |  |
| SAUSA300_1485 | *-* | Hypothetical protein |  |  |  |
| SAUSA300_1486 | *-* | Hypothetical protein |  |  |  |
| SAUSA300_1487 | *-* | Transcriptional regulator, Cro/CI family |  |  |  |
| SAUSA300_1488 | *-* | Hypothetical protein |  |  |  |
| SAUSA300_1489 | *-* | Hypothetical protein |  |  |  |
| SAUSA300_1490 | *efp* | Protein Translation Elongation Factor P (EF-P) |  |  |  |
| SAUSA300_1491 | *-* | Xaa-Pro dipeptidase (EC 3.4.13.9) |  |  |  |
| SAUSA300_1492 | *-* | Hypothetical protein |  |  |  |
| SAUSA300_1493 | *-* | Hypothetical protein |  |  |  |
| SAUSA300_1494 | *-* | Lipoate-protein ligase A (EC 6.3.2.-) |  |  |  |
| SAUSA300_1495 | *-* | Rhodanese-related sulfurtransferases |  |  |  |
| SAUSA300_1496 | *-* | Glycine dehydrogenase [decarboxylating] (EC 1.4.4.2) |  |  |  |
| SAUSA300_1497 | *-* | Glycine dehydrogenase [decarboxylating] (EC 1.4.4.2) |  |  |  |
| SAUSA300_1498 | *gcvT* | Aminomethyltransferase (EC 2.1.2.10) |  |  |  |
| SAUSA300_1499 | *aroK* | Shikimate kinase (EC 2.7.1.71) |  |  |  |
| SAUSA300_1500 | *-* | Hypothetical membrane associated protein |  |  |  |
| SAUSA300_1501 | *-* | ComG operon protein 4 |  |  |  |
| SAUSA300_1502 | *-* | ComG operon protein 3 |  |  |  |
| SAUSA300_1503 | *-* | ComG operon protein 2 |  |  |  |
| SAUSA300_1504 | *-* | ComG operon protein 1 |  |  |  |
| SAUSA300_1505 | *-* | Hydroxyacylglutathione hydrolase (EC 3.1.2.6) |  |  |  |
| SAUSA300_1506 | *-* | Hypothetical cytosolic protein |  |  |  |
| SAUSA300_1507 | *glk* | Glucokinase (EC 2.7.1.2) |  |  |  |
| SAUSA300_1508 | *-* | Hypothetical cytosolic protein |  |  |  |
| SAUSA300_1509 | *-* | Integral membrane protein (Rhomboid family) |  |  |  |
| SAUSA300_1510 | *-* | 5-formyltetrahydrofolate cyclo-ligase (EC 6.3.3.2) |  |  |  |
| SAUSA300_1511 | *rpmG* | LSU ribosomal protein L33P |  |  |  |
| SAUSA300_1512 | *pbp3* | Penicillin binding protein transpeptidase |  |  |  |
| SAUSA300_1513 | *-* | Superoxide dismutase (EC 1.15.1.1) |  |  |  |
| SAUSA300_1514 | *zur* | Zinc-specific metalloregulatory protein |  |  |  |
| SAUSA300_1515 | *-* | High-affinity zinc uptake system membrane protein ZnuB |  |  |  |
| SAUSA300_1516 | *-* | High-affinity zinc uptake system ATP-binding protein ZnuC |  |  |  |
| SAUSA300_1517 | *-* | Endonuclease IV (EC 3.1.21.2) |  |  |  |
| SAUSA300_1518 | *-* | ATP-dependent RNA helicase |  |  |  |
| SAUSA300_1519 | *-* | NIF3-related protein |  |  |  |
| SAUSA300_1520 | *-* | Hypothetical cytosolic protein |  |  |  |
| SAUSA300_1521 | *rpoD* | RNA polymerase sigma factor RpoD |  |  |  |
| SAUSA300_1522 | *dnaG* | DNA primase (EC 2.7.7.-) |  |  |  |
| SAUSA300_1523 | *-* | ATP/GTP-binding protein |  |  |  |
| SAUSA300_1524 | *-* | CBS domain containing protein |  |  |  |
| SAUSA300_1525 | *glyS* | Glycyl-tRNA synthetase (EC 6.1.1.14) |  |  |  |
| SAUSA300_1526 | *recO* | DNA repair protein recO |  |  |  |
| SAUSA300_1527 | *era* | GTP-binding protein era |  |  |  |
| SAUSA300_1528 | *cdd* | Cytidine deaminase (EC 3.5.4.5) |  |  |  |
| SAUSA300_1529 | *dgkA* | Diacylglycerol kinase (EC 2.7.1.107) |  |  |  |
| SAUSA300_1530 | *-* | Hypothetical metal-binding protein |  |  |  |
| SAUSA300_1531 | *phoH* | PhoH protein |  |  |  |
| SAUSA300_1532 | *-* | Siderophore-mediated iron transport protein |  |  |  |
| SAUSA300_1533 | *-* | Hypothetical protein |  |  |  |
| SAUSA300_1534 | *-* | Transporter |  |  |  |
| SAUSA300_1535 | *rpsU* | SSU ribosomal protein S21P |  |  |  |
| SAUSA300_1536 | *-* | tRNA 2-methylthioadenosine synthase homolog |  |  |  |
| SAUSA300_1537 | *-* | Hypothetical cytosolic protein |  |  |  |
| SAUSA300_1538 | *prmA* | Ribosomal protein L11 methyltransferase (EC 2.1.1.-) |  |  |  |
| SAUSA300_1539 | *dnaJ* | Chaperone protein DnaJ |  |  |  |
| SAUSA300_1540 | *dnaK* | Chaperone protein DnaK |  |  |  |
| SAUSA300_1541 | *grpE* | GrpE protein |  |  |  |
| SAUSA300_1542 | *hrcA* | Heat-inducible transcription repressor HrcA |  |  |  |
| SAUSA300_1543 | *-* | Oxygen-independent coproporphyrinogen-III oxidase (EC 1.3.99.22) |  |  |  |
| SAUSA300_1544 | *lepA* | GTP-binding protein lepA |  |  |  |
| SAUSA300_1545 | *rpsT* | SSU ribosomal protein S20P |  |  |  |
| SAUSA300_1546 | *holA* | DNA polymerase III, delta subunit (EC 2.7.7.7) |  |  |  |
| SAUSA300_1547 | *-* | COME operon protein 3 |  |  |  |
| SAUSA300_1548 | *-* | COME operon protein 2 |  |  |  |
| SAUSA300_1549 | *-* | COME operon protein 1 |  |  |  |
| SAUSA300_1550 | *-* | Methyltransferase (EC 2.1.1.-) |  |  |  |
| SAUSA300_1551 | *-* | iojap protein family |  |  |  |
| SAUSA300_1552 | *-* | Hydrolase (HAD superfamily) |  |  |  |
| SAUSA300_1553 | *nadD* | Nicotinate-nucleotide adenylyltransferase (EC 2.7.7.18) |  |  |  |
| SAUSA300_1554 | *-* | Hypothetical RNA binding protein |  |  |  |
| SAUSA300_1555 | *aroE* | Shikimate 5-dehydrogenase (EC 1.1.1.25) |  |  |  |
| SAUSA300_1556 | *-* | GTP-binding protein |  |  |  |
| SAUSA300_1557 | *-* | Putative lipase |  |  |  |
| SAUSA300_1558 | *mtnN* | 5'-methylthioadenosine nucleosidase (EC 3.2.2.16) / S-adenosylhomocysteine nucleosidase (EC 3.2.2.9) |  |  |  |
| SAUSA300_1559 | *-* | Enterotoxin |  |  |  |
| SAUSA300_1560 | *-* | Fic family protein |  |  |  |
| SAUSA300_1561 | *-* | Hypothetical membrane spanning protein |  |  |  |
| SAUSA300_1562 | *-* | Lactam utilization protein LamB |  |  |  |
| SAUSA300_1563 | *accC* | Biotin carboxylase (EC 6.3.4.14) |  |  |  |
| SAUSA300_1564 | *accB* | Biotin carboxyl carrier protein of acetyl-CoA carboxylase |  |  |  |
| SAUSA300_1565 | *-* | Regulator of kinase autophosphorylation inhibitor |  |  |  |
| SAUSA300_1566 | *-* | Kinase autophosphorylation inhibitor KipI |  |  |  |
| SAUSA300_1567 | *greA* | Transcription elongation factor greA |  |  |  |
| SAUSA300_1568 | *udk* | Uridine kinase (EC 2.7.1.48) |  |  |  |
| SAUSA300_1569 | *-* | Peptidase family U32 |  |  |  |
| SAUSA300_1570 | *-* | Peptidase family U32 |  |  |  |
| SAUSA300_1571 | *-* | O-methyltransferase (EC 2.1.1.-) |  |  |  |
| SAUSA300_1572 | *-* | Hypothetical cytosolic protein |  |  |  |
| SAUSA300_1573 | *-* | Endonuclease involved in recombination |  |  |  |
| SAUSA300_1574 | *-* | Hypothetical cytosolic protein |  |  |  |
| SAUSA300_1575 | *alaS* | Alanyl-tRNA synthetase (EC 6.1.1.7) |  |  |  |
| SAUSA300_1576 | *-* | Exodeoxyribonuclease V alpha chain (EC 3.1.11.5) |  |  |  |
| SAUSA300_1577 | *-* | O-LINKED GLCNAC TRANSFERASE |  |  |  |
| SAUSA300_1578 | *mnmA* | tRNA (5-methylaminomethyl-2-thiouridylate)-methyltransferase (EC 2.1.1.61) |  |  |  |
| SAUSA300_1579 | *-* | Cysteine desulfurase (EC 2.8.1.7) / Selenocysteine lyase (EC 4.4.1.16) |  |  |  |
| SAUSA300_1580 | *-* | Luciferase-like monooxygenase (EC 1.14.-.-) |  |  |  |
| SAUSA300_1581 | *-* | Hypothetical protein |  |  |  |
| SAUSA300_1582 | *-* | Hypothetical protein |  |  |  |
| SAUSA300_1583 | *-* | Rrf2 family protein |  |  |  |
| SAUSA300_1584 | *-* | ATPase, AAA family |  |  |  |
| SAUSA300_1585 | *-* | ThiF/MoeB family protein |  |  |  |
| SAUSA300_1586 | *aspS* | Aspartyl-tRNA synthetase (EC 6.1.1.12) |  |  |  |
| SAUSA300_1587 | *hisS* | Histidyl-tRNA synthetase (EC 6.1.1.21) |  |  |  |
| SAUSA300_1588 | *lytH* | N-acetylmuramoyl-L-alanine amidase (EC 3.5.1.28) |  |  |  |
| SAUSA300_1589 | *dtd* | D-tyrosyl-tRNA(Tyr) deacylase (EC 3.1.-.-) |  |  |  |
| SAUSA300_1590 | *-* | GTP pyrophosphokinase (EC 2.7.6.5) / Guanosine-3',5'-bis(Diphosphate) 3'-pyrophosphohydrolase (EC 3.1.7.2) |  |  |  |
| SAUSA300_1591 | *apt* | Adenine phosphoribosyltransferase (EC 2.4.2.7) |  |  |  |
| SAUSA300_1592 | *recJ* | Single-stranded-DNA-specific exonuclease recJ (EC 3.1.-.-) |  |  | 2.20 |
| SAUSA300_1593 | *secF* | Protein translocase subunit secD / Protein translocase subunit secF |  |  |  |
| SAUSA300_1594 | *yajC* | Protein translocase subunit YajC |  |  |  |
| SAUSA300_1595 | *tgt* | Queuine tRNA-ribosyltransferase (EC 2.4.2.29) |  |  |  |
| SAUSA300_1596 | *queA* | S-adenosylmethionine:tRNA ribosyltransferase-isomerase (EC 5.-.-.-) |  |  |  |
| SAUSA300_1597 | *ruvB* | Holliday junction DNA helicase ruvB |  |  |  |
| SAUSA300_1598 | *ruvA* | Holliday junction DNA helicase ruvA |  |  |  |
| SAUSA300_1599 | *-* | Chorismate mutase (EC 5.4.99.5) |  |  |  |
| SAUSA300_1600 | *obgE* | GTP-binding protein CgtA (probably involved in DNA repair) |  |  |  |
| SAUSA300_1601 | *rpmA* | LSU ribosomal protein L27P |  |  |  |
| SAUSA300_1602 | *-* | hypothetical ribosome-associated protein |  |  |  |
| SAUSA300_1603 | *rplU* | LSU ribosomal protein L21P |  |  |  |
| SAUSA300_1604 | *mreD* | Rod shape-determining protein mreD |  |  |  |
| SAUSA300_1605 | *mreC* | Rod shape-determining protein mreC |  |  |  |
| SAUSA300_1606 | *-* | Hypothetical protein |  |  | -3.97 |
| SAUSA300_1607 | *-* | Hypothetical protein |  |  |  |
| SAUSA300_1608 | *radC* | DNA repair protein RadC | -2.53 |  |  |
| SAUSA300_1609 | *-* | Type 4 prepilin-like proteins leader peptide processing enzyme |  |  |  |
| SAUSA300_1610 | *folC* | Folylpolyglutamate synthase (EC 6.3.2.17) / Dihydrofolate synthase (EC 6.3.2.12) |  |  |  |
| SAUSA300_1611 | *valS* | Valyl-tRNA synthetase (EC 6.1.1.9) |  |  |  |
| SAUSA300_1612 | *tag* | DNA-3-methyladenine glycosylase (EC 3.2.2.20) |  |  |  |
| SAUSA300_1613 | *-* | AbrB protein |  |  |  |
| SAUSA300_1614 | *hemL* | Glutamate-1-semialdehyde 2,1-aminomutase (EC 5.4.3.8) |  |  |  |
| SAUSA300_1615 | *hemB* | Delta-aminolevulinic acid dehydratase (EC 4.2.1.24) |  |  |  |
| SAUSA300_1616 | *hemD* | Uroporphyrinogen-III synthase (EC 4.2.1.75) |  |  |  |
| SAUSA300_1617 | *hemC* | Porphobilinogen deaminase (EC 2.5.1.61) |  |  |  |
| SAUSA300_1618 | *hemX* | Putative heme export permease |  |  |  |
| SAUSA300_1619 | *hemA* | Glutamyl-tRNA reductase (EC 1.2.1.-) |  |  |  |
| SAUSA300_1620 | *engB* | GTP-binding protein YihA |  |  |  |
| SAUSA300_1621 | *clpX* | ATP-dependent endopeptidase clp ATP-binding subunit ClpX |  |  |  |
| SAUSA300_1622 | *tig* | Trigger factor, ppiase (EC 5.2.1.8) |  |  |  |
| SAUSA300_1623 | *-* | Hypothetical protein |  |  |  |
| SAUSA300_1624 | *-* | Hypothetical cytosolic protein |  |  |  |
| SAUSA300_1625 | *rplT* | LSU ribosomal protein L20P |  |  |  |
| SAUSA300_1626 | *rpmI* | LSU ribosomal protein L35P |  |  |  |
| SAUSA300_1627 | *infC* | Bacterial Protein Translation Initiation Factor 3 (IF-3) |  |  |  |
| SAUSA300_1628 | *lysP* | Lysine-specific permease |  |  |  |
| SAUSA300_1629 | *thrS* | Threonyl-tRNA synthetase (EC 6.1.1.3) |  |  |  |
| SAUSA300_1630 | *dnaI* | Primosomal protein dnaI |  |  |  |
| SAUSA300_1631 | *-* | Replication initiation and membrane attachment protein |  |  |  |
| SAUSA300_1632 | *nrdR* | Putative regulatory protein |  |  |  |
| SAUSA300_1633 | *gap* | Glyceraldehyde 3-phosphate dehydrogenase (EC 1.2.1.12) | -2.65 |  |  |
| SAUSA300_1634 | *coaE* | Dephospho-CoA kinase (EC 2.7.1.24) |  |  |  |
| SAUSA300_1635 | *mutM* | Formamidopyrimidine-DNA glycosylase (EC 3.2.2.23) |  |  |  |
| SAUSA300_1636 | *polA* | DNA polymerase I (EC 2.7.7.7) |  |  |  |
| SAUSA300_1637 | *-* | Glucosyltransferase (side chain biosynthesis) (EC 2.4.1.-) |  |  |  |
| SAUSA300_1638 | *phoR* | Phosphate regulon sensor protein phoR (EC 2.7.3.-) |  |  |  |
| SAUSA300_1639 | *phoP* | Alkaline phosphatase synthesis two-component response regulator phoP |  |  |  |
| SAUSA300_1640 | *icd* | Isocitrate dehydrogenase [NADP] (EC 1.1.1.42) |  |  |  |
| SAUSA300_1641 | *gltA* | Citrate synthase (EC 2.3.3.1) | -2.01 |  | -2.18 |
| SAUSA300_1642 | *-* | D-serine/D-alanine/glycine transporter |  |  |  |
| SAUSA300_1644 | *pyk* | Pyruvate kinase (EC 2.7.1.40) |  |  |  |
| SAUSA300_1645 | *pfkA* | 6-phosphofructokinase (EC 2.7.1.11) |  |  |  |
| SAUSA300_1646 | *accA* | Acetyl-coenzyme A carboxylase carboxyl transferase subunit alpha (EC 6.4.1.2) |  |  |  |
| SAUSA300_1647 | *accD* | Acetyl-coenzyme A carboxylase carboxyl transferase subunit beta (EC 6.4.1.2) |  |  |  |
| SAUSA300_1648 | *-* | NAD-dependent malic enzyme (EC 1.1.1.38) |  |  |  |
| SAUSA300_1649 | *dnaE* | DNA polymerase III alpha subunit (EC 2.7.7.7) |  |  |  |
| SAUSA300_1650 | *-* | DNA polymerase III alpha subunit (EC 2.7.7.7) |  |  |  |
| SAUSA300_1651 | *-* | Cytosolic protein containing multiple CBS domains |  |  |  |
| SAUSA300_1652 | *-* | Putative transcription factors |  |  |  |
| SAUSA300_1653 | *-* | Metal-dependent hydrolase (EC 3.-.-.-) |  |  |  |
| SAUSA300_1654 | *-* | Xaa-Pro aminopeptidase (EC 3.4.11.9) |  |  |  |
| SAUSA300_1655 | *ald* | Alanine dehydrogenase (EC 1.4.1.1) | -3.84 |  | -5.24 |
| SAUSA300_1656 | *-* | Universal stress protein family |  |  |  |
| SAUSA300_1657 | *ackA* | Acetate kinase (EC 2.7.2.1) |  |  |  |
| SAUSA300_1658 | *-* | Adenine-specific methyltransferase (EC 2.1.1.72) |  |  |  |
| SAUSA300_1659 | *tpx* | Thioredoxin peroxidase (EC 1.11.1.15) |  |  |  |
| SAUSA300_1660 | *-* | Hypothetical membrane spanning protein |  |  |  |
| SAUSA300_1661 | *thiI* | Thiamine biosynthesis protein thiI | 2.07 |  |  |
| SAUSA300_1662 | *-* | Cysteine desulfurase (EC 2.8.1.7) / Selenocysteine lyase (EC 4.4.1.16) | 2.35 |  |  |
| SAUSA300_1663 | *-* | Hypothetical protein |  |  |  |
| SAUSA300_1664 | *ezrA* | Septation ring formation regulator |  |  |  |
| SAUSA300_1665 | *-* | GAF domain-containing proteins |  |  |  |
| SAUSA300_1666 | *rpsD* | SSU ribosomal protein S4P |  |  |  |
| SAUSA300_1667 |  |  |  |  |  |
| SAUSA300_1668 | *-* | Osmotically inducible protein C |  |  |  |
| SAUSA300_1669 | *-* | Serine--pyruvate aminotransferase (EC 2.6.1.51) |  |  |  |
| SAUSA300_1670 | *serA* | D-3-phosphoglycerate dehydrogenase (EC 1.1.1.95) |  |  |  |
| SAUSA300_1671 | *-* | Hpr(Ser) kinase (EC 2.7.1.-) / phosphatase (EC 3.1.3.-) |  |  |  |
| SAUSA300_1672 | *nagE* | PTS system, N-acetylglucosamine-specific IIBC component (EC 2.7.1.69) |  |  |  |
| SAUSA300_1673 | *-* | 1-acyl-sn-glycerol-3-phosphate acyltransferase (EC 2.3.1.51) |  |  |  |
| SAUSA300_1674 | *-* | Endopeptidase DegP (EC 3.4.21.-) |  |  |  |
| SAUSA300_1675 | *tyrS* | Tyrosyl-tRNA synthetase (EC 6.1.1.1) | -2.24 |  |  |
| SAUSA300_1676 | *sgtA* | Penicillin-binding protein |  |  |  |
| SAUSA300_1677 | *-* | Fibronectin-binding protein |  |  |  |
| SAUSA300_1678 | *fhs* | Formate--tetrahydrofolate ligase (EC 6.3.4.3) |  |  | -2.18 |
| SAUSA300_1679 | *acsA* | Acetyl-coenzyme A synthetase (EC 6.2.1.1) | -3.60 |  | -3.02 |
| SAUSA300_1680 | *acuA* | Acetoin utilization protein acuA (EC 2.3.1.-) | -2.40 |  |  |
| SAUSA300_1681 | *acuC* | Acetoin utilization acuC protein | -2.42 |  |  |
| SAUSA300_1682 | *ccpA* | Catabolite control protein A |  |  |  |
| SAUSA300_1683 | *-* | 3-deoxy-7-phosphoheptulonate synthase (EC 2.5.1.54) / Chorismate mutase (EC 5.4.99.5) |  |  |  |
| SAUSA300_1684 | *-* | Hypothetical exported protein |  |  |  |
| SAUSA300_1685 | *-* | General stress protein |  |  |  |
| SAUSA300_1686 | *murC* | UDP-N-acetylmuramate--alanine ligase (EC 6.3.2.8) |  |  |  |
| SAUSA300_1687 | *-* | Cell division protein FtsK |  |  |  |
| SAUSA300_1688 | *-* | tRNA binding domain protein |  |  |  |
| SAUSA300_1689 | *-* | Hypothetical cytosolic protein |  |  |  |
| SAUSA300_1690 | *-* | Thioredoxin |  |  |  |
| SAUSA300_1691 | *-* | Hypothetical protein |  |  |  |
| SAUSA300_1692 | *-* | Hypothetical protein |  |  |  |
| SAUSA300_1693 | *-* | Metal-dependent hydrolase (EC 3.-.-.-) |  |  |  |
| SAUSA300_1694 | *trmB* | tRNA (m(7)G46) methyltransferase (EC 2.1.1.33) |  |  |  |
| SAUSA300_1695 | *-* | Phosphotransferase enzyme family |  |  |  |
| SAUSA300_1696 | *dat* | D-alanine aminotransferase (EC 2.6.1.21) |  |  |  |
| SAUSA300_1697 | *-* | Xaa-His dipeptidase (EC 3.4.13.3) |  |  |  |
| SAUSA300_1698 | *-* | Hypothetical protein |  |  |  |
| SAUSA300_1699 | *-* | Ribosomal small subunit pseudouridine synthase A (EC 4.2.1.70) |  |  |  |
| SAUSA300_1700 | *-* | Export protein for polysaccharides and teichoic acids |  |  |  |
| SAUSA300_1701 | *-* | NAD(FAD)-utilizing dehydrogenases |  |  |  |
| SAUSA300_1702 | *-* | Extracellular matrix binding protein |  |  |  |
| SAUSA300_1703 | *-* | Rhodanese-related sulfurtransferases |  |  |  |
| SAUSA300_1704 | *leuS* | Leucyl-tRNA synthetase (EC 6.1.1.4) |  |  |  |
| SAUSA300_1705 | *-* | Multidrug resistance protein |  |  |  |
| SAUSA300_1706 | *-* | Radical SAM superfamily protein |  |  |  |
| SAUSA300_1707 | *-* | SAM-dependent methyltransferase (EC 2.1.-.-) | 2.20 |  |  |
| SAUSA300_1708 | *rot* | Staphylococcal accessory regulator |  |  |  |
| SAUSA300_1710 | *-* | Lysophospholipase L2 (EC 3.1.1.5) |  |  |  |
| SAUSA300_1711 | *putA* | Proline dehydrogenase (EC 1.5.99.8) | -2.37 |  |  |
| SAUSA300_1712 | *ribH* | 6,7-dimethyl-8-ribityllumazine synthase (EC 2.5.1.9) | -3.62 | -2.27 |  |
| SAUSA300_1713 | *ribBA* | GTP cyclohydrolase II (EC 3.5.4.25) / 3,4-dihydroxy-2-butanone-4-phosphate synthase (EC 4.1.2.-) | -3.53 |  |  |
| SAUSA300_1714 | *ribE* | Riboflavin synthase alpha chain (EC 2.5.1.9) | -3.49 | -2.27 |  |
| SAUSA300_1715 | *ribD* | Diaminohydroxyphosphoribosylaminopyrimidine deaminase (EC 3.5.4.26) / 5-amino-6-(5-phosphoribosylamino)uracil reductase (EC 1.1.1.193) | -3.11 |  | -2.37 |
| SAUSA300_1716 | *-* | Hypothetical exported protein |  |  |  |
| SAUSA300_1717 | *arsR* | Arsenical resistance operon repressor |  |  |  |
| SAUSA300_1718 | *arsB* | Arsenical pump membrane protein |  |  |  |
| SAUSA300_1719 | *arsC* | Arsenate reductase (EC 1.20.4.1) |  |  |  |
| SAUSA300_1720 | *-* | Peptidoglycan endo-beta-N-acetylglucosaminidase (EC 3.2.1.-) |  |  |  |
| SAUSA300_1721 | *-* | Hypothetical protein |  |  |  |
| SAUSA300_1722 | *-* | Hypothetical protein |  |  |  |
| SAUSA300_1723 | *-* | Hypothetical protein |  |  |  |
| SAUSA300_1724 | *-* | Hypothetical membrane spanning protein |  |  |  |
| SAUSA300_1725 | *-* | Transaldolase (EC 2.2.1.2) |  |  |  |
| SAUSA300_1726 | *-* | CrcB family protein |  |  |  |
| SAUSA300_1728 | *-* | 2,5-diketo-D-gluconic acid reductase (EC 1.1.1.274) |  |  |  |
| SAUSA300_1729 | *-* | Hypothetical protein |  |  |  |
| SAUSA300_1730 | *metK* | S-adenosylmethionine synthetase (EC 2.5.1.6) |  |  |  |
| SAUSA300_1731 | *pckA* | Phosphoenolpyruvate carboxykinase [ATP] (EC 4.1.1.49) | -6.03 |  | -3.83 |
| SAUSA300_1733 | *-* | Acylamino-acid-releasing enzyme (EC 3.4.19.1) |  |  |  |
| SAUSA300_1734 | *-* | Phosphohydrolase (MutT/nudix family protein) |  |  |  |
| SAUSA300_1735 | *menC* | O-succinylbenzoate synthase (EC 4.2.1.-) |  |  |  |
| SAUSA300_1736 | *-* | Hypothetical cytosolic protein |  |  |  |
| SAUSA300_1737 | *menE* | O-succinylbenzoic acid--CoA ligase (EC 6.2.1.26) |  |  |  |
| SAUSA300_1738 | *-* | Hypothetical protein | 2.38 |  |  |
| SAUSA300_1739 | *-* | Endonuclease (EC 3.1.-.-) |  |  |  |
| SAUSA300_1740 | *-* | Hypothetical protein |  |  |  |
| SAUSA300_1741 | *-* | Hypothetical protein |  |  |  |
| SAUSA300_1742 | *-* | Hypothetical protein |  |  |  |
| SAUSA300_1743 | *-* | Hypothetical protein |  |  |  |
| SAUSA300_1744 | *-* | Hypothetical protein |  |  |  |
| SAUSA300_1745 | *-* | Hypothetical protein |  |  |  |
| SAUSA300_1746 | *-* | Hypothetical protein |  |  |  |
| SAUSA300_1747 | *-* | Hypothetical protein |  |  |  |
| SAUSA300_1749 | *-* | Hypothetical protein |  |  |  |
| SAUSA300_1750 | *-* | Hypothetical protein |  |  |  |
| SAUSA300_1751 | *hsdS* | Type I restriction-modification system specificity subunit |  |  |  |
| SAUSA300_1752 | *hsdM* | Type I restriction-modification system methylation subunit |  |  |  |
| SAUSA300_1753 | *splF* | Serine protease (EC 3.4.21.-) | -11.95 |  | -15.08 |
| SAUSA300_1754 | *splE* | Serine protease (EC 3.4.21.-) | -7.58 |  | -8.96 |
| SAUSA300_1755 | *splD* | Serine protease (EC 3.4.21.-) | -8.11 |  | -12.11 |
| SAUSA300_1756 | *splC* | Serine protease (EC 3.4.21.-) | -7.33 |  | -7.48 |
| SAUSA300_1757 | *splB* | Serine protease (EC 3.4.21.-) | -14.99 |  | -15.40 |
| SAUSA300_1758 | *splA* | Serine protease (EC 3.4.21.-) | -15.31 |  | -15.17 |
| SAUSA300_1759 | *-* | Hypothetical protein | -4.90 |  | -3.93 |
| SAUSA300_1760 | *epiG* | Epidermin resistance transmembrane protein |  |  |  |
| SAUSA300_1761 | *epiE* | Lantibiotic transport permease protein |  |  |  |
| SAUSA300_1762 | *epiF* | Lantibiotic transport ATP-binding protein |  |  |  |
| SAUSA300_1763 | *epiP* | Epidermin leader peptide processing serine protease EPIP precursor (EC 3.4.21.-) |  |  |  |
| SAUSA300_1764 | *epiD* | Epidermin modifying enzyme epiD |  |  |  |
| SAUSA300_1765 | *epiC* | Lanthionine synthetase (lantibiotic biosynthesis) |  |  |  |
| SAUSA300_1766 | *epiB* | Serine (threonine) dehydratase (lantibiotic biosynthesis) |  |  |  |
| SAUSA300_1767 | *epiA* | Lantibiotic gallidermin |  |  |  |
| SAUSA300_1768 | *lukD* | Hypothetical protein |  |  |  |
| SAUSA300_1769 | *lukE* | Leukocidin S subunit |  |  |  |
| SAUSA300_1770 | *-* | Hypothetical protein |  |  |  |
| SAUSA300_1771 | *-* | Hypothetical protein |  |  |  |
| SAUSA300_1780 | *-* | Hypothetical protein |  |  |  |
| SAUSA300_1781 | *hemG* | Protoporphyrinogen oxidase (EC 1.3.3.4) |  |  |  |
| SAUSA300_1782 | *hemH* | Ferrochelatase (EC 4.99.1.1) |  |  |  |
| SAUSA300_1783 | *hemE* | Uroporphyrinogen decarboxylase (EC 4.1.1.37) |  |  |  |
| SAUSA300_1784 | *-* | Signal transduction protein TRAP |  |  |  |
| SAUSA300_1785 | *-* | Protein ecsB |  |  |  |
| SAUSA300_1786 | *-* | ABC-type transporter ATP-binding protein ecsA |  |  |  |
| SAUSA300_1787 | *-* | Adenosine 5'-monophosphoramidase |  |  |  |
| SAUSA300_1788 | *-* | Hypothetical protein |  |  |  |
| SAUSA300_1789 | *-* | Hypothetical protein |  |  |  |
| SAUSA300_1790 | *prsA* | Peptidyl-prolyl cis-trans isomerase (EC 5.2.1.8) | -2.05 |  | -2.95 |
| SAUSA300_1791 | *cbf1* | CMP-binding factor |  |  |  |
| SAUSA300_1792 | *-* | Hypothetical membrane associated protein |  |  |  |
| SAUSA300_1793 | *-* | Phosphoesterase |  |  |  |
| SAUSA300_1794 | *-* | Hypothetical protein |  |  |  |
| SAUSA300_1795 | *-* | Hypothetical cytosolic protein |  |  |  |
| SAUSA300_1796 | *-* | Hypothetical membrane associated protein |  |  |  |
| SAUSA300_1797 | *-* | Transcriptional regulator, PbsX family |  |  |  |
| SAUSA300_1798 | *-* | Hypothetical protein |  |  |  |
| SAUSA300_1799 | *-* | Two-component sensor protein YhcY (EC 2.7.3.-) |  |  |  |
| SAUSA300_1800 | *-* | Ribosomal large subunit pseudouridine synthase D (EC 4.2.1.70) |  |  |  |
| SAUSA300_1801 | *fumC* | Fumarate hydratase (EC 4.2.1.2) |  |  |  |
| SAUSA300_1802 | *-* | Hypothetical protein |  |  |  |
| SAUSA300_1803 | *-* | Hypothetical protein |  |  |  |
| SAUSA300_1804 | *-* | Hypothetical protein |  |  |  |
| SAUSA300_1805 | *-* | 23S rRNA methyltransferase (EC 2.1.1.-) |  |  |  |
| SAUSA300_1806 | *-* | iron-sulfur cluster-binding protein |  |  |  |
| SAUSA300_1807 | *-* | Arginine transport ATP-binding protein ArtP |  |  |  |
| SAUSA300_1808 | *-* | Arginine-binding protein / Arginine transport system permease protein ArtQ |  |  |  |
| SAUSA300_1809 | *-* | Hypothetical membrane spanning protein |  |  |  |
| SAUSA300_1810 | *-* | Transposase |  |  |  |
| SAUSA300_1842 | *-* | Peroxide operon regulator |  |  |  |
| SAUSA300_1843 | *-* | D-3-phosphoglycerate dehydrogenase (EC 1.1.1.95) |  |  |  |
| SAUSA300_1844 | *-* | Thioredoxin peroxidase (EC 1.11.1.15) |  |  |  |
| SAUSA300_1845 | *hemL* | Glutamate-1-semialdehyde 2,1-aminomutase (EC 5.4.3.8) |  |  |  |
| SAUSA300_1846 | *-* | Integral membrane protein |  |  |  |
| SAUSA300_1847 | *-* | Multidrug/protein/lipid ABC transporter family, ATP-binding and permease protein |  |  |  |
| SAUSA300_1848 | *-* | Hypothetical cytosolic protein |  |  |  |
| SAUSA300_1849 | *mutY* | A/G-specific adenine DNA glycosylase (EC 3.2.2.-) |  |  |  |
| SAUSA300_1850 | *-* | Hypothetical membrane spanning protein |  |  |  |
| SAUSA300_1851 | *-* | Hypothetical protein |  |  |  |
| SAUSA300_1852 | *-* | ABC transporter ATP-binding protein |  |  |  |
| SAUSA300_1853 | *-* | Transcriptional regulator |  |  |  |
| SAUSA300_1854 | *recX* | Regulatory protein RecX |  |  |  |
| SAUSA300_1855 | *sgtB* | Monofunctional biosynthetic peptidoglycan transglycosylase (EC 2.4.2.-) |  |  |  |
| SAUSA300_1856 | *-* | PROTEASE I (EC 3.4.-.-) |  |  |  |
| SAUSA300_1857 | *-* | Hypothetical protein |  |  |  |
| SAUSA300_1858 | *-* | Thioredoxin-like oxidoreductases |  |  |  |
| SAUSA300_1859 | *-* | Acyl-CoA hydrolase (EC 3.1.2.20) |  |  |  |
| SAUSA300_1860 | *pepS* | Aminopeptidase (EC 3.4.11.-) |  |  |  |
| SAUSA300_1861 | *-* | Hypothetical protein |  |  |  |
| SAUSA300_1862 | *-* | Protein tyrosine phosphatase (EC 3.1.3.48) |  |  |  |
| SAUSA300_1863 | *-* | Hypothetical protein |  |  |  |
| SAUSA300_1864 | *-* | Ribonuclease BN (EC 3.1.-.-) |  |  |  |
| SAUSA300_1865 | *vraR* | Two-component response regulator YvqC |  |  |  |
| SAUSA300_1866 | *vraS* | Two-component sensor protein yvqE (EC 2.7.3.-) |  |  |  |
| SAUSA300_1867 | *-* | Transporter yvqF |  |  |  |
| SAUSA300_1868 | *-* | Hypothetical protein |  |  |  |
| SAUSA300_1869 | *map* | Methionine aminopeptidase (EC 3.4.11.18) |  |  |  |
| SAUSA300_1870 | *-* | Integral membrane protein |  |  |  |
| SAUSA300_1871 | *-* | Hypothetical protein |  |  |  |
| SAUSA300_1872 | *-* | CobB/CobQ-like glutamine amidotransferase domain |  |  |  |
| SAUSA300_1873 | *-* | UDP-N-acetylmuramoylalanyl-D-glutamate--2,6-diaminopimelate ligase (EC 6.3.2.13) |  |  |  |
| SAUSA300_1874 | *-* | Ferritin |  |  |  |
| SAUSA300_1875 | *-* | DNA polymerase III alpha subunit (EC 2.7.7.7) |  |  |  |
| SAUSA300_1876 | *-* | DNA polymerase IV (EC 2.7.7.7) |  |  |  |
| SAUSA300_1877 | *-* | Permease |  |  |  |
| SAUSA300_1878 | *rumA* | 23S rRNA m(5)U 1939 methyltransferase (EC 2.1.1.-) |  |  |  |
| SAUSA300_1879 | *-* | Diacylglycerol kinase family protein |  |  |  |
| SAUSA300_1880 | *gatB* | Aspartyl/glutamyl-tRNA(Asn/Gln) amidotransferase subunit B (EC 6.3.5.-) |  |  |  |
| SAUSA300_1881 | *gatA* | Aspartyl/glutamyl-tRNA(Asn/Gln) amidotransferase subunit A (EC 6.3.5.-) |  |  |  |
| SAUSA300_1882 | *gatC* | Aspartyl/glutamyl-tRNA(Asn/Gln) amidotransferase subunit C (EC 6.3.5.-) |  |  |  |
| SAUSA300_1883 | *putP* | Sodium/proline symporter |  |  |  |
| SAUSA300_1884 | *-* | Lipoprotein (pheromone precursor) |  |  |  |
| SAUSA300_1885 | *ligA* | NAD-dependent DNA ligase (EC 6.5.1.2) |  |  |  |
| SAUSA300_1886 | *pcrA* | DNA helicase II (EC 3.6.1.-) |  |  |  |
| SAUSA300_1887 | *pcrB* | GERANYLGERANYLGLYCERYL DIPHOSPHATE SYNTHASE |  |  |  |
| SAUSA300_1888 | *-* | cytosolic protein containing cobalamin binding site |  |  |  |
| SAUSA300_1889 | *purB* | Adenylosuccinate lyase (EC 4.3.2.2) |  |  |  |
| SAUSA300_1890 | *-* | Staphopain (EC 3.4.22.-) | -7.68 |  | -4.96 |
| SAUSA300_1891 | *-* | Hypothetical cytosolic protein |  |  |  |
| SAUSA300_1892 | *-* | Hypothetical protein |  |  |  |
| SAUSA300_1893 | *nadE* | NH(3)-dependent NAD(+) synthetase (EC 6.3.5.1) |  |  |  |
| SAUSA300_1894 | *-* | Nicotinate phosphoribosyltransferase (EC 2.4.2.11) |  |  |  |
| SAUSA300_1895 | *-* | Nitric-oxide synthase (EC 1.14.13.39) |  |  |  |
| SAUSA300_1896 | *pheA* | Prephenate dehydratase (EC 4.2.1.51) |  |  |  |
| SAUSA300_1897 | *-* | Transporter, Divalent Anion:Sodium Symporter family |  |  |  |
| SAUSA300_1898 | *-* | Hypothetical protein |  |  |  |
| SAUSA300_1899 | *-* | Pyrazinamidase (EC 3.5.1.-) / Nicotinamidase (EC 3.5.1.19) |  |  |  |
| SAUSA300_1900 | *ppaC* | Inorganic pyrophosphatase (EC 3.6.1.1) |  |  |  |
| SAUSA300_1901 | *aldA2* | Aldehyde dehydrogenase (NAD(P)+) (EC 1.2.1.5) |  |  |  |
| SAUSA300_1902 | *-* | 6-phosphogluconolactonase (EC 3.1.1.31) |  |  |  |
| SAUSA300_1903 | *-* | Hypothetical protein |  |  | 2.38 |
| SAUSA300_1904 | *-* | Hypothetical protein |  |  |  |
| SAUSA300_1905 | *-* | Choloylglycine hydrolase |  |  |  |
| SAUSA300_1906 | *-* | Hypothetical protein |  |  |  |
| SAUSA300_1907 | *-* | Hypothetical protein |  |  |  |
| SAUSA300_1908 | *-* | Hypothetical protein |  |  |  |
| SAUSA300_1909 | *-* | Thioredoxin |  |  |  |
| SAUSA300_1910 | *-* | Hypothetical protein |  |  |  |
| SAUSA300_1911 | *-* | ABC transporter ATP-binding protein |  |  |  |
| SAUSA300_1912 | *-* | Hypothetical protein |  |  |  |
| SAUSA300_1913 | *-* | ABC transporter ATP-binding protein |  |  | -2.04 |
| SAUSA300_1914 | *-* | Transcriptional regulator, GntR family |  |  | -2.11 |
| SAUSA300_1915 | *-* | Hypothetical protein |  |  |  |
| SAUSA300_1916 | *-* | Aspartate aminotransferase (EC 2.6.1.1) |  |  |  |
| SAUSA300_1918 | *-* | Sphingomyelin phosphodiesterase (EC 3.1.4.12) | -3.13 |  | -4.34 |
| SAUSA300_1919 | *-* | Hypothetical protein | -3.34 |  | -2.55 |
| SAUSA300_1920 | *chs* | Chemotaxis-inhibiting protein CHIPS |  |  |  |
| SAUSA300_1921 | *-* | N-acetylmuramoyl-L-alanine amidase (EC 3.5.1.28) |  |  | 2.25 |
| SAUSA300_1922 | *sak* | Staphylokinase precursor |  |  |  |
| SAUSA300_1923 | *-* | CHAP domain containing protein |  | -2.93 | 2.31 |
| SAUSA300_1924 | *-* | Holin |  | -3.35 | 2.15 |
| SAUSA300_1925 | *-* | Phage protein |  | -3.43 |  |
| SAUSA300_1926 | *-* | Hypothetical protein |  |  |  |
| SAUSA300_1927 | *-* | Hypothetical protein |  |  |  |
| SAUSA300_1928 | *-* | Phage-related protein |  |  |  |
| SAUSA300_1929 | *-* | Phage-related protein | -2.84 | -2.92 |  |
| SAUSA300_1930 | *-* | Hypothetical protein | -2.06 | -2.43 |  |
| SAUSA300_1931 | *-* | Hypothetical protein | -2.77 | -3.70 |  |
| SAUSA300_1932 | *-* | Hypothetical protein | -2.29 | -2.62 |  |
| SAUSA300_1933 | *-* | Hypothetical protein | -2.87 | -3.34 |  |
| SAUSA300_1934 | *-* | prophage pi2 protein 39 | -2.65 | -2.38 |  |
| SAUSA300_1935 | *-* | Hypothetical cytosolic protein | -2.80 | -3.15 |  |
| SAUSA300_1936 | *-* | Phage protein | -2.66 | -2.83 |  |
| SAUSA300_1937 | *-* | Phage protein | -2.54 | -2.56 |  |
| SAUSA300_1938 | *-* | Major capsid protein | -2.57 | -2.26 |  |
| SAUSA300_1939 | *-* | ATP-dependent endopeptidase clp proteolytic subunit ClpP (EC 3.4.21.92) | -2.04 | -2.27 |  |
| SAUSA300_1940 | *-* | Portal protein | -2.01 | -2.34 |  |
| SAUSA300_1941 | *-* | Terminase large subunit |  | -2.13 |  |
| SAUSA300_1942 | *-* | Phage-related protein | -2.25 |  |  |
| SAUSA300_1943 | *-* | HNH endonuclease family protein | -2.80 | -3.28 |  |
| SAUSA300_1944 | *-* | Hypothetical protein |  |  |  |
| SAUSA300_1945 | *-* | Phage protein |  |  |  |
| SAUSA300_1946 | *-* | Transcriptional activator rinB |  |  |  |
| SAUSA300_1947 | *-* | Hypothetical protein |  |  |  |
| SAUSA300_1949 | *dut* | Deoxyuridine 5'-triphosphate nucleotidohydrolase (EC 3.6.1.23) |  |  |  |
| SAUSA300_1950 | *-* | Hypothetical protein |  | -2.10 |  |
| SAUSA300_1951 | *-* | Phage protein |  |  |  |
| SAUSA300_1952 | *-* | Hypothetical protein |  | -2.17 |  |
| SAUSA300_1953 | *-* | Hypothetical protein |  | -2.19 |  |
| SAUSA300_1954 | *-* | Phage protein |  | -2.24 |  |
| SAUSA300_1955 | *-* | Phage protein |  | -2.31 |  |
| SAUSA300_1956 | *-* | Hypothetical protein |  |  |  |
| SAUSA300_1957 | *-* | Phage protein |  |  |  |
| SAUSA300_1958 | *-* | Single-strand DNA binding protein |  | -2.23 |  |
| SAUSA300_1959 | *-* | Metal-dependent hydrolase (EC 3.-.-.-) |  | -2.52 |  |
| SAUSA300_1960 | *-* | RecT protein | -2.21 | -2.29 |  |
| SAUSA300_1961 | *-* | Hypothetical protein | -2.20 | -2.07 |  |
| SAUSA300_1962 | *-* | Hypothetical cytosolic protein |  | -2.45 |  |
| SAUSA300_1963 | *-* | Hypothetical cytosolic protein | -2.34 | -2.32 |  |
| SAUSA300_1964 | *-* | Hypothetical protein | -2.17 | -2.43 |  |
| SAUSA300_1966 | *-* | Phage antirepressor protein | -2.55 | -2.14 |  |
| SAUSA300_1967 | *-* | Phage protein |  |  |  |
| SAUSA300_1968 | *-* | Phage transcriptional regulator, Cro/CI family |  |  |  |
| SAUSA300_1969 | *-* | Phage transcriptional repressor |  |  |  |
| SAUSA300_1970 | *-* | DNA polymerase III alpha subunit (EC 2.7.7.7) |  |  |  |
| SAUSA300_1971 | *-* | Phage protein |  |  |  |
| SAUSA300_1972 | *int* | DNA integration/recombination/inversion protein |  |  |  |
| SAUSA300_1973 | *-* | Sphingomyelin phosphodiesterase (EC 3.1.4.12) |  |  |  |
| SAUSA300_1974 | *lukG* | Leukocidin F subunit | -2.89 |  | -2.99 |
| SAUSA300_1975 | *lukH* | Leukocidin S subunit | -2.51 |  | -2.68 |
| SAUSA300_1976 | *-* | Succinyl-diaminopimelate desuccinylase (EC 3.5.1.18) |  |  |  |
| SAUSA300_1977 | *-* | Tetracenomycin polyketide synthesis O-methyltransferase tcmP (EC 2.1.1.-) |  |  |  |
| SAUSA300_1978 | *-* | Ferrichrome-binding protein / Ferrioxamine B binding protein |  |  |  |
| SAUSA300_1979 | *-* | Potassium uptake protein KtrB |  |  |  |
| SAUSA300_1980 | *-* | Acetyltransferase (EC 2.3.1.-) |  |  |  |
| SAUSA300_1981 | *-* | Phage Terminase Small Subunit |  |  |  |
| SAUSA300_1982 | *groEL* | 60 kDa chaperonin GroeL |  |  |  |
| SAUSA300_1983 | *groES* | 10 kDa chaperonin GroeS |  |  |  |
| SAUSA300_1984 | *-* | CAAX amino terminal protease family |  |  |  |
| SAUSA300_1985 | *sdrH* | Hypothetical membrane associated protein |  |  |  |
| SAUSA300_1986 | *-* | Nitroreductase family protein |  |  |  |
| SAUSA300_1987 | *-* | Beta-ureidopropionase (EC 3.5.1.6) |  |  |  |
| SAUSA300_1988 | *hld* | Delta-lysin |  |  | -908.28 |
| SAUSA300_1989 | *agrB* | Accessory gene regulator protein B AgrB |  |  | -3564.35 |
| SAUSA300_1990 | *agrD* | Autoinducing peptide AgrD |  |  | -2238.25 |
| SAUSA300_1991 | *agrC* | Sensory transduction histidine kinase AgrC (EC 2.7.3.-) |  |  | -8947.39 |
| SAUSA300_1992 | *agrA* | Accessory gene regulator protein A AgrA |  |  | -2702.90 |
| SAUSA300_1993 | *-* | Fructokinase (EC 2.7.1.4) |  |  |  |
| SAUSA300_1994 | *scrB* | Sucrose-6-phosphate hydrolase (EC 3.2.1.26) |  |  |  |
| SAUSA300_1995 | *scrR* | Sucrose operon repressor | -2.16 |  |  |
| SAUSA300_1996 | *amt* | Hypothetical protein |  |  |  |
| SAUSA300_1997 | *-* | Transcriptional regulatory protein |  |  |  |
| SAUSA300_1998 | *-* | putative transport system permease protein |  |  |  |
| SAUSA300_1999 | *rex* | Redox-sensitive transcriptional regulator Rex |  |  |  |
| SAUSA300_2000 | *vga* | ABC transporter ATP-binding protein |  |  |  |
| SAUSA300_2001 | *-* | DNA mismatch repair protein MutS |  |  |  |
| SAUSA300_2002 | *-* | O-sialoglycoprotein endopeptidase (EC 3.4.24.57) |  |  |  |
| SAUSA300_2003 | *rimI* | Ribosomal-protein-S18-alanine acetyltransferase (EC 2.3.1.128) |  |  |  |
| SAUSA300_2004 | *-* | Non-proteolytic protein, peptidase family M22 |  |  |  |
| SAUSA300_2005 | *-* | ATP/GTP hydrolase |  |  |  |
| SAUSA300_2006 | *ilvD* | Dihydroxy-acid dehydratase (EC 4.2.1.9) |  |  |  |
| SAUSA300_2007 | *ilvB* | Acetolactate synthase large subunit (EC 2.2.1.6) |  |  |  |
| SAUSA300_2008 | *ilvN* | Acetolactate synthase small subunit (EC 2.2.1.6) |  |  |  |
| SAUSA300_2009 | *ilvC* | Ketol-acid reductoisomerase (EC 1.1.1.86) / 2-dehydropantoate 2-reductase (EC 1.1.1.169) |  |  |  |
| SAUSA300_2010 | *leuA* | 2-isopropylmalate synthase (EC 2.3.3.13) |  |  |  |
| SAUSA300_2011 | *leuB* | 3-isopropylmalate dehydrogenase (EC 1.1.1.85) |  |  |  |
| SAUSA300_2012 | *leuC* | 3-isopropylmalate dehydratase large subunit (EC 4.2.1.33) |  |  |  |
| SAUSA300_2013 | *leuD* | 3-isopropylmalate dehydratase small subunit (EC 4.2.1.33) |  |  |  |
| SAUSA300_2014 | *ilvA* | Threonine dehydratase (EC 4.3.1.19) |  |  |  |
| SAUSA300_2020 | *-* | Metallopeptidase, SprT family (EC 3.4.24.-) |  |  |  |
| SAUSA300_2021 | *-* | Transcription accessory protein (S1 RNA binding domain) |  |  |  |
| SAUSA300_2022 | *rpoF* | RNA polymerase sigma-B factor |  |  |  |
| SAUSA300_2023 | *rsbW* | Anti-sigma B factor |  |  |  |
| SAUSA300_2024 | *rsbV* | Anti-sigma B factor antagonist |  |  |  |
| SAUSA300_2025 | *rsbU* | Sigma factor sigB regulation protein rsbU |  |  |  |
| SAUSA300_2026 | *-* | PEMK-like protein |  |  |  |
| SAUSA300_2027 | *alr* | Alanine racemase (EC 5.1.1.1) |  |  |  |
| SAUSA300_2028 | *acpS* | Holo-[acyl-carrier protein] synthase (EC 2.7.8.7) |  |  |  |
| SAUSA300_2029 | *-* | Hypothetical membrane spanning protein |  |  |  |
| SAUSA300_2030 | *-* | Hypothetical membrane spanning protein |  |  |  |
| SAUSA300_2031 | *-* | Hypothetical protein |  |  |  |
| SAUSA300_2032 | *kdpC* | Potassium-transporting ATPase C chain (EC 3.6.3.12) | -3.26 |  | -3.93 |
| SAUSA300_2033 | *kdpB* | Potassium-transporting ATPase B chain (EC 3.6.3.12) | -3.00 |  | -5.75 |
| SAUSA300_2034 | *kdpA* | Potassium-transporting ATPase A chain (EC 3.6.3.12) | -4.59 |  | -12.85 |
| SAUSA300_2035 | *kdpD* | Sensor protein KdpD (EC 2.7.3.-) | -2.91 |  | -12.01 |
| SAUSA300_2036 | *kdpE* | Two-component response regulator kdpE | -3.08 |  | -8.36 |
| SAUSA300_2037 | *-* | ATP-dependent RNA helicase |  |  |  |
| SAUSA300_2038 | *murF* | UDP-N-acetylmuramoyl-tripeptide--D-alanyl-D-alanine ligase (EC 6.3.2.10) |  |  |  |
| SAUSA300_2039 | *ddl* | D-alanine--D-alanine ligase (EC 6.3.2.4) |  |  |  |
| SAUSA300_2040 | *-* | Rod shape-determining protein rodA |  |  |  |
| SAUSA300_2041 | *-* | Hypothetical protein |  |  |  |
| SAUSA300_2042 | *-* | Hypothetical protein |  |  |  |
| SAUSA300_2043 | *-* | Hypothetical cytosolic protein | 2.36 |  |  |
| SAUSA300_2044 | *cls* | Cardiolipin synthetase (EC 2.7.8.-) |  |  |  |
| SAUSA300_2045 | *-* | Metal dependent hydrolase |  |  |  |
| SAUSA300_2046 | *oxaA* | 60 kDa inner membrane protein YidC |  |  |  |
| SAUSA300_2047 | *thiE* | Thiamin-phosphate pyrophosphorylase (EC 2.5.1.3) |  |  |  |
| SAUSA300_2048 | *thiM* | Hydroxyethylthiazole kinase (EC 2.7.1.50) |  |  |  |
| SAUSA300_2049 | *thiD* | Phosphomethylpyrimidine kinase (EC 2.7.4.7) / Hydroxymethylpyrimidine kinase (EC 2.7.1.49) |  |  |  |
| SAUSA300_2050 | *-* | Transcriptional activator tenA |  |  |  |
| SAUSA300_2051 | *-* | SceD precursor |  | 3.69 | -3.08 |
| SAUSA300_2052 | *-* | Single-strand DNA binding protein | -3.89 |  | -2.75 |
| SAUSA300_2053 | *-* | Hypothetical protein |  |  |  |
| SAUSA300_2054 | *fabZ* | (3R)-hydroxyacyl-[acyl carrier protein] dehydratase (EC 4.2.1.60) |  |  |  |
| SAUSA300_2055 | *murA* | UDP-N-acetylglucosamine 1-carboxyvinyltransferase (EC 2.5.1.7) |  |  |  |
| SAUSA300_2056 | *-* | Hypothetical membrane associated protein |  |  |  |
| SAUSA300_2057 | *atpC* | ATP synthase epsilon chain (EC 3.6.3.14) |  |  |  |
| SAUSA300_2058 | *atpD* | ATP synthase beta chain (EC 3.6.3.14) |  |  |  |
| SAUSA300_2059 | *atpG* | ATP synthase gamma chain (EC 3.6.3.14) |  |  |  |
| SAUSA300_2060 | *atpA* | ATP synthase alpha chain (EC 3.6.3.14) |  |  |  |
| SAUSA300_2061 | *atpH* | ATP synthase delta chain (EC 3.6.3.14) |  |  |  |
| SAUSA300_2062 | *atpF* | ATP synthase B chain (EC 3.6.3.14) |  |  |  |
| SAUSA300_2063 | *atpE* | ATP synthase C chain (EC 3.6.3.14) |  |  |  |
| SAUSA300_2064 | *atpB* | ATP synthase A chain (EC 3.6.3.14) |  |  |  |
| SAUSA300_2065 | *-* | UDP-N-acetylglucosamine 2-epimerase (EC 5.1.3.14) |  |  |  |
| SAUSA300_2066 | *upp* | Uracil phosphoribosyltransferase (EC 2.4.2.9) |  |  |  |
| SAUSA300_2067 | *glyA* | Serine hydroxymethyltransferase (EC 2.1.2.1) |  |  |  |
| SAUSA300_2068 | *-* | Hypothetical protein |  |  |  |
| SAUSA300_2069 | *-* | Protein tyrosine phosphatase (EC 3.1.3.48) |  |  |  |
| SAUSA300_2070 | *-* | Sua5/YciO/YrdC/YwlC family protein |  |  |  |
| SAUSA300_2071 | *-* | Peptide release factor-glutamine N5-methyltransferase (EC 2.1.1.-) |  |  |  |
| SAUSA300_2072 | *prfA* | Bacterial peptide chain release factor 1 (RF-1) |  |  |  |
| SAUSA300_2073 | *tdk* | Thymidine kinase (EC 2.7.1.21) |  |  |  |
| SAUSA300_2074 | *rpmE2* | LSU ribosomal protein L31P |  |  |  |
| SAUSA300_2075 | *rho* | Transcription termination factor rho | -2.54 |  |  |
| SAUSA300_2076 | *-* | Aldehyde dehydrogenase (EC 1.2.1.3) |  |  |  |
| SAUSA300_2077 | *-* | Transcriptional regulator, MarR family |  |  |  |
| SAUSA300_2078 | *murA* | UDP-N-acetylglucosamine 1-carboxyvinyltransferase (EC 2.5.1.7) |  |  |  |
| SAUSA300_2079 | *fba* | Fructose-bisphosphate aldolase (EC 4.1.2.13) |  |  |  |
| SAUSA300_2080 | *-* | Hypothetical protein |  |  |  |
| SAUSA300_2081 | *pyrG* | CTP synthase (EC 6.3.4.2) |  |  |  |
| SAUSA300_2082 | *rpoE* | DNA-directed RNA polymerase delta chain (EC 2.7.7.6) |  |  |  |
| SAUSA300_2083 | *-* | Acetyltransferase (EC 2.3.1.-) |  |  |  |
| SAUSA300_2084 | *coaA* | Pantothenate kinase (EC 2.7.1.33) |  |  |  |
| SAUSA300_2085 | *-* | Hypothetical protein |  |  |  |
| SAUSA300_2086 | *-* | Hypothetical cytosolic protein |  |  |  |
| SAUSA300_2087 | *-* | Peptidase family M20/M25/M40 |  |  |  |
| SAUSA300_2088 | *luxS* | Hypothetical protein |  |  |  |
| SAUSA300_2089 | *pdp* | Thymidine phosphorylase (EC 2.4.2.4) |  |  |  |
| SAUSA300_2091 | *deoD* | Purine nucleoside phosphorylase (EC 2.4.2.1) |  |  |  |
| SAUSA300_2092 | *dps* | Non-specific DNA-binding protein Dps / Iron-binding ferritin-like antioxidant protein / Ferroxidase (EC 1.16.3.1) |  |  |  |
| SAUSA300_2093 | *-* | Hypothetical protein |  |  |  |
| SAUSA300_2094 | *-* | Hypothetical protein |  |  |  |
| SAUSA300_2095 | *-* | Hypothetical protein |  |  |  |
| SAUSA300_2096 | *manA* | Mannose-6-phosphate isomerase (EC 5.3.1.8) |  |  |  |
| SAUSA300_2097 | *-* | Putative NAD-dependent dehydrogenase |  |  |  |
| SAUSA300_2098 | *arsR* | Hypothetical protein |  |  |  |
| SAUSA300_2099 | *-* | Cobalt-zinc-cadmium resistance protein CzcD |  |  |  |
| SAUSA300_2100 | *-* | Lytic regulatory protein |  |  |  |
| SAUSA300_2101 | *-* | Hypothetical protein |  |  |  |
| SAUSA300_2102 | *-* | Hypothetical cytosolic protein |  |  |  |
| SAUSA300_2103 | *-* | Hypothetical protein |  |  |  |
| SAUSA300_2104 | *glmS* | Glucosamine--fructose-6-phosphate aminotransferase [isomerizing] (EC 2.6.1.16) |  |  |  |
| SAUSA300_2105 | *mtlF* | PTS system, mannitol-specific IIBC component (EC 2.7.1.69) | 4.07 | 2.73 |  |
| SAUSA300_2106 | *-* | Transcription antiterminator, BglG family / PTS system, mannitol (Cryptic)-specific IIA component (EC 2.7.1.69) | 4.02 | 2.94 |  |
| SAUSA300_2107 | *mtlA* | PTS system, mannitol-specific IIA component (EC 2.7.1.69) | 2.31 | 2.15 |  |
| SAUSA300_2108 | *mtlD* | Mannitol-1-phosphate 5-dehydrogenase (EC 1.1.1.17) | 2.29 | 2.39 |  |
| SAUSA300_2109 | *fmtB* | Hypothetical protein |  |  |  |
| SAUSA300_2110 | *fmtB* | Methicillin resistance protein |  |  |  |
| SAUSA300_2111 | *glmM* | Phosphoglucosamine mutase (EC 5.4.2.10) |  |  |  |
| SAUSA300_2112 | *-* | Hypothetical membrane associated protein |  |  |  |
| SAUSA300_2113 | *-* | Hypothetical membrane spanning protein |  |  |  |
| SAUSA300_2114 | *rocF* | Arginase (EC 3.5.3.1) | -2.28 |  |  |
| SAUSA300_2125 | *-* | Iron-sulfur cluster assembly/repair protein ApbC |  |  |  |
| SAUSA300_2126 | *-* | Multidrug resistance protein B |  |  |  |
| SAUSA300_2127 | *-* | Hypothetical protein |  |  |  |
| SAUSA300_2128 | *-* | Permease |  |  |  |
| SAUSA300_2129 | *-* | Conserved membrane protein (hemolysin III homolog) |  |  |  |
| SAUSA300_2130 | *-* | UDP-N-acetylglucosamine pyrophosphorylase (EC 2.7.7.23) |  |  |  |
| SAUSA300_2131 | *-* | Predicted membrane-bound metal-dependent hydrolase |  |  |  |
| SAUSA300_2132 | *-* | Hypothetical cytosolic protein |  |  |  |
| SAUSA300_2133 | *-* | Hypothetical membrane spanning protein |  |  |  |
| SAUSA300_2134 | *htsC* | Iron(III) dicitrate transport system permease protein |  |  |  |
| SAUSA300_2135 | *htsB* | Ferrichrome transport system permease protein |  |  |  |
| SAUSA300_2136 | *htsA* | Iron(III) dicitrate-binding protein |  |  |  |
| SAUSA300_2137 | *-* | Hypothetical cytosolic protein |  |  |  |
| SAUSA300_2138 | *-* | Amino-acid citrate synthetase (EC 6.-.-.-) |  |  |  |
| SAUSA300_2139 | *-* | Macrolide-efflux protein |  |  |  |
| SAUSA300_2140 | *-* | Siderophore synthase (EC 6.-.-.-) |  |  |  |
| SAUSA300_2142 | *asp23* | Alkaline shock protein |  |  |  |
| SAUSA300_2143 | *-* | Small integral membrane protein |  |  |  |
| SAUSA300_2144 | *-* | Hypothetical protein |  |  |  |
| SAUSA300_2145 | *-* | Glycine betaine transporter |  |  |  |
| SAUSA300_2146 | *-* | Hypothetical protein |  |  |  |
| SAUSA300_2147 | *-* | Quinone oxidoreductase (EC 1.6.5.5) |  |  |  |
| SAUSA300_2148 | *-* | putative acyltransferases and hydrolases with the alpha/beta hydrolase fold |  |  |  |
| SAUSA300_2149 | *lacG* | 6-phospho-beta-galactosidase (EC 3.2.1.85) |  |  |  |
| SAUSA300_2150 | *lacE* | PTS system, lactose-specific IIBC component (EC 2.7.1.69) |  |  |  |
| SAUSA300_2151 | *lacF* | PTS system, lactose-specific IIA component (EC 2.7.1.69) |  |  |  |
| SAUSA300_2152 | *lacD* | Tagatose-bisphosphate aldolase (EC 4.1.2.40) |  |  |  |
| SAUSA300_2153 | *lacC* | Tagatose-6-phosphate kinase (EC 2.7.1.144) |  |  |  |
| SAUSA300_2154 | *lacB* | Galactose-6-phosphate isomerase lacB subunit (EC 5.3.1.26) |  |  |  |
| SAUSA300_2155 | *lacA* | Galactose-6-phosphate isomerase lacA subunit (EC 5.3.1.26) |  |  |  |
| SAUSA300_2156 | *-* | Lactose phosphotransferase system repressor |  |  |  |
| SAUSA300_2157 | *-* | Sir2 family protein |  |  |  |
| SAUSA300_2158 | *-* | Hypothetical protein |  |  |  |
| SAUSA300_2159 | *-* | Morphine 6-dehydrogenase (EC 1.1.1.218) |  |  |  |
| SAUSA300_2160 | *-* | Transcriptional regulator, MerR family |  |  |  |
| SAUSA300_2161 | *hysA* | Hyaluronate lyase precursor (EC 4.2.2.1) |  |  |  |
| SAUSA300_2162 | *-* | Peptidoglycan-specific endopeptidase, M23 family |  |  |  |
| SAUSA300_2163 | *-* | Hydrolase (HAD superfamily) |  |  |  |
| SAUSA300_2164 | *-* | Outer membrane protein | -2.25 | 2.19 | -13.03 |
| SAUSA300_2165 | *budA* | Alpha-acetolactate decarboxylase (EC 4.1.1.5) |  |  |  |
| SAUSA300_2166 | *alsS* | Acetolactate synthase (EC 2.2.1.6) |  |  |  |
| SAUSA300_2167 | *-* | Hypothetical protein |  |  |  |
| SAUSA300_2168 | *-* | ATPases with chaperone activity, ATP-binding domain |  |  |  |
| SAUSA300_2169 | *-* | Hypothetical protein |  |  |  |
| SAUSA300_2171 | *rpsI* | Ssu ribosomal protein S9P |  |  |  |
| SAUSA300_2172 | *rplM* | Lsu ribosomal protein L13P |  |  |  |
| SAUSA300_2173 | *truA* | tRNA pseudouridine synthase A (EC 4.2.1.70) |  |  |  |
| SAUSA300_2174 | *-* | Cobalt transport protein cbiQ |  |  |  |
| SAUSA300_2175 | *cbiO* | Cobalt transport ATP-binding protein cbiO |  |  |  |
| SAUSA300_2176 | *cbiO* | Cobalt transport ATP-binding protein cbiO |  |  |  |
| SAUSA300_2177 | *rplQ* | Lsu ribosomal protein L17P |  |  |  |
| SAUSA300_2178 | *rpoA* | DNA-directed RNA polymerase alpha chain (EC 2.7.7.6) |  |  |  |
| SAUSA300_2179 | *rpsK* | SSU ribosomal protein S11P |  |  |  |
| SAUSA300_2180 | *rpsM* | Ssu ribosomal protein S13P |  |  |  |
| SAUSA300_2181 | *rpmJ* | Lsu ribosomal protein L36P |  |  |  |
| SAUSA300_2182 | *infA* | Bacterial protein translation initiation factor 1 (IF-1) |  |  |  |
| SAUSA300_2183 | *adk* | Adenylate kinase (EC 2.7.4.3) / Nucleoside-diphosphate kinase (EC 2.7.4.6) |  |  |  |
| SAUSA300_2184 | *secY* | Protein translocase subunit secY |  |  |  |
| SAUSA300_2185 | *rplO* | Lsu ribosomal protein L15P |  |  |  |
| SAUSA300_2186 | *rpmD* | LSU ribosomal protein L30P |  |  |  |
| SAUSA300_2187 | *rpsE* | Ssu ribosomal protein S5P |  |  |  |
| SAUSA300_2188 | *rplR* | Lsu ribosomal protein L18P |  |  |  |
| SAUSA300_2189 | *rplF* | LSU ribosomal protein L6P |  |  |  |
| SAUSA300_2190 | *rpsH* | Ssu ribosomal protein S8P |  |  |  |
| SAUSA300_2191 | *rpsN* | Ssu ribosomal protein S14P |  |  |  |
| SAUSA300_2192 | *rplE* | Lsu ribosomal protein L5P |  |  |  |
| SAUSA300_2193 | *rplX* | Lsu ribosomal protein L24P |  |  | 2.25 |
| SAUSA300_2194 | *rplN* | Lsu ribosomal protein L14P |  |  | 2.12 |
| SAUSA300_2195 | *rpsQ* | Ssu ribosomal protein S17P |  |  | 2.07 |
| SAUSA300_2196 | *rpmC* | Lsu ribosomal protein L29P |  |  | 2.13 |
| SAUSA300_2197 | *rplP* | Lsu ribosomal protein L16P |  |  | 2.00 |
| SAUSA300_2198 | *rpsC* | Ssu ribosomal protein S3P |  |  |  |
| SAUSA300_2199 | *rplV* | Lsu ribosomal protein L22P |  |  |  |
| SAUSA300_2200 | *rpsS* | Ssu ribosomal protein S19P |  |  |  |
| SAUSA300_2201 | *rplB* | LSU ribosomal protein L2P |  |  |  |
| SAUSA300_2202 | *rplW* | Lsu ribosomal protein L23P |  |  |  |
| SAUSA300_2203 | *rplD* | Lsu ribosomal protein L1E (= L4P) |  |  |  |
| SAUSA300_2204 | *rplC* | Lsu ribosomal protein L3P |  |  |  |
| SAUSA300_2205 | *rpsJ* | Ssu ribosomal protein S10P |  |  |  |
| SAUSA300_2206 | *-* | Hypothetical protein |  |  |  |
| SAUSA300_2207 | *-* | Guanine-hypoxanthine permease |  |  |  |
| SAUSA300_2208 | *topB* | DNA topoisomerase III (EC 5.99.1.2) |  |  |  |
| SAUSA300_2209 | *-* | Hypothetical protein |  |  |  |
| SAUSA300_2210 | *glcU* | Glucose uptake protein |  |  |  |
| SAUSA300_2211 | *-* | Permease |  |  |  |
| SAUSA300_2212 | *-* | ATPase involved in DNA repair/chromosome segregation |  |  |  |
| SAUSA300_2213 | *-* | Acriflavin resistance plasma membrane protein |  |  |  |
| SAUSA300_2214 | *-* | UDP-N-acetylmuramoylpentapeptide-lysine N(6)-glycyltransferase (EC 2.3.2.-) |  |  |  |
| SAUSA300_2215 | *-* | Hypothetical cytosolic protein |  |  |  |
| SAUSA300_2216 | *-* | Transcriptional regulator, MarR family |  |  |  |
| SAUSA300_2217 | *-* | Transporter, MFS superfamily |  |  |  |
| SAUSA300_2218 | *-* | STAPHYLOCOCCAL ACCESSORY REGULATOR A |  |  |  |
| SAUSA300_2219 | *moaA* | Molybdenum cofactor biosynthesis protein A |  |  |  |
| SAUSA300_2220 | *mobA* | Molybdopterin-guanine dinucleotide biosynthesis protein A |  |  |  |
| SAUSA300_2221 | *moaD* | Molybdopterin converting factor, small subunit |  |  |  |
| SAUSA300_2222 | *moaE* | Molybdopterin converting factor, large subunit |  |  |  |
| SAUSA300_2223 | *mobB* | Molybdopterin-guanine dinucleotide biosynthesis protein B |  |  |  |
| SAUSA300_2224 | *moeA* | Molybdopterin biosynthesis MoeA protein |  |  |  |
| SAUSA300_2225 | *moaC* | Molybdenum cofactor biosynthesis protein C |  |  |  |
| SAUSA300_2226 | *moaB* | Molybdenum cofactor biosynthesis protein B |  |  |  |
| SAUSA300_2227 | *moeB* | Molybdopterin biosynthesis MoeB protein |  |  |  |
| SAUSA300_2228 | *modC* | Molybdenum transport ATP-binding protein modC |  |  |  |
| SAUSA300_2229 | *modB* | Molybdenum transport system permease protein modB |  |  |  |
| SAUSA300_2230 | *modA* | Molybdate-binding protein |  |  |  |
| SAUSA300_2231 | *fdhD* | FdhD protein (FdsC) |  |  |  |
| SAUSA300_2232 | *-* | Acetyltransferase (EC 2.3.1.-) |  |  |  |
| SAUSA300_2233 | *-* | BioY protein |  |  |  |
| SAUSA300_2234 | *-* | Inosine-uridine preferring nucleoside hydrolase (EC 3.2.2.1) |  |  |  |
| SAUSA300_2235 | *-* | Ferrichrome-binding protein / Ferrioxamine B binding protein / Aerobactin binding protein / Coprogen binding protein |  |  |  |
| SAUSA300_2236 | *-* | Acyl-CoA dehydrogenase, short-chain specific (EC 1.3.99.2) |  |  |  |
| SAUSA300_2237 | *-* | Urea transporter | -2.69 |  | -3.77 |
| SAUSA300_2238 | *ureA* | Urease gamma subunit (EC 3.5.1.5) | -2.90 |  | -5.64 |
| SAUSA300_2239 | *ureB* | Urease beta subunit (EC 3.5.1.5) | -3.72 |  | -5.78 |
| SAUSA300_2240 | *ureC* | Urease alpha subunit (EC 3.5.1.5) | -3.08 |  | -4.59 |
| SAUSA300_2241 | *ureE* | Urease accessory protein ureE | -2.27 |  | -2.53 |
| SAUSA300_2242 | *ureF* | Urease accessory protein ureF | -2.42 |  | -3.26 |
| SAUSA300_2243 | *ureG* | Urease accessory protein ureG | -2.08 |  | -2.37 |
| SAUSA300_2244 | *ureD* | Urease accessory protein ureD | -2.82 |  | -3.11 |
| SAUSA300_2245 | *-* | STAPHYLOCOCCAL ACCESSORY REGULATOR A |  |  |  |
| SAUSA300_2246 | *-* | Hypothetical protein |  |  |  |
| SAUSA300_2247 | *-* | Staphylococcal accessory regulator |  | -2.26 |  |
| SAUSA300_2248 | *-* | Transcriptional regulator, AraC family | -2.13 | -2.16 |  |
| SAUSA300_2249 | *ssaA* | Secretory antigen precursor SsaA |  |  |  |
| SAUSA300_2250 | *nhaC* | Na+/H+ antiporter nhaC |  |  |  |
| SAUSA300_2251 | *-* | Opine dehydrogenase (EC 1.5.1.28) |  |  |  |
| SAUSA300_2252 | *-* | Hypothetical protein |  |  |  |
| SAUSA300_2253 | *ssaA* | Secretory antigen precursor SsaA |  |  | -2.55 |
| SAUSA300_2254 | *-* | Glyoxylate reductase (NADP+) (EC 1.1.1.79) / Glyoxylate reductase (NAD+) (EC 1.1.1.26) / Hydroxypyruvate reductase (EC 1.1.1.81) |  |  |  |
| SAUSA300_2255 | *-* | Salicylate hydroxylase (EC 1.14.13.1) |  |  |  |
| SAUSA300_2256 | *-* | Peptidoglycan endo-beta-N-acetylglucosaminidase (EC 3.2.1.-) |  |  |  |
| SAUSA300_2257 | *-* | Hypothetical protein |  |  |  |
| SAUSA300_2258 | *-* | Formate dehydrogenase alpha chain (EC 1.2.1.2) |  |  |  |
| SAUSA300_2259 | *-* | Transcriptional regulator, LytR family |  |  |  |
| SAUSA300_2260 | *-* | Myo-inositol-1(or 4)-monophosphatase (EC 3.1.3.25) |  |  |  |
| SAUSA300_2261 | *-* | Transcriptional regulator, DeoR family |  |  |  |
| SAUSA300_2262 | *-* | Integral membrane protein / CAAX amino terminal protease family |  |  |  |
| SAUSA300_2263 | *-* | Transposase |  |  |  |
| SAUSA300_2264 | *-* | Transcriptional regulator, RpiR family |  |  |  |
| SAUSA300_2265 | *-* | Amino acid permease |  |  |  |
| SAUSA300_2266 | *-* | Hypothetical protein |  |  |  |
| SAUSA300_2267 | *-* | Phosphoglycolate phosphatase (EC 3.1.3.18) |  |  |  |
| SAUSA300_2268 | *-* | Transporter, Sodium/bile acid symporter family |  |  |  |
| SAUSA300_2269 | *-* | Hypothetical protein |  |  |  |
| SAUSA300_2270 | *glvC* | PTS system, maltose and glucose-specific IIBC component (EC 2.7.1.69) |  | 2.81 |  |
| SAUSA300_2271 | *-* | Transcriptional regulator, RpiR family |  |  |  |
| SAUSA300_2272 | *-* | Hypothetical protein |  |  |  |
| SAUSA300_2273 | *-* | Na+/H+ antiporter NhaC |  |  |  |
| SAUSA300_2274 | *-* | Hypothetical membrane spanning protein |  |  |  |
| SAUSA300_2275 | *-* | Hypothetical protein |  |  |  |
| SAUSA300_2276 | *-* | N-acyl-L-amino acid amidohydrolase (EC 3.5.1.14) |  |  |  |
| SAUSA300_2277 | *hutI* | Imidazolonepropionase (EC 3.5.2.7) |  |  |  |
| SAUSA300_2278 | *hutU* | Urocanate hydratase (EC 4.2.1.49) |  |  |  |
| SAUSA300_2279 | *-* | Transcriptional regulators, LysR family |  |  |  |
| SAUSA300_2280 | *fosB* | Fosfomycin resistance protein |  |  |  |
| SAUSA300_2281 | *hutG* | Formiminoglutamase (EC 3.5.3.8) |  |  |  |
| SAUSA300_2282 | *-* | Hypothetical protein |  |  |  |
| SAUSA300_2283 | *rpiA* | Ribose 5-phosphate isomerase (EC 5.3.1.6) |  |  |  |
| SAUSA300_2284 | *-* | Molybdenum cofactor sulphurase |  |  |  |
| SAUSA300_2285 | *galM* | Aldose 1-epimerase (EC 5.1.3.3) |  |  |  |
| SAUSA300_2286 | *-* | Hypothetical membrane associated protein | 2.03 |  | 2.98 |
| SAUSA300_2287 | *-* | Sodium export permease protein |  |  |  |
| SAUSA300_2288 | *-* | Sodium export ATP-binding protein |  |  |  |
| SAUSA300_2289 | *-* | Integral membrane protein |  |  | -2.41 |
| SAUSA300_2290 | *-* | DNA-3-methyladenine glycosylase II (EC 3.2.2.21) |  |  |  |
| SAUSA300_2291 | *gltS* | Sodium/glutamate symport carrier protein |  |  |  |
| SAUSA300_2292 | *fni* | Isopentenyl-diphosphate delta-isomerase (EC 5.3.3.2) |  |  |  |
| SAUSA300_2293 | *corA* | Magnesium and cobalt transport protein corA |  |  |  |
| SAUSA300_2294 | *-* | Hypothetical cytosolic protein |  |  |  |
| SAUSA300_2295 | *-* | Hypothetical protein |  |  |  |
| SAUSA300_2296 | *-* | Esterase (EC 3.1.1.-) |  |  |  |
| SAUSA300_2297 | *-* | Hypothetical protein |  |  |  |
| SAUSA300_2298 | *-* | Multidrug resistance protein B | 5.78 | 6.95 |  |
| SAUSA300_2299 | *-* | Multidrug resistance protein A | 10.84 | 8.45 |  |
| SAUSA300_2300 | *-* | Transcriptional regulator, TetR family |  |  |  |
| SAUSA300_2301 | *tcaB* | Hypothetical protein |  |  |  |
| SAUSA300_2302 | *tcaA* | Teicoplanin-associated protein TcaA |  |  |  |
| SAUSA300_2303 | *tcaR* | TcaR transcription regulator |  |  |  |
| SAUSA300_2304 | *-* | EpiH/GdmH-related protein |  |  |  |
| SAUSA300_2305 | *-* | Transposase |  |  |  |
| SAUSA300_2306 | *-* | ABC transporter ATP-binding protein | 3.52 | 2.05 |  |
| SAUSA300_2307 | *-* | ABC transporter permease protein | 2.65 | 2.26 |  |
| SAUSA300_2308 | *-* | Two-component response regulator |  |  |  |
| SAUSA300_2309 | *-* | Two component system histidine kinase (EC 2.7.3.-) |  |  |  |
| SAUSA300_2310 | *-* | Transcriptional regulator |  |  |  |
| SAUSA300_2311 | *-* | Hypothetical membrane associated protein |  |  |  |
| SAUSA300_2312 | *mqo* | Malate:quinone oxidoreductase (EC 1.1.99.16) |  |  |  |
| SAUSA300_2313 | *-* | L-lactate permease |  |  |  |
| SAUSA300_2314 | *-* | Hypothetical protein |  |  |  |
| SAUSA300_2315 | *-* | membrane lipoprotein |  |  |  |
| SAUSA300_2316 | *-* | Protease synthase and sporulation negative regulatory protein PAI 1 |  |  |  |
| SAUSA300_2317 | *-* | Alcohol dehydrogenase (EC 1.1.1.1) |  |  |  |
| SAUSA300_2318 | *-* | Acetyltransferase, GNAT family |  |  |  |
| SAUSA300_2319 | *-* | Thioredoxin reductase (EC 1.8.1.9) | -2.03 |  |  |
| SAUSA300_2320 | *-* | Hypothetical protein |  |  |  |
| SAUSA300_2321 | *-* | Phage infection protein | 30.75 | 30.51 |  |
| SAUSA300_2322 | *-* | Transcriptional regulator, TetR family |  |  |  |
| SAUSA300_2323 | *cobI* | Magnesium and cobalt transport protein corA |  |  |  |
| SAUSA300_2324 | *-* | PTS system, sucrose-specific IIBC component (EC 2.7.1.69) |  | 2.10 |  |
| SAUSA300_2325 | *-* | Hypothetical protein | -2.81 |  |  |
| SAUSA300_2326 | *-* | Transcriptional regulator, AraC family |  |  |  |
| SAUSA300_2327 | *-* | General stress protein 26 |  |  |  |
| SAUSA300_2328 | *-* | Hypothetical protein |  |  |  |
| SAUSA300_2329 | *gltT* | Proton/sodium-glutamate symport protein |  |  |  |
| SAUSA300_2330 | *-* | Hypothetical protein |  |  | 2.25 |
| SAUSA300_2331 | *-* | Transcriptional regulator, MarR family |  |  |  |
| SAUSA300_2332 | *-* | Small heat shock protein |  |  |  |
| SAUSA300_2333 | *narK* | Nitrite extrusion protein |  | -3.08 | 2.40 |
| SAUSA300_2334 | *-* | Hypothetical cytosolic protein |  |  |  |
| SAUSA300_2335 | *-* | Hypothetical cytosolic protein |  |  |  |
| SAUSA300_2336 | *-* | Transcriptional activator tipA |  |  |  |
| SAUSA300_2337 | *-* | Two-component response regulator |  |  |  |
| SAUSA300_2338 | *-* | Two component system histidine kinase (EC 2.7.3.-) |  |  |  |
| SAUSA300_2339 | *-* | Transcriptional regulator |  |  |  |
| SAUSA300_2340 | *narI* | Respiratory nitrate reductase gamma chain (EC 1.7.99.4) |  |  |  |
| SAUSA300_2341 | *narJ* | Respiratory nitrate reductase delta chain (EC 1.7.99.4) |  | -3.45 | 2.86 |
| SAUSA300_2342 | *narH* | Respiratory nitrate reductase beta chain (EC 1.7.99.4) |  | -3.10 | 2.66 |
| SAUSA300_2343 | *-* | Respiratory nitrate reductase alpha chain (EC 1.7.99.4) |  | -3.70 | 3.87 |
| SAUSA300_2344 | *-* | Uroporphyrin-III C-methyltransferase (EC 2.1.1.107) |  | -2.41 | 2.44 |
| SAUSA300_2345 | *nirD* | Nitrite reductase [NAD(P)H] small subunit (EC 1.7.1.4) |  | -2.34 |  |
| SAUSA300_2346 | *nirB* | Nitrite reductase [NAD(P)H] large subunit (EC 1.7.1.4) |  |  |  |
| SAUSA300_2347 | *nirR* | Sirohydrochlorin cobaltochelatase (EC 4.99.1.3) |  |  | 2.05 |
| SAUSA300_2348 | *-* | Acetyltransferase (EC 2.3.1.-) |  |  |  |
| SAUSA300_2349 | *-* | Nitrite transporter |  |  |  |
| SAUSA300_2350 | *-* | Hypothetical cytosolic protein |  |  |  |
| SAUSA300_2351 | *-* | Hypothetical protein | 2.18 |  |  |
| SAUSA300_2352 | *-* | RelE protein |  |  |  |
| SAUSA300_2353 | *-* | RelB protein |  |  |  |
| SAUSA300_2354 | *-* | Thiol:disulfide interchange protein DsbA |  |  | 2.03 |
| SAUSA300_2355 | *-* | Hypothetical protein |  |  |  |
| SAUSA300_2356 | *fmhA* | UDP-N-acetylmuramoylheptapeptide-glycine L-seryltransferase (EC 2.3.2.-) |  |  |  |
| SAUSA300_2357 | *-* | Cystine transport ATP-binding protein |  |  |  |
| SAUSA300_2358 | *-* | Cystine transport system permease protein |  |  |  |
| SAUSA300_2359 | *-* | Cystine-binding protein |  |  |  |
| SAUSA300_2360 | *-* | Multidrug resistance protein B |  |  |  |
| SAUSA300_2361 | *-* | Hypothetical protein |  |  |  |
| SAUSA300_2362 | *gpmA* | Phosphoglycerate mutase (EC 5.4.2.1) |  |  |  |
| SAUSA300_2363 | *-* | Cobalt-zinc-cadmium resistance protein czcD |  |  |  |
| SAUSA300_2364 | *sbi* | IgG-binding protein Sbi |  |  | 2.21 |
| SAUSA300_2365 | *hlgA* | Leukocidin S subunit |  |  |  |
| SAUSA300_2366 | *hlgC* | Leukocidin S subunit | -4.72 |  | -3.88 |
| SAUSA300_2367 | *hlgB* | Hypothetical protein | -2.62 |  | -2.36 |
| SAUSA300_2368 | *-* | BioX protein |  |  |  |
| SAUSA300_2369 | *-* | 6-carboxyhexanoate--CoA ligase (EC 6.2.1.14) |  |  |  |
| SAUSA300_2370 | *-* | 8-amino-7-oxononanoate synthase (EC 2.3.1.47) |  |  |  |
| SAUSA300_2371 | *bioB* | Biotin synthase (EC 2.8.1.6) |  |  |  |
| SAUSA300_2372 | *bioA* | Adenosylmethionine-8-amino-7-oxononanoate aminotransferase (EC 2.6.1.62) |  |  |  |
| SAUSA300_2373 | *bioD* | Dethiobiotin synthetase (EC 6.3.3.3) |  |  |  |
| SAUSA300_2374 | *-* | ABC transporter ATP-binding and permease protein |  |  |  |
| SAUSA300_2375 | *-* | ABC transporter ATP-binding and permease protein |  |  |  |
| SAUSA300_2376 | *-* | Monosaccharide translocase (flippase type) |  |  |  |
| SAUSA300_2377 | *-* | Glycerate kinase (EC 2.7.1.31) |  |  |  |
| SAUSA300_2378 | *-* | Hypothetical protein |  |  |  |
| SAUSA300_2379 | *-* | Hypothetical protein |  |  |  |
| SAUSA300_2380 | *-* | Transcriptional activator AarP |  |  |  |
| SAUSA300_2381 | *-* | Hypothetical protein |  |  |  |
| SAUSA300_2382 | *-* | Phosphoesterase |  |  |  |
| SAUSA300_2383 | *-* | D-serine/D-alanine/glycine transporter | -3.11 |  | -2.72 |
| SAUSA300_2384 | *-* | Na+/H+ antiporter NhaP |  |  |  |
| SAUSA300_2385 | *-* | Amino acid permease |  |  |  |
| SAUSA300_2386 | *-* | Beta-lactamase family protein |  |  |  |
| SAUSA300_2387 | *-* | dTDP-glucose 4,6-dehydratase (EC 4.2.1.46) |  |  |  |
| SAUSA300_2388 | *panE* | 2-dehydropantoate 2-reductase (EC 1.1.1.169) |  |  |  |
| SAUSA300_2389 | *-* | Multidrug resistance protein B |  |  |  |
| SAUSA300_2390 | *opuCd* | Glycine betaine transport system permease protein |  |  |  |
| SAUSA300_2391 | *opuCc* | Glycine betaine/carnitine/choline-binding protein |  |  |  |
| SAUSA300_2392 | *opuCb* | Glycine betaine/carnitine/choline transport system permease protein OpuCB |  |  |  |
| SAUSA300_2393 | *opuCa* | Glycine betaine transport ATP-binding protein |  |  |  |
| SAUSA300_2394 | *-* | 60 kDa chaperonin GroeL |  |  |  |
| SAUSA300_2395 | *-* | Amino acid permease |  |  |  |
| SAUSA300_2396 | *pnbA* | Para-nitrobenzyl esterase (EC 3.1.1.-) | -2.04 |  | -2.05 |
| SAUSA300_2397 | *-* | Chloramphenicol resistance protein |  |  |  |
| SAUSA300_2398 | *-* | ABC transporter permease protein |  |  |  |
| SAUSA300_2399 | *-* | ABC transporter ATP-binding protein |  |  |  |
| SAUSA300_2400 | *-* | Endoglucanase M (EC 3.2.1.4) |  |  |  |
| SAUSA300_2401 | *-* | RelE protein |  |  |  |
| SAUSA300_2402 | *-* | RelB protein |  |  |  |
| SAUSA300_2403 | *-* | Hypothetical protein |  |  |  |
| SAUSA300_2404 | *-* | Ferredoxin-dependent glutamate synthase (EC 1.4.7.1) |  |  |  |
| SAUSA300_2405 | *-* | Integral membrane protein |  |  |  |
| SAUSA300_2406 | *-* | Bicyclomycin resistance protein |  |  |  |
| SAUSA300_2407 | *-* | Nickel transport ATP-binding protein NikE |  |  |  |
| SAUSA300_2408 | *-* | Nickel transport ATP-binding protein NikD |  |  |  |
| SAUSA300_2409 | *-* | Nickel transport system permease protein NikC |  |  |  |
| SAUSA300_2410 | *-* | Nickel transport system permease protein NikB |  |  |  |
| SAUSA300_2411 | *opp-1A* | Nickel-binding protein |  |  |  |
| SAUSA300_2412 | *-* | Hypothetical protein | -2.34 |  |  |
| SAUSA300_2413 | *-* | Methyltransferase (EC 2.1.1.-) |  |  | -2.17 |
| SAUSA300_2414 | *-* | Diaminopimelate epimerase (EC 5.1.1.7) |  |  |  |
| SAUSA300_2415 | *-* | Hypothetical protein |  |  |  |
| SAUSA300_2416 | *-* | Hypothetical protein |  |  |  |
| SAUSA300_2417 | *-* | Aminobenzoyl-glutamate transport protein |  |  |  |
| SAUSA300_2418 | *-* | Transposase |  |  |  |
| SAUSA300_2419 | *-* | Hypothetical protein |  |  |  |
| SAUSA300_2420 | *-* | Hypothetical protein |  |  |  |
| SAUSA300_2421 | *-* | Hypothetical protein |  |  |  |
| SAUSA300_2422 | *-* | Short chain dehydrogenase |  |  |  |
| SAUSA300_2423 | *-* | Hypothetical protein |  |  |  |
| SAUSA300_2424 | *-* | membrane lipoprotein |  |  |  |
| SAUSA300_2425 | *-* | Hypothetical protein |  |  |  |
| SAUSA300_2426 | *-* | Hypothetical cytosolic protein |  |  |  |
| SAUSA300_2428 | *-* | membrane lipoprotein |  |  |  |
| SAUSA300_2429 | *-* | Hypothetical protein |  |  |  |
| SAUSA300_2430 | *-* | membrane lipoprotein |  |  |  |
| SAUSA300_2431 | *-* | DNA/RNA helicase (DEAD/DEAH box family) |  |  |  |
| SAUSA300_2432 | *-* | 7,8-dihydro-8-oxoguanine-triphosphatase (EC 3.6.1.-) |  |  |  |
| SAUSA300_2433 | *-* | Phosphoglucomutase (EC 5.4.2.2) / Phosphomannomutase (EC 5.4.2.8) |  |  |  |
| SAUSA300_2434 | *-* | Macrolide-efflux protein |  |  |  |
| SAUSA300_2435 | *-* | Beta-N-acetylhexosaminidase (EC 3.2.1.52) |  |  |  |
| SAUSA300_2436 | *-* | Beta-N-acetylhexosaminidase (EC 3.2.1.52) | 2.13 |  | 2.06 |
| SAUSA300_2437 | *sarT* | STAPHYLOCOCCAL ACCESSORY REGULATOR A |  |  |  |
| SAUSA300_2438 | *sarU* | STAPHYLOCOCCAL ACCESSORY REGULATOR A |  |  |  |
| SAUSA300_2439 | *galU* | UTP--glucose-1-phosphate uridylyltransferase (EC 2.7.7.9) |  |  |  |
| SAUSA300_2440 | *fnbB* | Fibronectin-binding protein |  |  |  |
| SAUSA300_2441 | *fnbA* | Fibronectin-binding protein |  |  |  |
| SAUSA300_2442 | *gntP* | Gluconate permease |  |  |  |
| SAUSA300_2443 | *gntK* | Gluconokinase (EC 2.7.1.12) |  | 3.11 |  |
| SAUSA300_2444 | *gntR* | Gluconate operon transcriptional repressor |  | 2.82 |  |
| SAUSA300_2445 | *-* | Transcriptional regulator, MerR family | -2.21 |  |  |
| SAUSA300_2446 | *-* | GTP pyrophosphokinase (EC 2.7.6.5) | -2.42 |  |  |
| SAUSA300_2447 | *-* | Hypothetical protein |  |  |  |
| SAUSA300_2448 | *-* | Integral membrane protein | -8.49 | -2.12 | -2.72 |
| SAUSA300_2449 | *-* | Transporter, MFS superfamily |  |  |  |
| SAUSA300_2450 | *-* | DedA family protein |  |  |  |
| SAUSA300_2451 | *-* | Bicyclomycin resistance protein |  |  |  |
| SAUSA300_2452 | *-* | Transcriptional regulator, MarR family |  |  |  |
| SAUSA300_2453 | *-* | Lantibiotic transport ATP-binding protein | 5.65 |  | 11.95 |
| SAUSA300_2454 | *-* | ABC transporter ATP-binding protein | 4.09 |  | 10.85 |
| SAUSA300_2455 | *-* | Fructose-1,6-bisphosphatase (EC 3.1.3.11) | -4.90 |  | -3.34 |
| SAUSA300_2456 | *-* | Hypothetical membrane spanning protein |  |  |  |
| SAUSA300_2457 | *-* | Carboxylesterase (EC 3.1.1.1) |  |  |  |
| SAUSA300_2458 | *-* | Glyoxalase family protein |  |  |  |
| SAUSA300_2459 | *-* | Transcriptional regulator, MarR family | 2.47 |  | 2.03 |
| SAUSA300_2460 | *-* | Acetyltransferase (EC 2.3.1.-) |  |  |  |
| SAUSA300_2461 | *-* | Glyoxalase family protein |  | 2.05 |  |
| SAUSA300_2462 | *frp* | NAD(P)H-dependent quinone reductase (EC 1.-.-.-) | 2.02 |  |  |
| SAUSA300_2463 | *ddh* | D-2-hydroxyacid dehydrogenase (EC 1.1.1.-) |  |  |  |
| SAUSA300_2464 | *-* | Hydrolase (HAD superfamily) |  |  |  |
| SAUSA300_2465 | *-* | ABC transporter ATP-binding protein |  |  |  |
| SAUSA300_2466 | *-* | Hypothetical membrane spanning protein |  |  |  |
| SAUSA300_2467 | *srtA* | Sortase |  |  |  |
| SAUSA300_2468 | *-* | Phosphinothricin N-acetyltransferase (EC 2.3.1.-) |  |  |  |
| SAUSA300_2469 | *sdaAA* | L-serine dehydratase alpha subunit (EC 4.3.1.17) |  |  |  |
| SAUSA300_2470 | *sdaAB* | L-serine dehydratase beta subunit (EC 4.3.1.17) | -2.37 |  | -2.52 |
| SAUSA300_2471 | *-* | Transcriptional regulator pfoR | -3.44 |  | -2.46 |
| SAUSA300_2472 | *-* | Transporter, drug/metabolite exporter family |  |  |  |
| SAUSA300_2473 | *-* | Esterase (EC 3.1.1.-) |  |  |  |
| SAUSA300_2474 | *-* | Thioredoxin |  |  |  |
| SAUSA300_2475 | *-* | Thioesterase (EC 3.1.2.-) |  |  |  |
| SAUSA300_2476 | *ptsG* | PTS system, glucose-specific IIABC component (EC 2.7.1.69) |  | 2.70 | -2.07 |
| SAUSA300_2477 | *cidC* | Pyruvate dehydrogenase [cytochrome] (EC 1.2.2.2) |  |  |  |
| SAUSA300_2478 | *cidB* | Murein hydrolase export regulator |  |  |  |
| SAUSA300_2479 | *cidA* | Murein hydrolase exporter |  |  |  |
| SAUSA300_2480 | *-* | Hypothetical protein |  |  |  |
| SAUSA300_2481 | *-* | Hypothetical cytosolic protein |  |  |  |
| SAUSA300_2482 | *-* | SceB precursor |  |  |  |
| SAUSA300_2483 | *-* | 3-hydroxy-3-methylglutaryl-coenzyme A reductase (EC 1.1.1.34) / 3-hydroxy-3-methylglutaryl-coenzyme A reductase (EC 1.1.1.88) |  |  |  |
| SAUSA300_2484 | *-* | Hydroxymethylglutaryl-CoA synthase (EC 2.3.3.10) |  |  |  |
| SAUSA300_2485 | *-* | O6-methylguanine-DNA methyltransferase (EC 2.1.1.63) |  |  |  |
| SAUSA300_2486 | *-* | ATP-dependent endopeptidase clp ATP-binding subunit ClpL |  |  |  |
| SAUSA300_2487 | *feoB* | Ferrous iron transport protein B |  |  |  |
| SAUSA300_2488 | *feoA* | Ferrous iron transport protein A |  |  | -2.09 |
| SAUSA300_2489 |  |  |  |  |  |
| SAUSA300_2490 | *-* | Transcriptional regulator, TetR family |  |  |  |
| SAUSA300_2491 | *-* | Delta-1-pyrroline-5-carboxylate dehydrogenase (EC 1.5.1.12) | -2.09 |  | -2.13 |
| SAUSA300_2492 | *-* | O-acetyltransferase (cell wall biosynthesis) (EC 2.3.1.-) |  |  |  |
| SAUSA300_2493 | *-* | Hypothetical protein |  |  | -9.25 |
| SAUSA300_2494 | *-* | Copper-exporting ATPase (EC 3.6.3.4) |  | 2.16 |  |
| SAUSA300_2495 | *-* | Hypothetical protein |  |  |  |
| SAUSA300_2496 | *-* | D-2-hydroxyacid dehydrogenase (EC 1.1.1.-) |  |  |  |
| SAUSA300_2497 | *-* | Aspartate aminotransferase (EC 2.6.1.1) |  |  |  |
| SAUSA300_2498 | *crtN* | Dehydrosqualene desaturase (EC 1.3.99.-) |  |  |  |
| SAUSA300_2499 | *crtM* | dehydrosqualene synthase (EC 2.5.1.-) |  |  |  |
| SAUSA300_2500 | *-* | HpnB protein |  |  |  |
| SAUSA300_2501 | *-* | Phytoene desaturase (EC 1.14.99.-) |  |  |  |
| SAUSA300_2502 | *-* | Hypothetical protein |  |  |  |
| SAUSA300_2503 | *-* | Secretory antigen precursor SsaA |  |  |  |
| SAUSA300_2504 | *-* | Acyltransferase family |  |  |  |
| SAUSA300_2505 | *-* | Acetyltransferase, GNAT family |  |  |  |
| SAUSA300_2506 | *isaA* | Hypothetical protein |  |  |  |
| SAUSA300_2507 | *-* | Hypothetical membrane spanning protein |  |  |  |
| SAUSA300_2508 | *-* | Hypothetical protein |  |  |  |
| SAUSA300_2509 | *-* | Transcriptional regulator, TetR family |  |  |  |
| SAUSA300_2510 | *-* | Hypothetical protein |  |  |  |
| SAUSA300_2511 | *-* | Hypothetical cytosolic protein | 2.21 |  |  |
| SAUSA300_2512 | *-* | Glyoxalase family protein | 7.75 | 6.27 |  |
| SAUSA300_2513 | *-* | putative nucleoside-diphosphate-sugar epimerases | 10.22 | 10.20 |  |
| SAUSA300_2514 | *-* | Hypothetical protein | 10.01 | 11.95 |  |
| SAUSA300_2515 | *-* | Transcriptional regulator, TetR family | 8.56 | 8.77 |  |
| SAUSA300_2516 | *-* | Short chain dehydrogenase | 9.34 | 11.52 |  |
| SAUSA300_2517 | *-* | 5-carboxyvanillic acid decarboxylase (EC 4.1.1.-) | 2.03 | 2.89 |  |
| SAUSA300_2518 | *-* | Esterase/Lipase (EC 3.1.-.-) |  |  |  |
| SAUSA300_2519 | *-* | Low-affinity zinc transport protein |  |  |  |
| SAUSA300_2520 | *-* | Ferrous iron transport protein B |  |  |  |
| SAUSA300_2521 | *-* | Oxidoreductase (EC 1.1.1.-) |  |  |  |
| SAUSA300_2522 | *-* | Hypothetical cytosolic protein |  |  | -2.34 |
| SAUSA300_2523 | *-* | Hypothetical protein | -2.12 |  | -2.43 |
| SAUSA300_2524 | *-* | Hypothetical protein |  |  | -2.31 |
| SAUSA300_2525 | *-* | Fructosamine kinase family protein |  |  |  |
| SAUSA300_2526 | *pyrD* | Dihydroorotate dehydrogenase (EC 1.3.3.1) |  |  |  |
| SAUSA300_2527 | *-* | Hypothetical protein |  |  |  |
| SAUSA300_2528 | *-* | Hypothetical cytosolic protein |  |  |  |
| SAUSA300_2529 | *-* | PhnB protein |  |  |  |
| SAUSA300_2530 | *-* | Transcriptional regulator, TetR family |  |  |  |
| SAUSA300_2531 | *-* | X-Pro dipeptidyl-peptidase (S15) family protein |  |  |  |
| SAUSA300_2532 | *panD* | Aspartate 1-decarboxylase (EC 4.1.1.11) |  |  |  |
| SAUSA300_2533 | *panC* | Pantoate--beta-alanine ligase (EC 6.3.2.1) |  |  |  |
| SAUSA300_2534 | *panB* | 3-methyl-2-oxobutanoate hydroxymethyltransferase (EC 2.1.2.11) |  |  |  |
| SAUSA300_2535 | *panE* | 2-dehydropantoate 2-reductase (EC 1.1.1.169) |  |  |  |
| SAUSA300_2536 | *budA* | Alpha-acetolactate decarboxylase (EC 4.1.1.5) |  |  |  |
| SAUSA300_2537 | *-* | L-lactate dehydrogenase (EC 1.1.1.27) |  |  |  |
| SAUSA300_2538 | *-* | Amino acid permease |  |  |  |
| SAUSA300_2539 | *-* | 4-aminobutyrate aminotransferase (EC 2.6.1.19) |  |  |  |
| SAUSA300_2540 | *-* | Fructose-bisphosphate aldolase (EC 4.1.2.13) |  |  |  |
| SAUSA300_2541 | *mqo* | Malate:quinone oxidoreductase (EC 1.1.99.16) |  |  |  |
| SAUSA300_2542 | *-* | Acetyl-coenzyme A synthetase (EC 6.2.1.1) |  |  |  |
| SAUSA300_2543 | *-* | Signal transduction protein TRAP |  |  |  |
| SAUSA300_2544 | *-* | Hypothetical cytosolic protein |  |  |  |
| SAUSA300_2545 | *betA* | Choline dehydrogenase (EC 1.1.99.1) |  |  |  |
| SAUSA300_2546 | *betB* | Betaine aldehyde dehydrogenase (EC 1.2.1.8) |  | 2.21 | -2.48 |
| SAUSA300_2547 | *-* | Transcriptional regulator, ArsR family |  |  |  |
| SAUSA300_2548 | *-* | Zinc metallohydrolase, glyoxalase ii family |  |  |  |
| SAUSA300_2549 | *bccT* | Choline transport protein |  |  |  |
| SAUSA300_2550 | *nrdG* | Anaerobic ribonucleoside-triphosphate reductase activating protein (EC 1.97.1.4) |  |  |  |
| SAUSA300_2551 | *nrdD* | Anaerobic ribonucleoside-triphosphate reductase (EC 1.17.4.2) |  |  |  |
| SAUSA300_2552 | *-* | Hypothetical protein |  |  |  |
| SAUSA300_2553 | *-* | Precorrin-2 dehydrogenase (EC 1.3.1.76) / Sirohydrochlorin ferrochelatase (EC 4.99.1.4) | 2.20 |  |  |
| SAUSA300_2554 | *-* | Sulfite reductase [NADPH] flavoprotein alpha-component (EC 1.8.1.2) | 2.51 |  |  |
| SAUSA300_2555 | *-* | Glutathione peroxidase (EC 1.11.1.9) |  |  |  |
| SAUSA300_2556 | *-* | ABC transporter permease protein |  |  |  |
| SAUSA300_2557 | *-* | ABC transporter ATP-binding protein |  |  |  |
| SAUSA300_2558 | *-* | Two-component sensor histidine kinase (EC 2.7.3.-) |  |  |  |
| SAUSA300_2559 | *-* | Two-component response regulator |  |  |  |
| SAUSA300_2560 | *-* | Hypothetical protein |  |  |  |
| SAUSA300_2561 | *phoB* | Alkaline phosphatase (EC 3.1.3.1) |  |  |  |
| SAUSA300_2562 | *-* | Hypothetical protein |  |  |  |
| SAUSA300_2563 | *-* | Transcriptional regulator, MarR family |  |  |  |
| SAUSA300_2564 | *estA* | Acetyl esterase (EC 3.1.1.-) |  |  |  |
| SAUSA300_2565 | *clfB* | Hypothetical protein |  | -2.38 | 3.88 |
| SAUSA300_2566 | *arcR* | Transcription regulator, Crp family |  |  |  |
| SAUSA300_2567 | *arcC* | Carbamate kinase (EC 2.7.2.2) |  |  |  |
| SAUSA300_2568 | *arcD* | Arginine/ornithine antiporter |  |  |  |
| SAUSA300_2569 | *arcB* | Ornithine carbamoyltransferase (EC 2.1.3.3) |  |  |  |
| SAUSA300_2570 | *arcA* | Arginine deiminase (EC 3.5.3.6) |  |  | -2.98 |
| SAUSA300_2571 | *argR* | Arginine repressor, argR |  |  |  |
| SAUSA300_2572 | *aur* | Zinc metalloproteinase aureolysin (EC 3.4.24.29) | -2.73 |  |  |
| SAUSA300_2573 | *isaB* | Hypothetical protein |  |  |  |
| SAUSA300_2574 | *-* | Hypothetical exported protein |  |  |  |
| SAUSA300_2575 | *-* | Transcription antiterminator, BglG family |  |  |  |
| SAUSA300_2576 | *-* | PTS system, mannose-specific IIAB component (EC 2.7.1.69) / PTS system, mannose-specific IIC component (EC 2.7.1.69) | -2.57 |  |  |
| SAUSA300_2577 | *manA* | Mannose-6-phosphate isomerase (EC 5.3.1.8) | -2.80 |  |  |
| SAUSA300_2578 | *-* | Hypothetical membrane spanning protein |  |  |  |
| SAUSA300_2579 | *-* | N-acetylmuramidase (EC 3.2.1.17) | -2.14 |  |  |
| SAUSA300_2580 | *-* | N-carbamoylsarcosine amidase (EC 3.5.1.59) |  |  |  |
| SAUSA300_2581 | *-* | Hypothetical protein |  |  |  |
| SAUSA300_2582 | *-* | surface protein Pls |  |  |  |
| SAUSA300_2583 | *-* | Poly(Glycerol-phosphate) alpha-glucosyltransferase (EC 2.4.1.52) |  |  |  |
| SAUSA300_2584 | *-* | Protein translocase subunit SecA |  |  |  |
| SAUSA300_2585 | *-* | Hypothetical cytosolic protein | -3.01 |  |  |
| SAUSA300_2586 | *-* | Hypothetical cytosolic protein | -3.73 |  |  |
| SAUSA300_2587 | *-* | Hypothetical protein | -3.71 |  |  |
| SAUSA300_2588 | *secY* | Protein translocase subunit secY | -3.49 |  |  |
| SAUSA300_2589 | *-* | Cell surface protein | -2.02 |  |  |
| SAUSA300_2590 | *-* | FAD-dependent oxidoreductase (EC 1.-.-.-) |  |  |  |
| SAUSA300_2591 | *-* | Hypothetical protein |  |  |  |
| SAUSA300_2592 | *-* | Hypothetical protein |  |  |  |
| SAUSA300_2593 | *-* | FAD-dependent oxidoreductase (EC 1.-.-.-) |  |  |  |
| SAUSA300_2594 | *msrA* | Peptide methionine sulfoxide reductase (EC 1.8.4.11) |  |  |  |
| SAUSA300_2595 | *-* | Acetyltransferase, GNAT family |  |  |  |
| SAUSA300_2596 | *cap1C* | Phosphotyrosine-protein phosphatase (capsular polysaccharide biosynthesis) (EC 3.1.3.48) |  |  |  |
| SAUSA300_2597 | *cap1B* | Tyrosine-protein kinase (capsular polysaccharide biosynthesis) |  |  |  |
| SAUSA300_2598 | *cap1A* | Chain length regulator (capsular polysaccharide biosynthesis) |  |  |  |
| SAUSA300_2599 | *tetR* | Transcriptional regulator IcaR |  |  |  |
| SAUSA300_2600 | *icaA* | N-acetylglucosaminyltransferase (EC 2.4.1.-) |  |  |  |
| SAUSA300_2601 | *icaB* | Polysaccharide deacetylase |  |  |  |
| SAUSA300_2602 | *icaC* | Intercellular adhesion protein IcaC |  |  |  |
| SAUSA300_2603 | *lip* | Lipase (EC 3.1.1.3) | -12.97 |  | -20.97 |
| SAUSA300_2604 | *-* | Hypothetical protein |  |  |  |
| SAUSA300_2605 | *hisIE* | Phosphoribosyl-AMP cyclohydrolase (EC 3.5.4.19) / Phosphoribosyl-ATP diphosphatase (EC 3.6.1.31) |  |  |  |
| SAUSA300_2606 | *hisF* | Imidazole glycerol phosphate synthase, cyclase subunit (EC 4.1.3.-) |  |  |  |
| SAUSA300_2607 | *hisA* | 1-(5-phosphoribosyl)-5-[(5-phosphoribosylamino)methylideneamino] imidazole-4-carboxamide isomerase (EC 5.3.1.16) |  |  |  |
| SAUSA300_2608 | *hisH* | Imidazole glycerol phosphate synthase, glutamine amidotransferase subunit (EC 2.4.2.-) |  |  |  |
| SAUSA300_2609 | *hisB* | Imidazoleglycerol-phosphate dehydratase (EC 4.2.1.19) |  | -2.26 |  |
| SAUSA300_2610 | *hisC* | Histidinol-phosphate aminotransferase (EC 2.6.1.9) |  | -2.80 | 2.27 |
| SAUSA300_2611 | *hisD* | Histidinol dehydrogenase (EC 1.1.1.23) |  | -2.47 | 2.06 |
| SAUSA300_2612 | *hisG* | ATP phosphoribosyltransferase (EC 2.4.2.17) |  |  |  |
| SAUSA300_2613 | *hisZ* | Hypothetical cytosolic protein |  | -2.57 |  |
| SAUSA300_2614 | *-* | Hypothetical protein |  |  |  |
| SAUSA300_2615 | *-* | Ribosomal-protein-alanine acetyltransferase (EC 2.3.1.128) |  |  |  |
| SAUSA300_2616 | *-* | Cobalt transport protein cbiQ |  |  |  |
| SAUSA300_2617 | *-* | Cobalt transport ATP-binding protein cbiO |  |  |  |
| SAUSA300_2618 | *-* | Hypothetical protein |  |  |  |
| SAUSA300_2619 | *-* | Hypothetical cytosolic protein |  |  |  |
| SAUSA300_2620 | *-* | Hypothetical exported protein |  |  |  |
| SAUSA300_2621 | *-* | Antibiotic-induced protein, Drp35 | -2.35 |  | -2.63 |
| SAUSA300_2622 | *-* | Rhodanese-related sulfurtransferases | 3.68 |  |  |
| SAUSA300_2623 | *pcp* | Pyrrolidone-carboxylate peptidase (EC 3.4.19.3) |  |  |  |
| SAUSA300_2624 | *-* | Hypothetical protein |  |  |  |
| SAUSA300_2625 | *-* | Transcriptional regulator, PadR family |  |  |  |
| SAUSA300_2626 | *-* | Hypothetical protein | 2.06 |  |  |
| SAUSA300_2627 | *-* | 2-oxoglutarate/malate translocator |  |  |  |
| SAUSA300_2628 | *rarD* | Chloramphenicol-sensitive protein rarD |  |  |  |
| SAUSA300_2629 | *-* | Prolyl-tRNA synthetase |  |  |  |
| SAUSA300_2630 | *nixA* | High-affinity nickel transport protein |  |  |  |
| SAUSA300_2631 | *-* | Hypothetical protein |  |  |  |
| SAUSA300_2632 | *-* | Hypothetical membrane spanning protein |  |  |  |
| SAUSA300_2633 | *-* | Bacitracin transport ATP-binding protein ytsC |  |  |  |
| SAUSA300_2634 | *-* | ABC transporter permease protein |  |  |  |
| SAUSA300_2635 | *-* | Hypothetical protein |  |  |  |
| SAUSA300_2636 | *-* | Transposase |  |  |  |
| SAUSA300_2637 | *-* | Hypothetical protein |  |  |  |
| SAUSA300_2639 | *-* | Cold shock protein |  |  |  |
| SAUSA300_2640 | *-* | Transcriptional regulator |  | -2.33 | 3.80 |
| SAUSA300_2641 | *-* | Permease |  |  | 2.02 |
| SAUSA300_2642 | *-* | Permease | 2.48 |  | 2.53 |
| SAUSA300_2642 | *-* | Permease |  |  |  |
| SAUSA300_2643 | *-* | Chromosome partitioning protein ParB |  |  |  |
| SAUSA300_2644 | *gidB* | Methyltransferase GidB (EC 2.1.-.-) |  |  |  |
| SAUSA300_2645 | *gidA* | Glucose inhibited division protein A |  |  |  |
| SAUSA300_2646 | *trmE* | tRNA (5-carboxymethylaminomethyl-2-thiouridylate) synthase |  |  |  |
| SAUSA300_2647 | *rnpA* | Ribonuclease P protein component (EC 3.1.26.5) |  |  |  |
| SAUSA300_2648 | *rpmH* | LSU ribosomal protein L34P |  |  |  |

Transcriptional profile of USA300 LAC treated with savirin (5 µg ml^-1^) vs. vehicle, Δ*agr* USA300 LAC treated with savirin (5 µg ml^-1^) vs. vehicle, and *Δagr* USA 300 LAC vs*. agr*+ after 5 hrs incubation with 50 nM AIP1. Microarray results are presented as the mean fold-change of 3 separate experiments. Data that met criteria for differentially-expressed genes (P value <0.05; >2-fold change) are included. Blank indicates no change; red values are upregulated and blue values are downregulated.
