# Supplementary material for: Selective Chemical Inhibition of agr Quorum Sensing in Staphylococcus aureus Promotes Host Defense with Minimal Impact on Resistance
Source: PLoS Pathog. 2014 Jun 12;10(6):e1004174. doi: 10.1371/journal.ppat.1004174 (PMC4055767; doi:10.1371/journal.ppat.1004174)
Supplement: Table S2 — Transcripts upregulated by savirin in LAC agr+ and Δagr. (DOC) [file ppat.1004174.s009.doc]

**Table S2.** Transcripts upregulated by savirin in LAC *agr*+ and *Δagr*

| **ORF** | **Gene** |  | | **Description** | ***agr*+** | ***Δagr*** |
| --- | --- | --- | --- | --- | --- | --- |
| SAUSA300_0334 | *-* |  | Transcriptional regulator, MarR family* | | 4.51 | 2.28 |
| SAUSA300_0335 | *-* |  | Na+ driven multidrug efflux pump* | | 3.71 | 2.77 |
| SAUSA300_1283 | *phoU* |  | Phosphate-binding protein | | 1.72 | 1.91 |
| SAUSA300_1331 | *ald* |  | Alanine dehydrogenase (EC 1.4.1.1) | | 5.62 | 2.86 |
| SAUSA300_2105 | *glmS* |  | PTS system, mannitol-specific IIBC component (EC 2.7.1.69) | | 4.07 | 2.73 |
| SAUSA300_2106 | *-* |  | Transcription antiterminator, BglG family / PTS system, mannitol (Cryptic)-specific IIA component (EC 2.7.1.69) | | 4.02 | 2.94 |
| SAUSA300_2107 | *-* |  | PTS system, mannitol-specific IIA component (EC 2.7.1.69) | | 2.31 | 2.15 |
| SAUSA300_2108 | *mtlD* |  | Mannitol-1-phosphate 5-dehydrogenase (EC 1.1.1.17) | | 2.29 | 2.39 |
| SAUSA300_2298 | *-* |  | Multidrug resistance protein B* | | 5.78 | 6.95 |
| SAUSA300_2299 | *-* |  | Multidrug resistance protein A* | | 10.84 | 8.45 |
| SAUSA300_2306 | *-* |  | ABC transporter ATP-binding protein | | 3.52 | 2.05 |
| SAUSA300_2307 | *-* |  | ABC transporter permease protein | | 2.65 | 2.26 |
| SAUSA300_2321 | *-* |  | Phage infection protein | | 30.75 | 30.51 |
| SAUSA300_2512 | *-* |  | Glyoxalase family protein | | 7.75 | 6.27 |
| SAUSA300_2513 | *-* |  | putative nucleoside-diphosphate-sugar epimerases | | 10.22 | 10.20 |
| SAUSA300_2514 | *-* |  | Hypothetical protein | | 10.01 | 11.95 |
| SAUSA300_2515 | *-* |  | Transcriptional regulator, TetR family* | | 8.56 | 8.77 |
| SAUSA300_2516 | *-* |  | Short chain dehydrogenase | | 9.34 | 11.52 |
| SAUSA300_2517 | *-* |  | 5-carboxyvanillic acid decarboxylase (EC 4.1.1.-) | | 2.03 | 2.89 |

*ORFs with potential roles in drug efflux and/or resistance
